# Reconstruction of *Escherichia coli *transcriptional regulatory networks via regulon-based associations

**DOI:** 10.1186/1752-0509-3-39

**Published:** 2009-04-14

**Authors:** Hossein Zare, Dipen Sangurdekar, Poonam Srivastava, Mostafa Kaveh, Arkady Khodursky

**Affiliations:** 1 Department of Electrical and Computer Engineering, The University of Minnesota, Minneapolis, MN, USA; 2 Department of Biochemistry, Biophysics and Molecular Biology, The University of Minnesota, St. Paul, MN, USA

## Abstract

**Background:**

Network reconstruction methods that rely on covariance of expression of transcription regulators and their targets ignore the fact that transcription of regulators and their targets can be controlled differently and/or independently. Such oversight would result in many erroneous predictions. However, accurate prediction of gene regulatory interactions can be made possible through modeling and estimation of transcriptional activity of groups of co-regulated genes.

**Results:**

Incomplete regulatory connectivity and expression data are used here to construct a consensus network of transcriptional regulation in *Escherichia coli *(*E. coli*). The network is updated via a covariance model describing the activity of gene sets controlled by common regulators. The proposed model-selection algorithm was used to annotate the likeliest regulatory interactions in *E. coli *on the basis of two independent sets of expression data, each containing many microarray experiments under a variety of conditions. The key regulatory predictions have been verified by an experiment and literature survey. In addition, the estimated activity profiles of transcription factors were used to describe their responses to environmental and genetic perturbations as well as drug treatments.

**Conclusion:**

Information about transcriptional activity of documented co-regulated genes (a core regulon) should be sufficient for discovering new target genes, whose transcriptional activities significantly co-vary with the activity of the core regulon members. Our ability to derive a highly significant consensus network by applying the regulon-based approach to two very different data sets demonstrated the efficiency of this strategy. We believe that this approach can be used to reconstruct gene regulatory networks of other organisms for which partial sets of known interactions are available.

## 1 Background

One of the goals of systems biology is to elucidate functionally relevant regulatory interactions[1,2]. Since changes in gene expression are in part determined by such interactions between regulators and their target genes, genome-wide expression data can be effectively used to impute regulatory transcriptional networks. One approach, which is based on the assumption that functionally related genes should show similar transcriptional activity across time points or different environmental conditions, uses clustering to identify sets of genes with similar expression profiles [3] and regulator-specific network modules [4]. However, the utility of this pioneering approach was limited by the greedy nature of clustering and lack of a quantitative measure of interactions between genes.

To overcome these limitations more complex statistical and mathematical models have been proposed, which are briefly summarized below and in Table 1. The first class of gene regulatory network models considers a gene regulatory network as a linear and time continuous system, with the transcriptional activity of genes described by a time continuous dynamical system of first order differential equations [5-7] or by stochastic dynamical equations, a framework based on a state space model [8] or Dynamic Bayesian network [9,10]. Boolean gene regulatory networks [11-13] are particular cases of dynamic networks which assume that the time and states of the system are discrete. Genes are the network nodes which are in one of two binary on/off states, which is the boolean function of the each node inputs' states. These methods often use time series data collected over a small number of time points, compared to the large number of genes, which results in an under-determined problem [14,15]. The algorithms falling in the second category, including relevance networks [16-18], Bayesian networks [19] and graphical Gaussian models (GGM) [20,21], impute gene networks by establishing connectivity (edges) between genes based on the dependencies in their expression profiles. The GGMs and relevance networks model conditional and marginal independencies, respectively, among the gene pairs. The application of the GGMs is limited to the gene networks with the number of experimental measurements significantly greater than the number of genes. Similarly, the relevance network algorithm, which uses mutual information between genes and treats gene expression levels across different conditions as ensembles of single random variables, can capture the condition-specific activity of the genes only when the sample size is very large.

**Table 1:** Models of Gene Regulatory Networks

Gene Network Methods	Brief Descriptions
Differential Equation Models, [5-7]	Require time series data, limited to small-scale networks, quantify interactions, associations are based on mRNA levels

Boolean Networks, [11-13]	Require time series data, limited to small scale networks, associations are based on mRNA levels

Bayesian Networks and Graphical Models, [19-21]	Measure the marginal and conditional dependencies among genes, associations are based on mRNA levels, learning the structure of large scale networks is highly complex

Relevance Networks [16-18]	Measure the linear or nonlinear correlations among genes, associations are based on mRNA levels and may not be direct.

Matrix Decomposition, [27,28]	Require complete knowledge of a potential connectivity network, refine and quantify the network using gene expression data

Supervised Methods, [35] and this paper	Require partial knowledge of the connectivity network, association are based on activity profile of transcription factors

Yet another approach to inferring gene regulatory networks is through non-greedy decomposition of gene expression data matrices to uncover hidden, often overlapping, regulatory signals and transcriptional connectivity patterns. Since the data does not have to be a time series, one can collect data for as many different experiments as possible and combine them to increase the sample size and prevent the problem of under-determination. Principal component analysis (PCA) [22] or singular value decomposition [23-25] and independent component analysis (ICA)[26] can be used to determine the low-dimensional representation of the data through decomposing the original data into a few regulatory signals which explain most of the data. However, the orthogonality assumption of PCA and the statistical independency assumption of ICA place methodological constraints on biological signals. Network component analysis (NCA) [27] is another matrix decomposition method, devised specifically for gene expression data, which takes advantage of available knowledge about the connectivity pattern of the network. The NCA method and a two-stage matrix decomposition model in [28] assume that the connectivity matrix is fully known, and, therefore, it does not predict any new interaction among the genes and transcription factors.

In this paper we introduce a new and highly efficient approach to the problem of gene network inference through a simple model selection algorithm. The algorithm uses gene expression data and documented transcriptional connections to predict a more complete structure of the transcriptional network. The method is based on covariance models of the co-regulated gene sets. We demonstrate that this approach can uncover previously uncharacterized regulatory interactions and simultaneously estimate the activity profiles of regulators from the corresponding covariance matrices. Comparison with the relevance network and GGM algorithms on two different data sets demonstrated the advantage of using the "gene"-"regulon" associations (the present method) over the "gene"-"regulator" associations (relevance networks and GGM). The proposed method outperformed both algorithms on both data sets at a very high significance margin with a recall value of more than 62% and precision value of 64%. We confirmed some of the predicted interactions by experiment and literature mining.

## 2 Results and Discussions

### 2.1 Data sets

Microarray gene expression data for more than 100 arrays representing 46 biologically distinct conditions have been used to reconstruct the underlying large scale transcriptional regulatory network of *E. coli*. The conditions covered a spectrum of environmental and genetic perturbations and drug treatments. The environmental perturbations, in addition to those described in [29] (Data set is available at NCBI Gene Expression Omnibus (GEO) with accession number: GSE4357-GSE4380), included different amino acid and nucleotide additions and limitations (NCBI GEO Series accession number: GSE15409). After filtering the collected expression data by a series of different criteria (removing genes with low variance expressions across conditions, with small absolute values and with low entropy profiles across conditions), expression measurements of 3658 genes were used in this study.

A second data set used in our study was published in [17] and was obtained from Many Microbe Microarray database (*M*^3*D*^) web site . This set contained expression levels of *E. coli *genes across 524 arrays which resolved into 189 different experimental conditions. It should be noted here that not only do these two data sets cover very different genetic or environmental perturbations, but they also have been collected on two different microarray platforms: cDNA microarrays and Affymatrix Genechips.

The connectivity matrix obtained from RegulonDB [30]. This connectivity matrix contained the connectivity pattern between 1210 genes (we only considered genes having expression measurements in our data set) and 137 transcription factors having more than two known targets.

### 2.2 Algorithm

For each transcription factor, the core regulon, consisting of the set of known targets, is identified from the known connectivity matrix described above. This information is used to learn a covariance model for each transcription factor. The covariance matrices are estimated from the expression data of genes assigned to regulons. The gene expression measurements of the group of *K *genes controlled by a TF are treated as *K *realizations of an independent random variable with the same distribution and, therefore, the weighted sample covariance matrix estimation method was applied to approximate the TF's model covariance matrix. Higher weight is given to those gene samples that are exclusively controlled by the TF. The bootstrap procedure is incorporated to increase the estimation accuracy of covariance model for those TFs with the low number of known targets. Then the algorithm computes the Gene-Regulon association score for each gene-regulon pair using the likelihood function defined in the Method Section. Finally, the activity profiles of transcription factors are estimated using the eigenvalue decomposition procedure. The regulon-based methodology assumes that the expressions of target genes vary with the activity of their regulator, which does not have to be determined solely by its transcript levels but can be a combination of latent factors including abundance, modification status, interaction with low molecular weight effectors or other proteins.

### 2.3 Performance Comparison

To assess the relative performance of our algorithm, we compared our algorithm with the relevance network method developed in [17], and with GGM method presented in [21]. We did not include other relevance network methods such as [16,18] and Bayesian networks [19] because the method in [17] outperforms them.

Since we were interested in transcriptional regulatory interactions (interactions between transcription factors and their target genes), we built a network by comparing scores for all possible pairs of transcription factor-gene targets. To make a fair comparison, in each method for each gene we ranked the regulators based on their association scores with that gene. A regulator which has the maximum association score with a gene was assigned to that gene. The second regulator was assigned to the gene if the corresponding association score was greater than the minimum of the association scores of the genes assigned to that regulator in the first round. This procedure was repeated and assignments were made, if warranted by the association score, for the lower ranking regulators as well. This procedure is different from those that use a global threshold parameter to select the edges. Assignment of regulators based on a global threshold for all TFs results in a very limited number of predictions, although with a good precision. Due to the large scale of the data, it is reasonable to assume that there should be at least one regulator that can explain the gene expression data of each gene. Although, this association may not be discovered through the dependency between the expression level of the gene and the mRNA level of a gene coding for the transcription factor, it may be discovered using gene-regulon based association, and this is what we would like to demonstrate.

We compared the prediction results of these three algorithms over the set of known interactions in RegulonDB database [30], which was compiled to a binary matrix of interactions between 1210 genes and 137 transcription factors. Because this data set is incomplete and there are no negative controls, the appropriate measures to compare the performance of the algorithms is recall and precision, see [Additional file 1] for the procedural details.

The results for two data sets and three algorithms is presented in Table 2. Our algorithm, which takes advantage of gene-regulon associations rather than the gene-TF associations, outperforms the other two algorithms. The improvement was at least in part due to the fact that the regulatory outcome is a result of the activity of transcription factors and not necessarily of their levels, and the knowledge of transcript levels of the regulators' genes is not sufficient to predict the interactions. Both the GGM and the relevance networks construct the relationship between the gene target expression levels and the expression levels of genes encoding transcription factors. Such models are confined to the cases when regulators are members of their own regulons. However, in many cases, if not most, transcriptional regulation of targets is not accompanied by changes in the levels of the regulators' transcripts, and even when it is, the changes don't have to be correlated with the transcription of the targets. Instead, such regulation can be captured when estimating the activity of the transcription factors, which is the basis of the method presented in the current study.

**Table 2:** Comparison of recall(Precision) (%), rounded to the closest integer, for the model selection algorithm, relevance network and graphical gaussian model on two large-scale microarrays data sets.

	Methods		
Data sets	Model selection algorithm (this paper)	Relevance Network	GGM

Our data set	44 (43)	8 (6)	3(3)

Data set in [17]	62 (64)	20 (16)	3(2)

The second reason for outperforming the relevance network algorithm is the capacity of our method to capture condition-specific activity and the co-variance of the gene expression profiles across different conditions. On the other hand, the relevance network algorithm treats gene expression levels across different conditions as ensembles of single random variables to estimate the probability distribution for each gene, and therefore it can not account for condition-specific activity of the genes when the sample size is not large enough. See [Additional file 1] for more information on the comparison between the algorithms.

### 2.4 Network reconstruction

We applied the proposed method to a large-scale gene expression data set to evaluate its capacity to recover known regulatory interactions and predict new ones. We ignored any TF assignments with a very small number (< 5) of known interactions. We assigned to each gene a regulator based on the maximum probability of association calculated using the proposed algorithm presented in the methods section. When we limited the number of associations for each gene to one, the algorithm was able to recover the correct association for 86% of the genes with known interaction, i.e., 1044 genes out of 1210 genes were associated with their known regulators. When two regulators were assigned to genes, an additional 542 known interactions were recovered, which included known interactions for 26 genes that were not in the set of 1044. That increased the percentage of genes with correct associations to 88%, and this implies that for 74% of genes, i.e. for 516 out of 696 genes having two or more regulators, both predicted regulators were correct.

In addition to recovering known interactions, the algorithm discovered new, un-annotated interactions. Some of the discovered interactions could be independently confirmed. For example, the algorithm predicted two targets of ArgR, *hisJ *and *artJ*, which were not among known interactions in the RegulonDB database, but have been recently reported in the literature [31]. For this particular TF, 21 out of 27 known interactions in regulonDB were recovered.

We specifically focused on the structure of the Lrp regulon. Lrp is a global transcription factor and a mediator of leucine response. It is believed that Lrp controls the expression of hundreds of genes directly or indirectly[32], although only 55 known Lrp targets were annotated in the RegulonDB at the time of this study. Overall, 85 genes were predicted to be Lrp targets. By using transcriptional data obtained on an Lrp knock-out mutant [32], we confirmed that 52 genes were differentially expressed in the knock-out strain. Using chromatin immuno-precipitation(IP), we found that Lrp binds to the upstream regions of at least 45 out of the 52 differentially expressed genes, including several new predicted targets such as *ompT*, *dppA*, *eco*, *pntA*, *pntB*, *csiE*, *sdaB sdaC*, *yhjE *and *ygdH*, most of which were also verified using a qPCR experiment (Table 3). Thus, the algorithm discovered 10 *new *targets of the transcription factor Lrp that could be confirmed by a biological experiment. In addition, limited evidence in the literature suggests that *sdaBC *and *pntAB *are also likely Lrp targets [33,34]. *pntAB *is also among predicted targets for lrp in [17]. Our results also indicated that Lrp controls expression of the *leuABC *operon. Although according to RegulonDB there is no interaction between Lrp and the *leuABC *operon, it has been previously reported that Lrp does regulate this operon [33]. Overall, this rate of discovery of true Lrp targets is higher than any previously reported discovery rate of confirmed targets for any regulator by a discovery algorithm.

**Table 3:** New targets of Lrp which were confirmed using qPCR (the fold enrichment values with '*' are from ChIP-chip)

Gene name	Fold transcript change	Fold IP enrichment	Lrp Activity	Function
ompT	8.5	2.8	Positive	DLP12 prophage; outer membrane protease VII

eco	1.8	2.3	Negative	ecotin, serine protease inhibitor

dppA	1.6	3.4	Positive	dipeptide transporter

pntA	1.6	2.8	Positive	pyridine nucleotide transhydrogenase, alpha subunit

artP	1.7	4	Negative	arginine periplasmic transport system

sdaC	-	1.9	Positive	predicted serine transporter

yhjE	1.5	6.9	Negative	putative transporter

csiE	-	2.1*	Negative	stationary phase inducible protein

ygdH	-	2*	Positive	unknown

### 2.5 Activity of regulators

The principal eigenvector of the covariance matrix computed from the set of genes controlled by each regulator was assumed to be a good and biologically sound approximation of the activity profiles of the regulators. We proceeded to evaluate this assumption by examining the estimated activity levels of transcription factors in individual conditions and by comparing the eigenvector-derived profiles with the activity profiles calculated by the Network Component Analysis, a state of the art connectivity matrix decomposition technique proposed in [27]. (Note, the activity levels of TFs are the relative activities of TFs in each condition with respect to a reference sample.)

**Figure 1:**
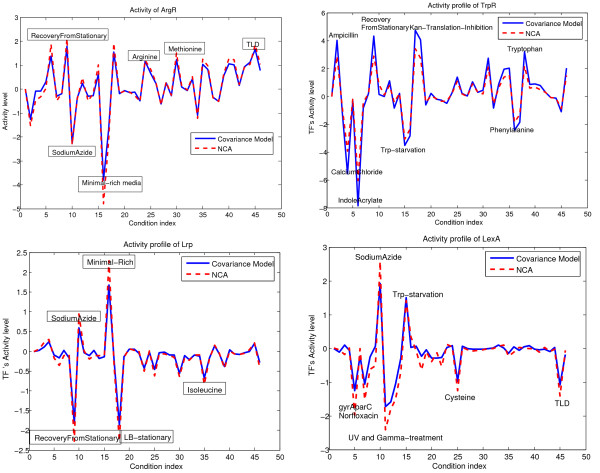
**Activity profile of ArgR, TrpR, Lrp and LexA**. Several conditions in our data set were expected to elicit transcriptional responses mediated by the activity of known regulators. We found that in all conditions with well-studied and understood transcriptional responses, the identity of the most active TF matched our expectations. For example, in an experiment which was conducted to measure transcriptional response to addition of the amino acid arginine, transcription factor ArgR appeared to be the most active TF. Similarly, TrpR was the most active TF in the condition when tryptophan was added to the medium, and LexA was the most active TF under conditions of UV and Gamma treatment.

We determined that the eigenvector-derived profiles of regulators' activity fully recapitulate NCA profiles. Figure 1 illustrates this point on several characteristic profiles (some conditions in which the activity level of the TF was significant are indicated).

Several conditions in our data set were expected to elicit transcriptional responses mediated by the activities of known regulators. Indeed, we found that in all conditions with well-studied and understood transcriptional responses, the identity of the most active TF matched our expectations. For example, in the experiment to measure transcriptional response to the addition of the amino acid arginine, transcription factor ArgR appeared to be the most active TF. Similarly, TrpR was the most active TF in the condition when tryptophan was added to the medium, and LexA was the most active TF under conditions of UV and Gamma treatment Fig. 1.

**Figure 2:**
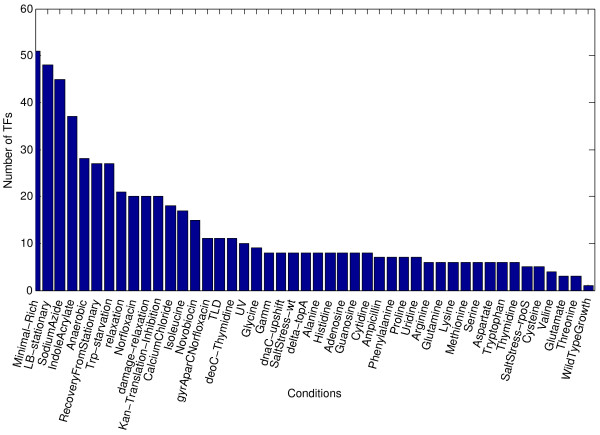
**A number of active regulators varies across conditions**. The number of transcription factors active in minimal growth medium as compared to rich medium was the highest, followed by the transition from exponential to stationary phase of growth, during which the cells are known to undergo massive regulatory re-programming, and then sodium azide treatment, which results, among other things, in an interruption of the electron flow chain. Among the amino acid effects, addition of isoleucine appeared to stimulate the highest number of TFs, whereas addition of threonine or glutamate appeared to have no or very little effect on the regulators. The smallest number of differentially active transcription factors was observed in the comparison of chemostat cultures grown at different dilution rates ("WildTypeGrowth").

In almost all conditions, we were able to identify more than one active transcription factor. When we considered a transcription factor to be active at a significance level of 5% (the z-score corresponding to each activity level was calculated from the background distribution estimated from all activity levels), on average 13 TFs were active per condition in our data set (11 – in the Affymetrix data set). The distribution of the number of active TFs across the conditions is shown in Fig. 2. The number of transcription factors active in a minimal growth medium as compared to rich medium was the highest, followed by the transition from exponential to stationary phase of growth, during which the cells are known to undergo massive regulatory re-programming, followed by sodium azide treatment, which results among other things in interrupting the electron flow chain. Among the amino acid effects, addition of isoleucine appeared to stimulate the highest number of TFs, whereas addition of threonine or glutamate appeared to have no or very little effect on the regulators. The smallest number of differentially active transcription factors was observed in the comparison of chemostat cultures grown at different dilution rates ("WildTypeGrowth").

**Figure 3:**
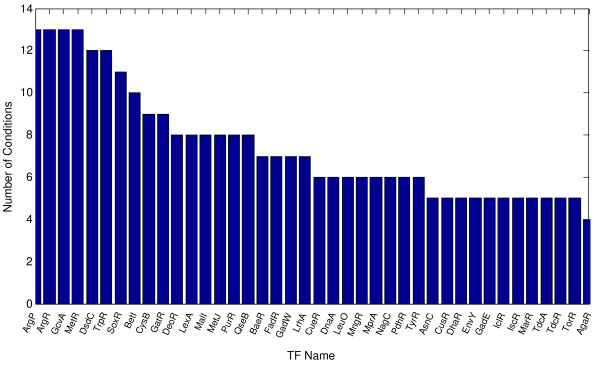
**Frequency of condition-specific activity for top regulators**. Many TFs were likely to be mediating transcriptional responses in multiple conditions. Given that the set of conditions in our study was enriched by those in which metabolism of various amino acids or nucleotides was directly or indirectly perturbed and by conditions causing DNA damage, it was not surprising that the list of most frequently active regulators included ArgR, GcvA, CysB, MetR/MetJ, DeoR, PurR, LexA.

Not only did we find that more than one TF appeared to be active in any given condition, but also that many TFs were likely to be mediating transcriptional responses in multiple conditions (Fig. 3). Given that the conditions in our study were enriched by perturbations of amino acid, nucleotide and DNA metabolism, it was not surprising that the list of most frequently active regulators included ArgR, GcvA, CysB, MetR/MetJ, DeoR, PurR, LexA. What we found surprising was that the transcription factor TrpR, the main transcriptional regulator of genes involved in tryptophan biosynthesis, appeared to be the most responsive regulator in a sense that it was not only among the top responsive TFs to different conditions, but also its activity level was higher than those of other TFs (the method allowed comparison of the activity levels of various regulators across a uniform scale; see the scale on Y axis in Fig. 1). Tryptophan is the scarcest amino acid in the cell; it is plausible that many perturbations, including those that don't affect tryptophan metabolism directly, may result in biologically significant fluctuations in the size of the amino acid pool. Despite the absence of any apparent bias in the Affymetrix data set [17], the frequency of OxyR activity (OxyR activates hydrogen peroxide induced genes) dwarfed the frequencies of all other factors: OxyR was active in almost thrice as many conditions as the next most frequently active regulator.

The correlation analysis of TFs activity profiles revealed that the activity profiles of almost half of the transcription factors considered in this study are correlated with one another. Some global regulators such as CRP, IHF, FNR were correlated with more than 10 TFs. However, some local regulators, such as OmpR, PhoP, and EnvY, also showed a high degree of correlation with other TFs. Figure S1 [see Additional file 2] shows the network view of correlation between transcription factors. The existence of the edge between two TFs indicates that the correlation between their activity profiles is above a threshold value of 0.70. Such similarities may indicate a certain degree of regulatory redundancy, i.e. different regulators controlling subsets of overlapping genes. Indeed, when we examined to what extent the correlations between the profiles are indicative of TFs regulating common genes, we observed that transcription factor pairs with high correlation regulate common genes with higher probability than TF pairs with low correlations. 55% of TF pairs with correlation above 0.70 appeared to have common targets, compared to 20% of TF pairs with correlation less than 0.70.

However several transcription factors, including LexA, GcvA, SoxR, DsdC and FadR, did not show high degree of correlation with other TFs, even though they were relatively responsive in a high number of conditions in the study.

### 2.6 Network refinement

Based on the two expression data sets, covering regulatory states for almost all genes in the genome, each gene (operon) was assigned a transcription factor(s) that was likely to control the expression of that gene. To this end, the present algorithm was applied to both data sets. For each data set, every gene was assigned three top-ranking regulators with the highest probabilities of association, calculated using equation (3). If a gene had the same regulator(s) predicted from both sets, the regulatory association between the gene and regulator(s) was deemed true and cataloged for the purpose of network refinement. This resulted in a list of 1719 genes associated with at least one regulator [see Additional file 3]. 779 genes out of 1719 had no previously characterized regulatory interactions. The existence of consensus regulators (predicted using two completely independent data sets) for this number of genes is statistically significant. Only 167 genes would have been expected to have a consensus regulator if the assignments in one of the sets had been done at random.

We incorporated the operon information to further refine the set of regulatory interactions. The consensus regulator for an operon was chosen as a common regulator of genes in the same operon, as predicted on the basis of both data sets. This resulted in a list of potential regulator(s) for each operon [see Additional file 4], with many already confirmed or highly plausible regulatory interactions. For example, *dinI *and *dinP *are known targets of LexA, but not among the known interactions set used in this study. Also, yafNOP, *yebB*, *yebG*, *yigN*, and *yjjB-dnaTC-yjjA *were predicted to be LexA targets. *yafNOP *is a neighboring operon of *dinB *with a weak but significant score for a LexA binding site in its promoter region. Regulatory regions of *yebG*, *yigN *and *yebB *contain high scoring LexA binding sites. The possibility that *dnaT *and *dnaC*, two genes involved in DNA replication, are under LexA control is intriguing, and warrants further experimentation.

Overall, even though the two data compendia appeared to be substantially different as far as dominant activity profiles are concerned, transcriptional profiles of as many as 1407 genes and 773 multigene operons could be explained at least in part by the activity of the same regulator(s) in both data sets. This result implies that, provided a sufficiently diverse collection of experimental conditions, the method will converge on true transcriptional regulators of any given gene in a genome, including regulators themselves [see Additional files 1 and 5].

## 3 Conclusion

Genome-wide transcriptional data allow for systematic analysis of regulatory patterns of gene expression. These patterns are at least in part determined by interactions between transcription factors and their cognate target genes. Accurate prediction of such interactions, which is essential for understanding phenotypic outcomes of genetic and environmental perturbations, depends on the quality of models capturing essential regulatory features and on their underlying assumptions. One such feature is that the transcriptional activity of co-regulated genes should sufficiently absorb in itself the activity of their common regulator. Moreover, the information about transcriptional activity of the known co-regulated genes (a core regulon) should also be sufficient for discovering new target genes, whose transcriptional activity significantly co-vary with the activity of the core regulon members.

We introduced an effective approach to predict interactions between regulators and genes through a simple model selection algorithm. The algorithm takes advantage of both gene expression data and the knowledge of known gene-TF associations to simultaneously discover new interactions between regulators and genes and to estimate the activity profiles of the regulators. The proposed approach, unlike other methods, associates the expression of genes in a regulon with the activity of transcription factors rather than with the expression levels of genes encoding for the transcription factors. We demonstrated that incorporation of information about the activity profiles of transcription factors allows for a reliable identification of many known as well as previously uncharacterized regulatory interactions, which could not be achieved by methods solely relying on gene expression associations. We should mention that the power of a regulon-based association framework presented in this paper and of the supervised inference method [35] relies on the prior information about known interactions between genes and transcription factors: without such information these supervised algorithms are not applicable. However, with the help of ChIP-chip and ChIP-sequencing technology, it should be possible to obtain sufficient amount of data to seed a *de novo *interactivity matrix, which can be used in combination with gene expression data to construct a reliable gene regulatory network by means of these supervised learning methods.

## 4 Methods

We consider the gene regulation process as an input-output model where the transcription rate (output product) is controlled through the activity level of the group of specialized transcription factors. We assume, given the activity of regulators, that this process is approximately linear, although the dynamic behavior of this biological process can be much more complex. In the linear model

(1)

**e **represents a vector of gene expression measurements across *m *conditions, **S **is a *m *× *k *matrix of the *k *regulators' activities and **c **is a sparse vector that represents the interaction between the gene and regulators. We assume **S **and **c **are both unknown and *ϵ *is an *m *elements vector of random noise with zero mean and covariance matrix of *σ*^2^**I**.

The fact that the expression level of genes is controlled by only a few regulatory nodes makes the resulting network, and therefore **c**, sparse. Furthermore, the direct measurement of the regulators' activity is a challenging and expensive, if not an impossible, task. Therefore, one does not have access to **S**, the activity profiles of regulators. The objective is to estimate the activity profiles of the regulators that control transcription (initiation) of groups of genes. At the same time, we also wish to provide for each gene the set of regulators that can explain the observed gene expression data.

Here, we present an efficient algorithm, which takes advantage of known interaction among genes and regulators. Let Ω_*f *_be a set consisting of the expression vectors of genes known to be controlled by a single regulator *f*. Then,

(2)

where **s**_*f*_, the activity profile of the regulator *f*, is unknown and *c*_*f *_is the interaction coefficient, which is assumed to be a random variable with distribution (*υ*, *γ*^2^). The likelihood function for **s**_*f *_[36] is,

(3)

where



Among the set of all regulators, the correct regulator, **s**_*f*_, maximizes *p*(**e**_*g*_|**s**_*f*_). On the other hand, to compute *p*(**e**_*g*_|**s**_*f*_) the regulatory signals need not be known explicitly and the knowledge of the sample mean (*μ*_*f*_) and the covariance matrix (Σ_*f*_) corresponding to each set Ω_*f *_is sufficient for model selection. Therefore, one only needs to estimate these sample mean and covariance matrices from the data. The covariance matrix Σ_*f*_, which is the covariance matrix of gene expression data in Ω_*f*_, can be estimated using sample covariance matrix. Assume **E**_*f *_to be the expression measurements of genes in Ω_*f*_, then one can estimate the weighted covariance matrix corresponding to regulator *f *by,

(4)

where *N*_*f *_is the number of samples in Ω_*f*_, *μ*_*i *_and *μ*_*j *_are *i*th and *j*th entries of sample mean, *μ*_*f *_and *w*_*k*_'s are weights which sum to one.

Since we considered Ω_*f*_'s as sets of genes controlled only by one regulator, if **s**_*f *_represents the true model, then the covariance matrix Σ_*f *_can be represented by its first rank approximation using eigenvalue decomposition. Therefore, ignoring the noise terms, the inverse of the covariance matrix can be efficiently computed through its first rank approximation, which not only speeds up the algorithm but also reduces the effects of the noise.

(5)

where *λ*_1 _and **u**_1 _are respectively the principal eigenvalue and the principal eigenvector of the covariance matrix. Given data sets of known TF-gene interactions one can form the sets of Ω_*f*_'s and use equations 4,5 and 3, respectively, to compute *p*(**e**_*g*_|**s**_*f*_) for all TFs and assign to each gene *g *the regulator *f *that provides the maximum value.

The knowledge of regulator-gene interactions represents a low-resolution view of molecular interactions inside a cell. It does not provide any details about how and when these interactions occur. Therefore, as a complement to this information, in some biological studies, the question might be which regulators and how they respond under different environmental or genetic perturbations. One way to tackle this problem is to study the activity levels of different transcription factors across different conditions. The principal eigenvector (eigenvector corresponding to the largest eigenvalue) of the sample covariance matrix (Σ_*f*_) can be viewed as the activity profile of a regulator *f*. Notice that the activity profiles of regulators are principal eigenvectors of different covariance matrices, which necessarily need not be, and, indeed they are not, orthogonal to each other. Therefore, the estimated activity profiles are different from those estimated by algorithms such as Principal component analysis (PCA) [22] or singular value decomposition [23,24] and independent component analysis (ICA)[26] which decompose the original data into a few regulatory signals that are orthogonal or independent.

## Authors' contributions

HZ conceived the method, designed the study, performed the analysis and wrote the manuscript. DS carried out the DNA microarray experiments for amino acid data set. PS obtained the chromatin-IP data for Lrp. MK participated in the design of the study and wrote the manuscript. AK coordinated the study, analyzed microarray data, and wrote the manuscript. All authors read and approved the final manuscript.

## Supplementary Material

Additional file 1 **Supplementary Information**. Additional information about performance evaluation, algorithms comparison, and transcription factor sub-networks. Click here for file

Additional file 2 **Supplementary Figure 1(Figure S1)**. Network of transcription factors with correlated profiles. The existence of an edge between two TFs indicates that the correlation between their activity profiles is above a threshold value of 0.70. Such similarities may indicate a certain degree of regulatory redundancy, i.e. different regulators controlling subsets of overlapping genes. Indeed, when we examined to what extent the correlations between the profiles are indicative of TFs regulating common genes, we observed that transcription factor pairs with high correlation regulate common genes with higher probability than TF pairs with low correlations. 55% of TF pairs with correlation above 0.70 appeared to have common targets, compared to 20% of TF pairs with correlation less than 0.70. Click here for file

Additional file 3 **TF-Gene Interactions**. A set of genes with common regulators predicted from both data sets. Click here for file

Additional file 4 **TF-Operon Refined Interactions**. A refined catalog of transcriptional interactions. Click here for file

Additional file 5 **Supplementary Figure 2(Figure S2)**. Regulatory network of transcription factors. The consensus regulatory interactions predicted using both data sets. This subnetwork comprises of 101 transcription factors (nodes) with 118 predicted interactions (edges) among them. All interactions are directed from a TF-regulator toward a TF-target. 76 (66%) predicted interactions (red edges) were previously known and include 36 known auto-regulators. The remaining 42 predicted interactions (blue edges) are new. In addition, 13 regulators identified as targets did not have any previously identified regulators. Click here for file
