# Supplementary material for: Reconstruction of Escherichia coli transcriptional regulatory networks via regulon-based associations
Source: BMC Syst Biol. 2009 Apr 14;3:39. doi: 10.1186/1752-0509-3-39 (PMC2689187; doi:10.1186/1752-0509-3-39)
Supplement: Additional file 1 — Supplementary Information. Additional information about performance evaluation, algorithms comparison, and transcription factor sub-networks. [file 1752-0509-3-39-S1.pdf]

Reconstruction of Escherichia coli transcriptional regulatory networks through regulon-based associations: Supplementary file

1 Performance evaluation

For the relevance network and GGM, we define recall as a fraction of 1210 genes (genes with known interactions) successfully associated with their cognate regulators. The precision is defined as a fraction of predicted interactions which are correct. However, because our algorithm uses the set of known interactions as a training data to estimate transcription factor activity profiles and covariance matrices, we split this data into a training and a test set in order to properly measure the performance of our algorithm. We only used the training part to estimate the covariance matrices inorder to predict the transcription factor-gene interactions. The test data was only used to calculate the recall and precision. We repeated this process 100 times and in each run the test set included 100 genes randomly selected from 1210 genes with known interactions and the training set contains the remaining 1110 genes. The final recall and precision were calculated by averaging over 100 recalls and precisions.

2 Comparison between algorithms

The relevance network performed better than the GGM and it worked better for the larger data set, the Affymetrix dataset. This improvement was likely due to a higher number of experimental conditions(arrays) in the second data set, which in turn resulted in a more accurate calculation of mutual information. The performance of the GGM over these two sets was very poor, which further confirms that GGMs are not suitable when dealing with large-scale gene networks.

It is worth mentioning that our algorithm is also faster than the relevance network and GGM. The expensive part of our algorithm is eigenvalue decomposition of covariance matrices, which has complexity of $O(m^3)$, where m is the number of conditions, which is much smaller than the number of genes. On the other hand, the relevance network involves estimation of marginal probability distributions of genes and joint probability distributions of all gene pairs to calculate pair-wise mutual information. This process is time-consuming when the number of genes is large. The expensive part of the GGM is the calculation of a partial correlation matrix, which is based on calculating the inverse or pseudo-inverse of a large matrix ($n \times n$), where n is the number of genes. The computational complexity of pseudo-inverse is $O(n^3)$, which can be very time-consuming when n is large, such as in large-scale gene networks.

The approximation of TF activities by principal eigenvectors of the covariance matrices is in agreement with the activity profile computed by NCA algorithm (Fig. 1). However, the former is much faster due to the fact that NCA needs to find activity profiles and to quantify connectivity matrix by iteratively solving many least square problems. If K iterations are required for convergence of the algorithm, then $K(n + m)$ least square problems (each $O(r^3)$, where r is the number of transcription factor)have to be solved, where n is the number of genes and m is the number of conditions in the gene expression data set. Even considering the sparsity of the connectivity matrix the complexity is much higher than eigenvalue decomposition of L small covariance matrices, where L is the number of covariance matrices corresponding to L transcription factors. In addition to the fact that the proposed algorithm is computationally simpler than NCA when estimating transcription factor activity profiles, it can also discover new, perviously un-characterized associations between genes and transcription factors.

3 Sub-network of regulators

We searched for an intra-regulatory network as a special subnetwork of the complete gene regulatory network. This network contains regulatory interactions between transcription factors. Figure S2[See Additional file 5] depicts such a network constructed from the list of consensus regulatory interactions predicted using both data sets. This subnetwork comprises of 101 transcription factors (nodes) with 118 predicted interactions (edges) among them. All interactions are directed from a TF-regulator toward a TF-target. 76 (66%) predicted interactions (red edges) were previously known and include 36 known auto-regulators. The remaining 42 predicted interactions (blue edges) are new. In addition, 13 regulators identified as targets

did not have any previously identified regulators. It remains to be seen whether the described and potential future connectivity refinements affect global or local topological properties of the network.
