# Supplementary figures and images for: Reconstruction of Escherichia coli transcriptional regulatory networks via regulon-based associations
Source: BMC Syst Biol. 2009 Apr 14;3:39. doi: 10.1186/1752-0509-3-39 (PMC2689187; doi:10.1186/1752-0509-3-39)

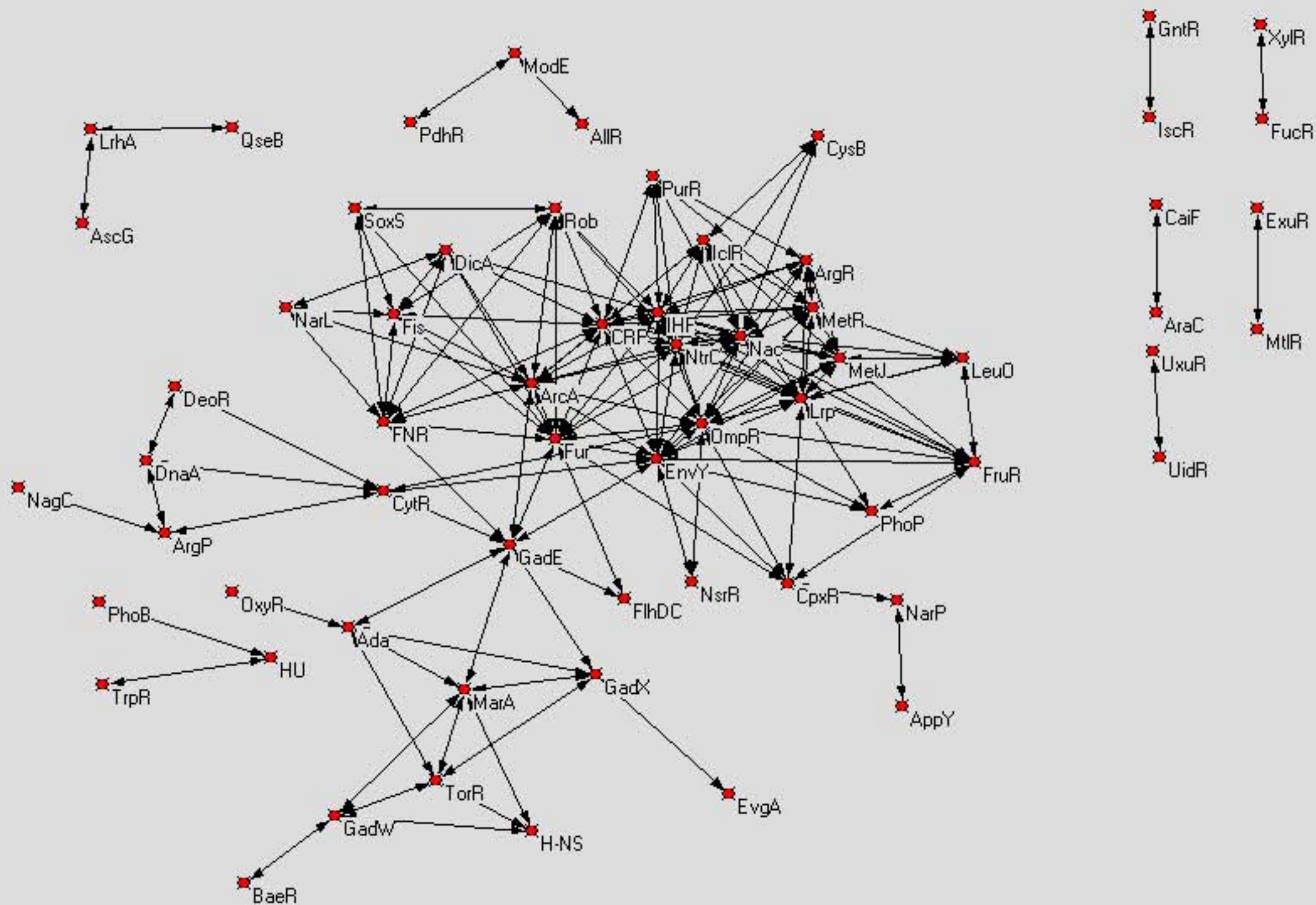

Supplement: Additional file 2 — Supplementary Figure 1(Figure S1). Network of transcription factors with correlated profiles. The existence of an edge between two TFs indicates that the correlation between their activity profiles is above a threshold value of 0.70. Such similarities may indicate a certain degree of regulatory redundancy, i.e. different regulators controlling subsets of overlapping genes. Indeed, when we examined to what extent the correlations between the profiles are indicative of TFs regulating common genes, we observed that transcription factor pairs with high correlation regulate common genes with higher probability than TF pairs with low correlations. 55% of TF pairs with correlation above 0.70 appeared to have common targets, compared to 20% of TF pairs with correlation less than 0.70. [file 1752-0509-3-39-S2.pdf]

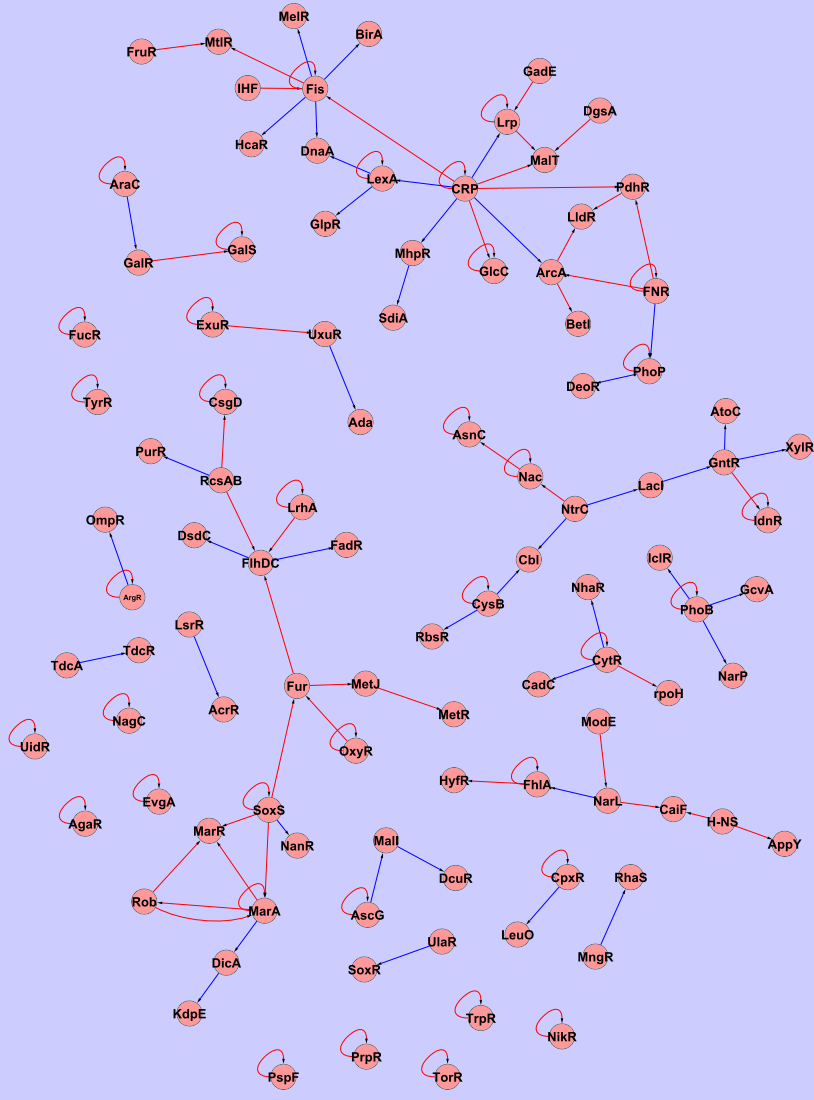

Supplement: Additional file 5 — Supplementary Figure 2(Figure S2). Regulatory network of transcription factors. The consensus regulatory interactions predicted using both data sets. This subnetwork comprises of 101 transcription factors (nodes) with 118 predicted interactions (edges) among them. All interactions are directed from a TF-regulator toward a TF-target. 76 (66%) predicted interactions (red edges) were previously known and include 36 known auto-regulators. The remaining 42 predicted interactions (blue edges) are new. In addition, 13 regulators identified as targets did not have any previously identified regulators. [file 1752-0509-3-39-S5.pdf]
