# Supplementary material for: Reconstruction of Escherichia coli transcriptional regulatory networks via regulon-based associations
Source: BMC Syst Biol. 2009 Apr 14;3:39. doi: 10.1186/1752-0509-3-39 (PMC2689187; doi:10.1186/1752-0509-3-39)
Supplement: Additional file 3 — TF-Gene Interactions. A set of genes with common regulators predicted from both data sets. [file 1752-0509-3-39-S3.pdf]

			Data Set 1			Data Set 2			Common	
Gene Name	Gene BID	Regulator1	Regulator2	Regulator3	Regulator1	Regulator2	Regulator3	Regulator1	Regulator2	Regulator3
yjbN	b4049	GadX	rpoH	H-NS	rpoH	PhoP	CpxR	rpoH		
ompF	b0929	OmpR	EnvY	Lrp	Lrp	CpxR	OmpR	OmpR	Lrp	
yjbJ	b4045	Fur	H-NS	IHF	IHF	CRP	ArcA	IHF		
dinF	b4044	LexA	Fur	CpxR	LexA	CRP	ArcA	LexA		
lexA	b4043	LexA	Lrp	CRP	LexA	CRP	ArcA	LexA	CRP	
ycbK	b0926	ArcA	IHF	CRP	H-NS	CRP	IHF	IHF	CRP	
ubiA	b4040	NarL	IHF	ArcA	NarL	CpxR	FNR	NarL		
mukE	b0923	SoxS	H-NS	MarA	H-NS	CpxR	Fis	H-NS		
mukF	b0922	H-NS	CpxR	PhoB	H-NS	SoxS	CpxR	H-NS	CpxR	
smtA	b0921	H-NS	CpxR	PhoB	H-NS	CpxR	Fis	H-NS	CpxR	
ubiC	b4039	NarL	IHF	ArcA	NarL	Fur	GadE	NarL		
malM	b4037	MalT	CRP	Fis	MalT	CRP	FNR	MalT	CRP	
lamB	b4036	MalT	CRP	IHF	MalT	CRP	FNR	MalT	CRP	
malK	b4035	CRP	MalT	FNR	MalT	CRP	FNR	CRP	MalT	FNR
malE	b4034	MalT	CRP	Fis	MalT	CRP	FNR	MalT	CRP	
malF	b4033	H-NS	CRP	Fis	MalT	CRP	FNR	CRP		
malG	b4032	CRP	GntR	Fis	MalT	CRP	FNR	CRP		
rpsA	b0911	FNR	ArcA	Fis	FNR	Fis	CRP	FNR	Fis	
cmk	b0910	FNR	Fis	LexA	Fis	FNR	CRP	FNR	Fis	
pgi	b4025	CRP	Fis	FNR	FNR	CRP	IHF	CRP	FNR	
aroA	b0908	Lrp	CRP	MetJ	Lrp	CRP	IHF	Lrp	CRP	
lysC	b4024	CRP	FNR	Lrp	CRP	ArgR	Lrp	CRP	Lrp	
serC	b0907	Lrp	CRP	IHF	Lrp	CRP	IHF	Lrp	CRP	IHF
ycaO	b0905	CRP	IHF	Fis	Fis	rpoH	PurR	Fis		
focA	b0904	NarL	ArcA	IHF	NarL	FNR	ArcA	NarL	ArcA	
pflB	b0903	NarL	ArcA	IHF	NarL	ArcA	FNR	NarL	ArcA	
metH	b4019	MetR	Fur	Lrp	MetR	TyrR	TrpR	MetR		
aceK	b4016	FruR	IclR	IHF	FruR	IHF	ArcA	FruR	IHF	
aceA	b4015	FruR	IclR	ArcA	FruR	IclR	ArcA	FruR	IclR	ArcA
aceB	b4014	IclR	FruR	ArcA	FruR	IclR	ArcA	IclR	FruR	ArcA
metA	b4013	MetJ	MetR	rpoH	MetR	MetJ	rpoH	MetJ	MetR	rpoH
purH	b4006	PhoP	PurR	FNR	PhoP	PurR	Lrp	PhoP	PurR	
purD	b4005	PhoP	NikR	GntR	PhoP	PurR	TyrR	PhoP		
hydG	b4004	Cbl	AppY	CaiF	MhpR	AgaR	Cbl	Cbl		
hupA	b4000	Fis	CRP	CpxR	Fis	CRP	IHF	Fis	CRP	
mtlA	b3599	FruR	Fis	CRP	FruR	Fis	PhoB	FruR	Fis	
aldB	b3588	Fis	CRP	IHF	Fis	CRP	NtrC	Fis	CRP	
upp	b2498	FNR	IHF	CRP	FNR	CRP	IHF	FNR	IHF	CRP
uraA	b2497	FNR	PurR	IHF	FNR	PurR	IHF	FNR	PurR	IHF
b2494	b2494	CpxR	PhoB	FNR	CpxR	PhoP	CysB	CpxR		
yiaK	b3575	GalR	GalS	YiaJ	NarP	YiaJ	NtrC	YiaJ		
yiaI	b3573	IclR	NarL	ExuR	NarL	NarP	FNR	NarL		
avtA	b3572	FlhDC	TrpR	IHF	IHF	Lrp	FNR	IHF		
hyfG	b2487	FhlA	HyfR	AscG	AgaR	HyfR	EvgA	HyfR		
bax	b3570	Fis	FNR	H-NS	Fis	CRP	FNR	Fis	FNR	
hyfF	b2486	FhlA	HyfR	NarL	AgaR	HyfR	MarR	HyfR		
hyfB	b2482	FhlA	HyfR	FucR	HyfR	FadR	UxuR	HyfR		
bcp	b2480	CRP	CpxR	Fur	CpxR	CRP	Lrp	CRP	CpxR	
b1391	b1391	PaaX	FlhDC	ModE	NagC	MarA	FlhDC	FlhDC		
xylR	b3569	CaiF	GntR	AtoC	YiaJ	GntR	FlhDC	GntR		
xylF	b3566	XylR	Fis	CRP	Fis	FruR	CRP	Fis	CRP	
xylA	b3565	XylR	GntR	Fis	FruR	Fis	NtrC	Fis		
gcvR	b2479	CpxR	CRP	Fis	CRP	Lrp	FNR	CRP		
glyQ	b3560	rpoH	CpxR	FNR	Fis	rpoH	FNR	rpoH	FNR	
purC	b2476	PurR	FNR	CRP	PurR	Lrp	CRP	PurR	CRP	
dapE	b2472	rpoH	PhoB	NhaR	PhoB	rpoH	FlhDC	rpoH	PhoB	
acrD	b2470	EvgA	BaeR	GntR	BaeR	RcsAB	PhoB	BaeR		

tynA	b1386	CusR	NagC	RcsAB	RcsAB	rpoH	FlhDC	RcsAB		
feaB	b1385	GntR	ModE	FlhDC	FlhDC	Rob	rpoH	FlhDC		
feaR	b1384	CRP	Fis	H-NS	Fis	CRP	IHF	CRP	Fis	
ydbL	b1383	PaaX	IscR	NarL	NarL	PhoB	FlhDC	NarL		
ldhA	b1380	rpoH	Fis	IHF	rpoH	IHF	CRP	rpoH	IHF	
glyS	b3559	rpoH	CpxR	FNR	Fis	CpxR	SoxS	CpxR		
cspA	b3556	Fis	CRP	FNR	CRP	H-NS	FNR	CRP	FNR	
yiaG	b3555	H-NS	IHF	Fis	CRP	Lrp	IHF	IHF		
yiaD	b3552	Fis	CRP	FruR	CRP	IHF	ArcA	CRP		
bisC	b3551	DicA	YiaJ	UlaR	PspF	UlaR	BaeR	UlaR		
tktB	b2465	MarA	SoxS	H-NS	SoxS	Lrp	IHF	SoxS		
talA	b2464	MarA	SoxS	H-NS	Lrp	SoxS	Fis	SoxS		
b2463	b2463	IHF	ArcA	CRP	FlhDC	IHF	CRP	IHF	CRP	
hslJ	b1379	CysB	RcsAB	rpoH	CysB	IHF	CRP	CysB		
b2460	b2460	HU	MetJ	NagC	NagC	GntR	AgaR	NagC		
b1374	b1374	Fur	CRP	H-NS	FNR	H-NS	CRP	CRP	H-NS	
yagU	b0287	Fis	IHF	CRP	CRP	FNR	IHF	IHF	CRP	
trs5_5	b1370	CytR	NarL	LexA	ArgR	FlhDC	LexA	LexA		
yagR	b0284	CusR	NagC	NtrC	ArgR	FlhDC	NtrC	NtrC		
yagQ	b0283	MprA	Cbl	CaiF	MprA	PhoB	ArgR	MprA		
yagP	b0282	MetR	Rob	CsgD	CsgD	LexA	PhoB	CsgD		
yagN	b0280	SoxS	MarA	CRP	IHF	CRP	ArcA	CRP		
yhjY	b3548	EvgA	GadX	PhoB	NtrC	PhoB	SoxS	PhoB		
yhjW	b3546	CpxR	H-NS	ModE	LexA	FruR	CpxR	CpxR		
dppA	b3544	IHF	FNR	Lrp	IHF	FNR	CRP	IHF	FNR	
dppB	b3543	IHF	FNR	CpxR	IHF	FNR	CRP	IHF	FNR	
dppC	b3542	IHF	FNR	FruR	IHF	FNR	Lrp	IHF	FNR	
dppD	b3541	IHF	FNR	CRP	IHF	FNR	CRP	IHF	FNR	CRP
dppF	b3540	IHF	FNR	CRP	IHF	FNR	CRP	IHF	FNR	CRP
eutJ	b2454	AgaR	AllR	GntR	FadR	NagC	GntR	GntR		
b1369	b1369	NikR	FlhDC	ModE	ArgR	FlhDC	LexA	FlhDC		
b1364	b1364	IscR	IHF	CRP	CpxR	CRP	FNR	CRP		
argF	b0273	ArgR	CRP	IHF	ArgR	IHF	FNR	ArgR	IHF	
yhjN	b3532	PhoB	BaeR	NarP	AgaR	PhoB	CsgD	PhoB		
b2447	b2447	AllR	ModE	RcsAB	Nac	RcsAB	ExuR	RcsAB		
ydaT	b1358	NikR	GntR	RcsAB	RcsAB	ArgR	NagC	RcsAB		
ydaS	b1357	NikR	FlhDC	NtrC	FlhDC	rpoH	CpxR	FlhDC		
ydaR	b1356	CRP	H-NS	Fis	CRP	IHF	FNR	CRP		
dctA	b3528	DcuR	ArcA	CRP	ArcA	CRP	DcuR	DcuR	ArcA	CRP
kdgK	b3526	TyrR	PhoB	FruR	FruR	CRP	ArcA	FruR		
yhjH	b3525	FlhDC	CpxR	H-NS	FlhDC	CpxR	H-NS	FlhDC	CpxR	H-NS
yhjE	b3523	Lrp	CRP	H-NS	Lrp	CRP	IHF	Lrp	CRP	
b2439	b2439	NarL	NarP	CpxR	rpoH	ArgR	CpxR	CpxR		
b2430	b2430	CytR	AgaR	CdaR	MarR	PhoB	AgaR	AgaR		
treF	b3519	NtrC	IHF	FNR	Ada	NtrC	SoxS	NtrC		
gadA	b3517	TorR	GadE	GadX	TorR	GadX	GadE	TorR	GadE	GadX
yhiX	b3516	TorR	GadX	GadE	TorR	GadE	GadX	TorR	GadX	GadE
yhiW	b3515	FNR	CRP	H-NS	FNR	H-NS	CRP	FNR	CRP	H-NS
yhiV	b3514	NarP	NarL	FNR	ArcA	MarA	FNR	FNR		
yhiE	b3512	EvgA	GadE	H-NS	EvgA	GadE	H-NS	EvgA	GadE	H-NS
hdeD	b3511	GadE	GadX	TorR	GadE	GadX	H-NS	GadE	GadX	
hdeA	b3510	MarA	TorR	GadE	TorR	MarA	GadE	MarA	TorR	GadE
cysP	b2425	CysB	IHF	CRP	CysB	IHF	H-NS	CysB	IHF	
cysU	b2424	CysB	IHF	FNR	CysB	IHF	H-NS	CysB	IHF	
cysW	b2423	CysB	IHF	ArgR	CysB	IHF	FlhDC	CysB	IHF	
cysA	b2422	CysB	IHF	CRP	CysB	IHF	H-NS	CysB	IHF	
cysM	b2421	CysB	IHF	CRP	CysB	IHF	Lrp	CysB	IHF	
fnr	b1334	CpxR	FNR	Fis	ArcA	FNR	CRP	FNR		
ynaJ	b1332	NarP	FNR	NarL	CRP	ArcA	FNR	FNR		

b1330	b1330	CpxR	Fis	RcsAB	MarA	CpxR	CRP	CpxR		
phoE	b0241	PhoB	H-NS	CRP	PhoB	AllR	ArgR	PhoB		
hdeB	b3509	MarA	TorR	GadX	MarA	TorR	GadE	MarA	TorR	
slp	b3506	MarA	H-NS	FNR	MarA	H-NS	ArcA	MarA	H-NS	
yfeK	b2419	CueR	CpxR	ModE	NarP	NarL	CpxR	CpxR		
arsB	b3502	TdcA	TdcR	RcsAB	YiaJ	RcsAB	AllR	RcsAB		
pdxK	b2418	CaiF	OxyR	SoxS	TyrR	SoxS	Nac	SoxS		
crr	b2417	DgsA	FruR	FadR	FruR	DgsA	CRP	DgsA	FruR	
gor	b3500	OxyR	UlaR	FhlA	OxyR	rpoH	GntR	OxyR		
ptsI	b2416	DgsA	FruR	CRP	FruR	Fis	CRP	FruR	CRP	
ptsH	b2415	DgsA	FruR	CRP	FruR	DgsA	CRP	DgsA	FruR	CRP
cysK	b2414	CysB	IHF	CRP	CysB	IHF	CRP	CysB	IHF	CRP
b1329	b1329	FlhDC	GalR	GalS	FNR	CRP	FlhDC	FlhDC		
lig	b2411	GntR	PhoB	FlhDC	IscR	PhoB	RcsAB	PhoB		
b1327	b1327	Zur	CpxR	LexA	CpxR	NagC	FlhDC	CpxR		
tpx	b1324	Fur	OxyR	FNR	ArcA	FNR	FruR	FNR		
tyrR	b1323	TyrR	LrhA	RcsAB	TyrR	SoxS	PhoP	TyrR		
gpt	b0238	Fis	H-NS	FNR	Fis	CRP	IHF	Fis		
ycjF	b1322	rpoH	H-NS	Fur	rpoH	UxuR	SoxS	rpoH		
pepD	b0237	CsgD	rpoH	FNR	CsgD	FNR	Fis	CsgD	FNR	
ycjX	b1321	rpoH	DnaA	GlpR	rpoH	CRP	IHF	rpoH		
yafP	b0234	LexA	Cbl	Nac	LexA	rpoH	CpxR	LexA		
yafO	b0233	LexA	CpxR	ModE	LexA	CRP	ArcA	LexA		
yafN	b0232	Zur	LexA	Cbl	LexA	CRP	FNR	LexA		
dinP	b0231	LexA	CpxR	H-NS	LexA	CRP	ArcA	LexA		
xapA	b2407	GntR	LsrR	YiaJ	ArgR	RcsAB	GntR	GntR		
gltX	b2400	FNR	CRP	Fis	LexA	GadE	Fis	Fis		
dinJ	b0226	OxyR	rpoH	CpxR	CpxR	CRP	FNR	CpxR		
yafK	b0224	Fur	Fis	IHF	Fis	CRP	IHF	Fis	IHF	
yafH	b0221	FadR	ArcA	CRP	FadR	ArgR	NtrC	FadR		
ykfE	b0220	IHF	FNR	CRP	CRP	Lrp	IHF	IHF	CRP	
ycjM	b1309	AgaR	LexA	HU	Fis	rpoH	LexA	LexA		
pspE	b1308	PspF	IHF	CRP	PspF	IHF	CRP	PspF	IHF	CRP
pspD	b1307	PspF	NsrR	IHF	PspF	ArgR	rpoH	PspF		
pspC	b1306	PspF	RcsAB	IHF	PspF	IHF	rpoH	PspF	IHF	
pspB	b1305	PspF	IHF	CRP	PspF	IHF	ArcA	PspF	IHF	
pspA	b1304	PspF	IHF	CRP	PspF	IHF	NtrC	PspF	IHF	
pspF	b1303	DicA	PspF	LrhA	PspF	NagC	PhoB	PspF		
aldH	b1300	IHF	CRP	FNR	NtrC	PhoB	IHF	IHF		
dnaQ	b0215	PhoB	CpxR	Fur	CysB	CpxR	FlhDC	CpxR		
gloB	b0212	DnaA	SoxS	PhoB	PhoB	Fis	CpxR	PhoB		
dniR	b0211	rpoH	Fis	CRP	CRP	IHF	FNR	CRP		
yafD	b0209	Fur	CpxR	Fis	Fis	CpxR	rpoH	CpxR	Fis	
nac	b1988	Nac	NtrC	CRP	Nac	NtrC	ArcA	Nac	NtrC	
cbl	b1987	CysB	NtrC	CRP	NtrC	CysB	ArcA	CysB	NtrC	
b1983	b1983	H-NS	Fis	CRP	CRP	CpxR	ArcA	CRP		
ycaC	b0897	BaeR	H-NS	FNR	BaeR	H-NS	Lrp	BaeR	H-NS	
dmsC	b0896	ModE	NarL	NarP	NarL	ModE	Fur	ModE	NarL	
dmsA	b0894	ModE	NarL	FNR	ModE	NarL	FNR	ModE	NarL	FNR
serS	b0893	ArcA	CRP	FNR	NarL	FNR	Fis	FNR		
ftsK	b0890	LexA	Nac	TyrR	LexA	NagC	H-NS	LexA		
b1976	b1976	CpxR	FNR	H-NS	CRP	IHF	FNR	FNR		
b1973	b1973	SoxS	Fur	MelR	SoxS	Fur	CRP	SoxS	Fur	
trxB	b0888	SoxS	H-NS	Fis	Fis	FNR	IHF	Fis		
cydD	b0887	NarL	NtrC	PhoB	NarL	PhoB	NtrC	NarL	NtrC	PhoB
b1971	b1971	FlhDC	CRP	Fis	FNR	IHF	CRP	CRP		
cydC	b0886	NarL	Fur	Fis	NarL	PhoB	PhoP	NarL		
infA	b0884	Fis	FNR	CRP	CRP	Fis	FNR	Fis	FNR	CRP
clpA	b0882	CRP	ArcA	IHF	CRP	FNR	ArcA	CRP	ArcA	

yljA	b0881	FruR	IHF	Lrp	IHF	H-NS	CRP	IHF		
cspD	b0880	H-NS	FruR	NtrC	H-NS	CRP	ArcA	H-NS		
yedV	b1968	CRP	CpxR	Fur	FNR	CRP	PhoB	CRP		
yedU	b1967	Fis	FNR	CRP	CRP	IHF	FNR	FNR	CRP	
b1964	b1964	GntR	FlhDC	CpxR	LexA	Fis	CpxR	CpxR		
ybjZ	b0879	rpoH	PhoB	DnaA	rpoH	IHF	CpxR	rpoH		
ybjX	b0877	CRP	FNR	H-NS	FNR	IHF	CRP	CRP	FNR	
dcm	b1961	FlhDC	H-NS	GadE	NarP	FlhDC	LexA	FlhDC		
hcp	b0873	MngR	NarP	AtoC	NarP	NarL	FlhDC	NarP		
poxB	b0871	MarA	SoxS	Fis	SoxS	MarA	IHF	MarA	SoxS	
yedI	b1958	PhoB	NtrC	FruR	TrpR	rpoH	NtrC	NtrC		
b1956	b1956	PhoP	rpoH	CpxR	ArgR	CpxR	NtrC	CpxR		
b1955	b1955	MarA	Lrp	OmpR	NtrC	Lrp	PhoP	Lrp		
b1953	b1953	FruR	OxyR	Fis	Fis	CRP	IHF	Fis		
rcsA	b1951	RcsAB	GadE	FlhDC	RcsAB	GadE	H-NS	RcsAB	GadE	
fliR	b1950	FlhDC	RcsAB	H-NS	FlhDC	H-NS	FNR	FlhDC	H-NS	
ybjP	b0865	IHF	FNR	SoxS	Lrp	IHF	Fis	IHF		
artP	b0864	FlhDC	IHF	CpxR	CRP	ArcA	IHF	IHF		
artJ	b0860	ArgR	CRP	IHF	ArgR	CRP	IHF	ArgR	CRP	IHF
fliQ	b1949	FlhDC	RcsAB	H-NS	FlhDC	H-NS	FNR	FlhDC	H-NS	
fliP	b1948	FlhDC	CRP	FNR	FlhDC	H-NS	FNR	FlhDC	FNR	
fliO	b1947	FlhDC	RcsAB	H-NS	FlhDC	H-NS	CpxR	FlhDC	H-NS	
fliN	b1946	FlhDC	H-NS	CpxR	FlhDC	H-NS	FNR	FlhDC	H-NS	
fliM	b1945	FlhDC	ArcA	CRP	FlhDC	H-NS	FNR	FlhDC		
fliL	b1944	FlhDC	FNR	ArcA	FlhDC	H-NS	FNR	FlhDC	FNR	
ybjF	b0859	Nac	PurR	Fis	ExuR	PhoB	PurR	PurR		
fliK	b1943	FlhDC	FNR	IHF	FlhDC	H-NS	FNR	FlhDC	FNR	
fliJ	b1942	FlhDC	FNR	ArcA	FlhDC	H-NS	FNR	FlhDC	FNR	
potI	b0857	AllR	NtrC	MngR	NtrC	Nac	ArgR	NtrC		
fliI	b1941	FlhDC	FNR	IHF	FlhDC	H-NS	CpxR	FlhDC		
potH	b0856	NtrC	Nac	ArgR	NtrC	TrpR	CpxR	NtrC		
fliH	b1940	FlhDC	H-NS	FNR	FlhDC	H-NS	FNR	FlhDC	H-NS	FNR
potG	b0855	NtrC	ArgR	Nac	NtrC	CpxR	TrpR	NtrC		
potF	b0854	NtrC	H-NS	CRP	NtrC	Lrp	CRP	NtrC	CRP	
ybjN	b0853	SoxS	TyrR	CsgD	SoxS	Fis	CRP	SoxS		
rimK	b0852	SoxS	OxyR	GadX	SoxS	AgaR	ZraR	SoxS		
mdaA	b0851	SoxS	FruR	Lrp	SoxS	Fis	FNR	SoxS		
ybjC	b0850	SoxS	ExuR	ModE	SoxS	Fis	MarA	SoxS		
fliG	b1939	FlhDC	H-NS	FNR	FlhDC	H-NS	FNR	FlhDC	H-NS	FNR
fliF	b1938	FlhDC	H-NS	FNR	FlhDC	H-NS	FNR	FlhDC	H-NS	FNR
fliE	b1937	FlhDC	NtrC	AgaR	FlhDC	H-NS	FNR	FlhDC		
yedN	b1934	NtrC	FlhDC	H-NS	FlhDC	CpxR	H-NS	FlhDC	H-NS	
grxA	b0849	OxyR	MetJ	UlaR	OxyR	CRP	FNR	OxyR		
b1933	b1933	rpoH	H-NS	IHF	FlhDC	H-NS	CpxR	H-NS		
ybjG	b0841	PhoP	UlaR	MarA	PhoP	ArcA	IHF	PhoP		
deoR	b0840	PhoP	Nac	NarL	PhoP	PhoB	Rob	PhoP		
yedE	b1929	FNR	CpxR	IHF	CRP	IHF	FNR	FNR	IHF	
amyA	b1927	OmpR	RcsAB	NtrC	NtrC	Lrp	IHF	NtrC		
fliT	b1926	FlhDC	CpxR	GadE	FlhDC	H-NS	FNR	FlhDC		
fliS	b1925	FlhDC	CpxR	GadE	FlhDC	H-NS	FNR	FlhDC		
fliD	b1924	FlhDC	CpxR	GadE	FlhDC	H-NS	FNR	FlhDC		
dacC	b0839	PhoP	FNR	CRP	FNR	PhoB	ArcA	FNR		
fliC	b1923	GadE	H-NS	FlhDC	GadE	H-NS	CpxR	GadE	H-NS	
fliA	b1922	FlhDC	H-NS	CpxR	FlhDC	H-NS	CpxR	FlhDC	H-NS	CpxR
fliZ	b1921	FlhDC	H-NS	CpxR	FlhDC	H-NS	FNR	FlhDC	H-NS	
b0836	b0836	NarL	FNR	ArcA	ArcA	FNR	CRP	FNR	ArcA	
fliY	b1920	H-NS	FlhDC	IHF	H-NS	FlhDC	IHF	H-NS	FlhDC	IHF
yedO	b1919	H-NS	Lrp	FNR	FlhDC	Lrp	FNR	Lrp	FNR	
uvrY	b1914	LexA	AsnC	GadX	LexA	CRP	CpxR	LexA		

uvrC	b1913	LexA	rpoH	PurR	LexA	CpxR	rpoH	LexA	rpoH	
ybiK	b0828	CysB	IHF	CRP	IHF	FlhDC	H-NS	IHF		
moeA	b0827	NarL	PrpR	OxyR	NarL	FNR	rpoH	NarL		
ybiT	b0820	rpoH	PhoB	FNR	FadR	Fis	rpoH	rpoH		
yecA	b1908	PhoB	FlhDC	CRP	Fis	PhoB	ArcA	PhoB		
tyrP	b1907	HU	TyrR	PdhR	HU	TyrR	Fur	HU	TyrR	
ftn	b1905	Fur	IHF	FNR	IHF	CRP	FNR	IHF	FNR	
yecI	b1902	CpxR	HcaR	ArgR	CpxR	CRP	FNR	CpxR		
araF	b1901	AraC	GntR	GalR	CRP	AraC	H-NS	AraC		
araG	b1900	AraC	RcsAB	GadE	CRP	AraC	H-NS	AraC		
ybiP	b0815	H-NS	Fur	Fis	Fur	FlhDC	NarL	Fur		
ompX	b0814	FNR	CRP	IHF	FNR	CRP	IHF	FNR	CRP	IHF
dps	b0812	OxyR	IHF	FNR	OxyR	IHF	CRP	OxyR	IHF	
glnH	b0811	NtrC	IHF	CRP	NtrC	IHF	Fis	NtrC	IHF	
glnP	b0810	NtrC	IHF	CRP	NtrC	IHF	Fis	NtrC	IHF	
glnQ	b0809	NtrC	Fis	IHF	NtrC	Fis	IHF	NtrC	Fis	IHF
b0805	b0805	Fur	CRP	ArgR	Fur	CRP	IHF	Fur	CRP	
prlC	b3498	CRP	rpoH	LexA	rpoH	Fis	IHF	rpoH		
uspA	b3495	FadR	CRP	Fis	FadR	CRP	IHF	FadR	CRP	
pitA	b3493	ArgR	FruR	Fis	FNR	Fis	IHF	Fis		
yhiN	b3492	FruR	CpxR	FlhDC	rpoH	LexA	CpxR	CpxR		
nikE	b3480	NhaR	NikR	MprA	NarL	NikR	NarP	NikR		
nupC	b2393	Nac	CytR	IHF	Nac	CytR	CRP	Nac	CytR	
b2392	b2392	Fur	IHF	CRP	Fur	IHF	CRP	Fur	IHF	CRP
b2390	b2390	Fur	CpxR	IHF	RcsAB	CpxR	FlhDC	CpxR		
nikD	b3479	NikR	NarL	GntR	NarL	NikR	FNR	NikR	NarL	
nikC	b3478	NikR	IscR	NarP	NarL	NikR	FNR	NikR		
nikB	b3477	NikR	NarP	NarL	NarL	NikR	FlhDC	NikR	NarL	
nikA	b3476	NikR	NarL	FNR	NarL	NikR	FNR	NikR	NarL	FNR
glk	b2388	FruR	Fis	H-NS	FruR	FNR	CRP	FruR		
ycjC	b1299	PhoB	NtrC	CdaR	NtrC	PhoB	Fur	PhoB	NtrC	
ycjL	b1298	CRP	IHF	FNR	NtrC	IHF	Fis	IHF		
b1297	b1297	CRP	FNR	IHF	NtrC	IHF	Fis	IHF		
b2379	b2379	PhoB	PurR	FruR	PhoB	SoxS	Rob	PhoB		
ddg	b2378	Fis	H-NS	CRP	FNR	CRP	IHF	CRP		
rpoH	b3461	CytR	DnaA	IHF	CytR	rpoH	LexA	CytR		
livJ	b3460	Lrp	CRP	IHF	Lrp	CRP	IHF	Lrp	CRP	IHF
b2374	b2374	NanR	PhoP	CpxR	CpxR	FNR	H-NS	CpxR		
fabI	b1288	FruR	CpxR	FNR	Fis	FruR	rpoH	FruR		
yciW	b1287	CysB	IHF	CRP	IHF	CRP	H-NS	IHF	CRP	
evgS	b2370	EvgA	HyfR	BaeR	EvgA	PhoB	AgaR	EvgA		
rnb	b1286	Fis	CRP	FNR	CRP	IHF	FNR	CRP	FNR	
yciR	b1285	Fur	GadE	NtrC	CpxR	NtrC	Fur	Fur	NtrC	
abc	b0199	MetJ	CRP	IHF	MetJ	CRP	IHF	MetJ	CRP	IHF
osmB	b1283	RcsAB	IHF	CRP	RcsAB	CRP	H-NS	RcsAB	CRP	
yaeE	b0198	MetJ	rpoH	FNR	MetJ	rpoH	CRP	MetJ	rpoH	
yaeC	b0197	MetJ	CRP	NtrC	MetJ	IHF	CRP	MetJ	CRP	
pyrF	b1281	Nac	Fis	PurR	Fis	CRP	IHF	Fis		
rcsF	b0196	CRP	FNR	Fis	CpxR	Lrp	Fis	Fis		
cutF	b0192	MtlR	OxyR	PhoP	PhoP	CpxR	Cbl	PhoP		
livK	b3458	Lrp	IHF	CRP	Lrp	CRP	IHF	Lrp	IHF	CRP
livH	b3457	Lrp	MetJ	ArgR	Lrp	TyrR	TrpR	Lrp		
livM	b3456	Lrp	IHF	CRP	Lrp	TyrR	IHF	Lrp	IHF	
livG	b3455	Lrp	IHF	PhoB	Lrp	IHF	CRP	Lrp	IHF	
livF	b3454	Lrp	IHF	ArgR	Lrp	TrpR	IHF	Lrp	IHF	
ugpB	b3453	PhoB	CRP	ArcA	PhoB	NtrC	Fis	PhoB		
evgA	b2369	EvgA	LexA	CpxR	EvgA	CRP	H-NS	EvgA		
ugpA	b3452	PhoB	FruR	Lrp	PhoB	NtrC	FruR	PhoB	FruR	
ugpE	b3451	PhoB	GntR	AraC	PhoB	NtrC	Fis	PhoB		

emrY	b2367	EvgA	AllR	XylR	EvgA	NagC	CpxR	EvgA		
ugpC	b3450	SoxS	PhoB	SdiA	PhoB	PrpR	Cbl	PhoB		
dsdA	b2366	FlhDC	CRP	FNR	FlhDC	Fur	CpxR	FlhDC		
dsdC	b2364	LexA	MetJ	FlhDC	FlhDC	Rob	CpxR	FlhDC		
ribA	b1277	SoxS	DeoR	LldR	SoxS	Fis	FNR	SoxS		
acnA	b1276	FruR	ArcA	IHF	FruR	NtrC	Fis	FruR		
cysB	b1275	CysB	CpxR	H-NS	CysB	FlhDC	FNR	CysB		
topA	b1274	rpoH	Fis	FlhDC	rpoH	SoxS	Fis	rpoH	Fis	
ldcC	b0186	LsrR	NtrC	AppY	Lrp	NtrC	OxyR	NtrC		
accA	b0185	rpoH	FlhDC	CpxR	CpxR	Fis	Fur	CpxR		
dnaE	b0184	FNR	H-NS	Fis	CpxR	Fis	ArgR	Fis		
rnhB	b0183	LexA	FlhDC	H-NS	Fis	CpxR	FlhDC	FlhDC		
lpxA	b0181	GadE	ArgR	CpxR	CpxR	ArgR	GadE	GadE	ArgR	CpxR
fabZ	b0180	GadE	CpxR	Fis	CpxR	ArgR	FNR	CpxR		
ugpQ	b3449	SoxS	MarA	PhoB	PhoB	CysB	Nac	PhoB		
yhhA	b3448	Fis	FNR	CRP	CRP	IHF	FNR	FNR	CRP	
yfdN	b2357	AgaR	RcsAB	MngR	ArgR	RcsAB	rpoH	RcsAB		
b2351	b2351	H-NS	Fur	IHF	CRP	rpoH	IHF	IHF		
yciV	b1266	Nac	LexA	PhoB	HyfR	PhoB	FhlA	PhoB		
trpE	b1264	TrpR	IHF	ArcA	TrpR	Lrp	CpxR	TrpR		
lpxD	b0179	GadE	CpxR	H-NS	CpxR	GadE	Fis	GadE	CpxR	
trpD	b1263	TrpR	IHF	ArcA	TrpR	Lrp	CRP	TrpR		
hlpA	b0178	CpxR	GadE	NarL	CpxR	GadE	rpoH	CpxR	GadE	
trpC	b1262	TrpR	IHF	TyrR	TrpR	Lrp	IHF	TrpR	IHF	
yaeT	b0177	rpoH	ArcA	CRP	H-NS	CRP	Fis	CRP		
trpB	b1261	TrpR	IHF	TyrR	TrpR	Lrp	CRP	TrpR		
yaeL	b0176	rpoH	Fis	ArgR	CpxR	Fis	H-NS	Fis		
trpA	b1260	TrpR	IHF	CRP	TrpR	CRP	Lrp	TrpR	CRP	
uppS	b0174	Fis	CRP	IHF	Fis	CRP	H-NS	Fis	CRP	
dxr	b0173	Nac	CpxR	Lrp	CpxR	PurR	TrpR	CpxR		
frr	b0172	LexA	FNR	rpoH	CpxR	Fur	FNR	FNR		
pyrH	b0171	FNR	Fis	CRP	ArgR	IHF	FNR	FNR		
tsf	b0170	FNR	Fis	ArcA	CRP	FNR	Fis	FNR	Fis	
yhhW	b3439	PhoP	Cbl	Fur	CysB	Cbl	CpxR	Cbl		
gntK	b3437	GntR	TyrR	IdnR	GntR	IdnR	CRP	GntR	IdnR	
asd	b3433	CRP	IHF	Lrp	Lrp	CRP	FNR	CRP	Lrp	
glgB	b3432	FNR	IHF	CRP	CRP	FNR	Lrp	FNR	CRP	
glgX	b3431	FNR	ArcA	IHF	CRP	FNR	Lrp	FNR		
yfdC	b2347	NtrC	FlhDC	CaiF	NtrC	RcsAB	SoxS	NtrC		
glgC	b3430	FNR	CRP	IHF	CRP	Lrp	FNR	FNR	CRP	
vacJ	b2346	FruR	GadX	PhoP	Lrp	CpxR	FruR	FruR		
fadL	b2344	PhoP	OmpR	FadR	FadR	PhoP	OmpR	PhoP	OmpR	FadR
yciG	b1259	H-NS	FNR	IHF	H-NS	CRP	Lrp	H-NS		
b2342	b2342	FadR	ArcA	CRP	FadR	DnaA	FlhDC	FadR		
yciF	b1258	H-NS	FNR	Fis	H-NS	CRP	FNR	H-NS	FNR	
b2341	b2341	FadR	ArcA	CRP	FadR	Fis	ArcA	FadR	ArcA	
b2340	b2340	CpxR	NtrC	H-NS	CpxR	Fur	Lrp	CpxR		
yciD	b1256	FNR	Fur	ArcA	FNR	CRP	ArcA	FNR	ArcA	
rpsB	b0169	FNR	Fis	ArcA	FNR	CRP	Fis	FNR	Fis	
tonB	b1252	Fur	FNR	IHF	Fur	IHF	CRP	Fur	IHF	
dapD	b0166	CpxR	ArgR	FNR	CpxR	CRP	Lrp	CpxR		
yaeG	b0162	CdaR	CRP	FNR	CdaR	FadR	FlhDC	CdaR		
htrA	b0161	CpxR	H-NS	CRP	CpxR	H-NS	Fur	CpxR	H-NS	
dgt	b0160	Zur	HcaR	PhoB	PhoB	CysB	NarL	PhoB		
glgA	b3429	FNR	IHF	CRP	CRP	FNR	Lrp	FNR	CRP	
yfcU	b2338	GntR	FhlA	PhoP	NagC	LexA	GntR	GntR		
cls	b1249	PhoB	DnaA	PhoP	PhoP	Lrp	rpoH	PhoP		
oppF	b1247	ModE	Lrp	ArcA	ModE	Lrp	ArcA	ModE	Lrp	ArcA
oppD	b1246	ModE	Lrp	ArcA	ModE	Lrp	ArcA	ModE	Lrp	ArcA

oppC	b1245	ModE	Lrp	ArcA	ModE	Lrp	ArcA	ModE	Lrp	ArcA
oppB	b1244	ModE	Lrp	ArcA	ModE	Lrp	ArcA	ModE	Lrp	ArcA
oppA	b1243	ModE	Lrp	ArcA	ModE	Lrp	ArcA	ModE	Lrp	ArcA
adhE	b1241	FruR	NarL	Fis	FruR	NarL	Fis	FruR	NarL	Fis
hemL	b0154	PhoP	CdaR	Nac	PhoP	ArgR	Lrp	PhoP		
fhuB	b0153	Fur	SoxS	NagC	Fur	OxyR	FhlA	Fur		
fhuD	b0152	Fur	CpxR	NarL	Fur	FlhDC	CpxR	Fur	CpxR	
fhuC	b0151	Cbl	Fur	FhlA	Fur	Rob	SoxS	Fur		
fhuA	b0150	Fur	FNR	CRP	Fur	CRP	FNR	Fur	FNR	CRP
malT	b3418	DgsA	Lrp	CRP	Lrp	DgsA	CRP	DgsA	Lrp	CRP
malP	b3417	MalT	FNR	CRP	MalT	FNR	CRP	MalT	FNR	CRP
malQ	b3416	MalT	FNR	FruR	MalT	FNR	CRP	MalT	FNR	
gntT	b3415	GntR	PhoB	CRP	GntR	FlhDC	CRP	GntR	CRP	
yhgI	b3414	GntR	IscR	CRP	GntR	IscR	CRP	GntR	IscR	CRP
fabB	b2323	FadR	CRP	IHF	FadR	FNR	CpxR	FadR		
hns	b1237	GadX	H-NS	Fis	GadX	Fis	H-NS	GadX	H-NS	Fis
galU	b1236	SoxS	CpxR	ModE	CpxR	Fis	FruR	CpxR		
sfsA	b0146	NarL	H-NS	IHF	H-NS	CRP	FNR	H-NS		
dksA	b0145	HU	Fis	H-NS	FNR	CRP	Fis	Fis		
pcnB	b0143	CRP	FNR	H-NS	rpoH	FNR	IHF	FNR		
feoB	b3409	Fur	FNR	NarL	Fur	FNR	CRP	Fur	FNR	
feoA	b3408	Fur	FNR	H-NS	Fur	FNR	CRP	Fur	FNR	
ompR	b3405	ArgR	UxuR	Fur	CpxR	ArgR	NarL	ArgR		
pckA	b3403	FruR	IHF	CRP	FruR	CRP	FNR	FruR	CRP	
hslO	b3401	rpoH	CRP	Fis	rpoH	IHF	LexA	rpoH		
hslR	b3400	rpoH	CRP	Fis	rpoH	IHF	CRP	rpoH	CRP	
accD	b2316	LexA	PhoP	Fis	LexA	Fur	PurR	LexA		
cvpA	b2313	PurR	PhoP	CRP	PurR	FNR	CRP	PurR	CRP	
purF	b2312	PurR	PhoP	CRP	PurR	Lrp	FNR	PurR		
b1228	b1228	FNR	Fis	PhoB	HyfR	Fis	FhlA	Fis		
narI	b1227	NarL	CpxR	IHF	NarL	IHF	FNR	NarL	IHF	
argT	b2310	NtrC	Fis	IHF	NtrC	Fis	CRP	NtrC	Fis	
narJ	b1226	NarL	IHF	FNR	NarL	IHF	FNR	NarL	IHF	FNR
narH	b1225	NarL	IHF	FNR	NarL	IHF	FNR	NarL	IHF	FNR
narG	b1224	NarL	IHF	FNR	NarL	IHF	FNR	NarL	IHF	FNR
narK	b1223	NarL	IHF	FNR	NarL	IHF	FNR	NarL	IHF	FNR
narX	b1222	ModE	UlaR	CdaR	ModE	NarP	NarL	ModE		
narL	b1221	ModE	Fis	CRP	ModE	FNR	IHF	ModE		
panD	b0131	IHF	Fis	CRP	CpxR	Lrp	CRP	CRP		
hisJ	b2309	NtrC	IHF	ArgR	NtrC	CRP	IHF	NtrC	IHF	
hisQ	b2308	NtrC	NagC	AscG	NtrC	IHF	Lrp	NtrC		
hisM	b2307	NtrC	MetJ	ArgR	NtrC	Lrp	IHF	NtrC		
hisP	b2306	NtrC	LeuO	MetJ	NtrC	ArgR	PhoP	NtrC		
chaB	b1217	ModE	SoxS	PhoB	CpxR	Fur	PhoB	PhoB		
chaA	b1216	CpxR	NhaR	Fis	CRP	rpoH	CpxR	CpxR		
yfcE	b2300	Lrp	SoxS	Fis	Fis	FNR	CRP	Fis		
yadI	b0129	PhoB	CdaR	CytR	CytR	FNR	FlhDC	CytR		
prfA	b1211	CpxR	RcsAB	ArcA	IHF	ArcA	FNR	ArcA		
hemA	b1210	FNR	RcsAB	IHF	IHF	ArcA	FNR	FNR	IHF	
speE	b0121	CRP	FNR	ArcA	Fis	CRP	IHF	CRP		
speD	b0120	FNR	CRP	PurR	Fis	CRP	IHF	CRP		
yacL	b0119	CRP	Fur	NarL	CRP	CpxR	rpoH	CRP		
ychF	b1203	Fis	FNR	CRP	Lrp	Fis	CRP	Fis	CRP	
acnB	b0118	FruR	Fis	ArcA	FruR	Fis	ArcA	FruR	Fis	ArcA
lpdA	b0116	PdhR	Fis	FNR	Fis	ArcA	FNR	Fis	FNR	
b1200	b1200	DhaR	IHF	CRP	DhaR	CRP	FNR	DhaR	CRP	
aceF	b0115	PdhR	ArcA	FNR	ArcA	FNR	CRP	ArcA	FNR	
aceE	b0114	PdhR	ArcA	FNR	ArcA	FNR	CRP	ArcA	FNR	
pdhR	b0113	PdhR	FNR	CRP	FNR	Fis	CRP	FNR	CRP	

aroP	b0112	TyrR	CRP	IHF	TyrR	Lrp	CRP	TyrR	CRP	
guaC	b0104	ArgR	IHF	FNR	CpxR	Fur	ArgR	ArgR		
b2997	b2997	NarL	ArcA	Fur	NarL	ArcA	FNR	NarL	ArcA	
hybA	b2996	NarL	NarP	TorR	NarL	ArcA	FNR	NarL		
hybB	b2995	NarL	CaiF	LexA	NarL	ArcA	CRP	NarL		
hybC	b2994	NarL	FhlA	NarP	NarL	ArcA	FNR	NarL		
hybG	b2990	NhaR	NarL	Nac	NarL	ArcA	NarP	NarL		
araH_1	b1899	CsgD	CytR	AraC	CRP	AraC	FNR	AraC		
otsB	b1897	SoxS	Fis	IHF	H-NS	CRP	IHF	IHF		
glcC	b2980	GlcC	CRP	IHF	CRP	ArcA	GlcC	GlcC	CRP	
otsA	b1896	MarA	SoxS	H-NS	Lrp	SoxS	IHF	SoxS		
flhD	b1892	LrhA	RcsAB	OmpR	QseB	RcsAB	Fur	RcsAB		
flhC	b1891	LrhA	RcsAB	OmpR	QseB	RcsAB	Fur	RcsAB		
motA	b1890	CpxR	FlhDC	H-NS	CpxR	FlhDC	GadE	CpxR	FlhDC	
glcD	b2979	GlcC	ArcA	IHF	IHF	ArcA	CRP	ArcA	IHF	
glcF	b2978	ArcA	IHF	CRP	IHF	ArcA	Fis	ArcA	IHF	
glcG	b2977	GlcC	ArcA	IHF	IHF	ArcA	Lrp	ArcA	IHF	
glcB	b2976	GlcC	ArcA	IHF	IHF	ArcA	CRP	ArcA	IHF	
motB	b1889	CpxR	FlhDC	H-NS	CpxR	FlhDC	FNR	CpxR	FlhDC	
cheA	b1888	CpxR	H-NS	GadE	CpxR	FNR	FlhDC	CpxR		
cheW	b1887	CpxR	H-NS	FNR	CpxR	FNR	FlhDC	CpxR	FNR	
b2970	b2970	AllR	rpoH	NtrC	LexA	rpoH	FadR	rpoH		
tar	b1886	CpxR	FNR	FlhDC	FlhDC	FNR	CpxR	CpxR	FNR	FlhDC
tap	b1885	FNR	FlhDC	CpxR	CpxR	FlhDC	FNR	FNR	FlhDC	CpxR
cheR	b1884	FlhDC	FNR	CpxR	CpxR	FlhDC	FNR	FlhDC	FNR	CpxR
dinG	b0799	LexA	TyrR	CpxR	LexA	CRP	ArcA	LexA		
cheB	b1883	FNR	FlhDC	CpxR	CpxR	FNR	FlhDC	FNR	FlhDC	CpxR
cheY	b1882	CpxR	FNR	FlhDC	CpxR	FNR	FlhDC	CpxR	FNR	FlhDC
rhlE	b0797	FlhDC	CRP	Fis	Fur	Fis	CRP	CRP	Fis	
cheZ	b1881	FNR	FlhDC	CpxR	CpxR	FNR	FlhDC	FNR	FlhDC	CpxR
flhB	b1880	FlhDC	FNR	NarL	FlhDC	CpxR	H-NS	FlhDC		
nupG	b2964	CytR	DeoR	EvgA	CytR	CRP	Fis	CytR		
mltC	b2963	H-NS	Fur	FruR	FruR	FlhDC	CpxR	FruR		
flhA	b1879	FlhDC	IHF	CRP	FlhDC	CpxR	H-NS	FlhDC		
flhE	b1878	FlhDC	RcsAB	FNR	FlhDC	CpxR	H-NS	FlhDC		
mutY	b2961	MarA	PhoP	LexA	LexA	CpxR	ArgR	LexA		
yggH	b2960	FNR	H-NS	Fur	Fis	FNR	CpxR	FNR		
ybhO	b0789	MarA	NarL	PhoB	SoxS	MarA	NtrC	MarA		
moaE	b0785	CueR	ModE	PhoP	ModE	FNR	NarL	ModE		
moaD	b0784	CueR	ModE	HU	ModE	FNR	IHF	ModE		
moaC	b0783	CueR	ModE	CpxR	ModE	FNR	IHF	ModE		
moaB	b0782	CueR	ModE	PhoP	ModE	FNR	CueR	CueR	ModE	
moaA	b0781	CueR	ModE	EnvY	ModE	FNR	IHF	ModE		
ybhK	b0780	ArcA	H-NS	LexA	PhoB	Rob	LexA	LexA		
yggN	b2958	FlhDC	CpxR	FruR	SoxS	CpxR	NagC	CpxR		
yggT	b2952	rpoH	PhoB	AraC	FlhDC	CpxR	rpoH	rpoH		
aspS	b1866	FNR	Fis	CRP	PurR	Fis	FNR	FNR	Fis	
yebC	b1864	ArgR	NarL	Fis	Fis	CRP	PurR	Fis		
uvrB	b0779	LexA	ArcA	IHF	LexA	CRP	ArcA	LexA	ArcA	
ruvA	b1861	LexA	CpxR	SoxS	LexA	CRP	FNR	LexA		
ruvB	b1860	LexA	GcvA	NagC	LexA	FhlA	CdaR	LexA		
bioB	b0775	CRP	BirA	CpxR	IHF	CRP	FNR	CRP		
gshB	b2947	HU	CpxR	NarL	SoxS	CpxR	Lrp	CpxR		
galP	b2943	GalR	GalS	CRP	GalR	GalS	CRP	GalR	GalS	CRP
metK	b2942	MetJ	CRP	FNR	MetJ	CRP	FNR	MetJ	CRP	FNR
yebL	b1857	Zur	HcaR	TyrR	Zur	CRP	FNR	Zur		
pykA	b1854	NarL	Fur	rpoH	IHF	NarL	FNR	NarL		
zwf	b1852	Rob	MarA	SoxS	Rob	SoxS	MarA	Rob	MarA	SoxS
ybhE	b0767	OxyR	IHF	FNR	H-NS	SoxS	IHF	IHF		

edd	b1851	GntR	FruR	NarP	GntR	FruR	PhoB	GntR	FruR	
ybhA	b0766	HU	H-NS	PhoB	Fur	H-NS	CRP	H-NS		
eda	b1850	GntR	QseB	DgsA	GntR	PhoB	FruR	GntR		
modC	b0765	ModE	CRP	FhlA	ModE	FlhDC	CpxR	ModE		
modB	b0764	ModE	GntR	MprA	ModE	CRP	H-NS	ModE		
modA	b0763	ModE	CRP	IHF	ModE	CRP	IHF	ModE	CRP	IHF
modF	b0760	Fur	CpxR	NtrC	Fur	CRP	H-NS	Fur		
speA	b2938	PurR	H-NS	CRP	PurR	Fis	FNR	PurR		
speB	b2937	PurR	CpxR	Fis	PurR	CpxR	FNR	PurR	CpxR	
purT	b1849	CRP	FNR	IHF	PurR	CRP	Lrp	CRP		
yebG	b1848	CpxR	LexA	H-NS	LexA	CRP	ArcA	LexA		
yebF	b1847	CpxR	LexA	H-NS	LexA	ArcA	CRP	LexA		
yebE	b1846	CpxR	IHF	Fur	CpxR	CRP	ArcA	CpxR		
ptrB	b1845	NagC	BaeR	ExuR	CpxR	NagC	FlhDC	NagC		
galE	b0759	HU	GalR	GalS	HU	GalR	GalS	HU	GalR	GalS
galT	b0758	HU	GalR	GalS	HU	GalR	GalS	HU	GalR	GalS
holE	b1842	Fis	H-NS	rpoH	CRP	LexA	H-NS	H-NS		
galK	b0757	HU	GalR	GalS	HU	GalR	GalS	HU	GalR	GalS
b1841	b1841	H-NS	PhoB	Fis	Fis	NarL	Fur	Fis		
galM	b0756	HU	GalR	GalS	HU	GalR	GalS	HU	GalR	GalS
b1840	b1840	H-NS	PhoB	CRP	Fis	IHF	CRP	CRP		
gpmA	b0755	Fur	PhoP	GadE	Fur	CRP	FNR	Fur		
aroG	b0754	TyrR	CpxR	Nac	TyrR	CpxR	CRP	TyrR	CpxR	
b0753	b0753	OmpR	MarA	NtrC	ArcA	NtrC	IHF	NtrC		
nadA	b0750	CRP	ArcA	IHF	CRP	IHF	FNR	CRP	IHF	
epd	b2927	FruR	NarL	Fis	FruR	CRP	rpoH	FruR		
pgk	b2926	FruR	NarL	CRP	FruR	CRP	Fis	FruR	CRP	
fba	b2925	FruR	CRP	FNR	FruR	rpoH	Fis	FruR		
yggB	b2924	CRP	FNR	H-NS	CRP	FNR	IHF	CRP	FNR	
yggE	b2922	IHF	FNR	CRP	FNR	CRP	IHF	IHF	FNR	CRP
pphA	b1838	rpoH	OxyR	PhoP	UxuR	rpoH	ArgR	rpoH		
ygfH	b2920	NagC	SoxS	GntR	RcsAB	FadR	NagC	NagC		
b1836	b1836	H-NS	IHF	FNR	IHF	H-NS	CRP	H-NS	IHF	
yebU	b1835	Fis	GadE	PurR	SoxS	PurR	MarA	PurR		
prc	b1830	Fis	rpoH	CRP	Fis	IHF	CRP	Fis	CRP	
ybgF	b0742	Fis	CpxR	Lrp	FNR	CpxR	CRP	CpxR		
ygfG	b2919	YiaJ	EvgA	AllR	AgaR	EvgA	PhoB	EvgA		
iciA	b2916	PhoB	FlhDC	TyrR	PhoB	FhlA	ArgR	PhoB		
rpiA	b2914	FadR	MetR	rpoH	SoxS	rpoH	CpxR	rpoH		
serA	b2913	Nac	Lrp	CRP	Nac	Lrp	CRP	Nac	Lrp	CRP
htpX	b1829	rpoH	IHF	FNR	rpoH	CRP	IHF	rpoH	IHF	
ygfA	b2912	H-NS	CpxR	rpoH	CpxR	PhoB	FadR	CpxR		
b1825	b1825	NarL	IHF	FNR	Fis	FNR	CRP	FNR		
cspC	b1823	CpxR	FNR	PhoB	CRP	Fis	FNR	FNR		
tolR	b0738	PhoP	ArgR	FlhDC	FlhDC	Fis	Fur	FlhDC		
b1821	b1821	rpoH	MalT	H-NS	CsgD	FruR	rpoH	rpoH		
ybgC	b0736	H-NS	Fis	NtrC	CRP	H-NS	CpxR	H-NS		
ybgE	b0735	FruR	H-NS	FNR	FruR	ArcA	FNR	FruR	FNR	
cydB	b0734	FruR	H-NS	ArcA	FruR	H-NS	ArcA	FruR	H-NS	ArcA
cydA	b0733	FruR	H-NS	ArcA	FruR	H-NS	ArcA	FruR	H-NS	ArcA
pepP	b2908	CpxR	LexA	NarL	CpxR	rpoH	PurR	CpxR		
gcvT	b2905	PurR	GcvA	Lrp	PurR	Lrp	GcvA	PurR	GcvA	Lrp
gcvH	b2904	PurR	GcvA	Lrp	Lrp	PurR	GcvA	PurR	GcvA	Lrp
gcvP	b2903	GcvA	PurR	Lrp	PurR	Lrp	FNR	PurR	Lrp	
manZ	b1819	NagC	DgsA	CRP	NagC	DgsA	CRP	NagC	DgsA	CRP
manY	b1818	NagC	DgsA	CRP	NagC	DgsA	CRP	NagC	DgsA	CRP
manX	b1817	NagC	DgsA	CRP	NagC	DgsA	CRP	NagC	DgsA	CRP
sdaA	b1814	Lrp	IHF	CRP	Lrp	rpoH	CRP	Lrp	CRP	
sucD	b0729	Fur	ArcA	IHF	Fur	ArcA	IHF	Fur	ArcA	IHF

sucC	b0728	Fur	ArcA	IHF	Fur	ArcA	IHF	Fur	ArcA	IHF
sucB	b0727	Fur	ArcA	IHF	Fur	ArcA	IHF	Fur	ArcA	IHF
sucA	b0726	Fur	ArcA	IHF	Fur	ArcA	IHF	Fur	ArcA	IHF
b1810	b1810	H-NS	NtrC	Fis	H-NS	CRP	IHF	H-NS		
sdhB	b0724	Fur	FNR	FlhDC	Fur	ArcA	FNR	Fur	FNR	
sdhA	b0723	Fur	ArcA	FNR	Fur	ArcA	FNR	Fur	ArcA	FNR
sdhD	b0722	Fur	ArcA	CRP	Fur	ArcA	FNR	Fur	ArcA	
gltA	b0720	ArcA	IHF	CRP	ArcA	IHF	CRP	ArcA	IHF	CRP
b1809	b1809	PhoP	Fur	CRP	Fis	CRP	H-NS	CRP		
fadD	b1805	FadR	ArcA	CRP	FadR	ArcA	CRP	FadR	ArcA	CRP
ybgQ	b0718	NagC	CpxR	RcsAB	RcsAB	NagC	LexA	NagC	RcsAB	
yeaW	b1802	GntR	NagC	CusR	UxuR	NagC	SoxS	NagC		
ybgO	b0716	CdaR	Fur	LexA	rpoH	FlhDC	Fur	Fur		
abrB	b0715	CpxR	MetR	FlhDC	NarL	FlhDC	Fur	FlhDC		
b0703	b0703	Rob	rpoH	CpxR	PhoB	NagC	CpxR	CpxR		
yrfG	b3399	rpoH	GadX	CpxR	rpoH	Fis	IHF	rpoH		
aroB	b3389	Fis	FNR	LexA	Fis	CpxR	FlhDC	Fis		
b2299	b2299	FNR	Lrp	IHF	Fis	IHF	CpxR	IHF		
pta	b2297	FNR	ArcA	CRP	FNR	ArcA	CRP	FNR	ArcA	CRP
ackA	b2296	FruR	ArcA	FNR	ArcA	FNR	CRP	ArcA	FNR	
b2295	b2295	FruR	IHF	rpoH	CpxR	rpoH	FNR	rpoH		
nuoA	b2288	NarL	Fis	IHF	NarL	Fis	ArcA	NarL	Fis	
nuoB	b2287	NarL	Fis	FNR	NarL	Fis	IHF	NarL	Fis	
nuoC	b2286	NarL	Fis	ArcA	NarL	Fis	IHF	NarL	Fis	
nuoE	b2285	NarL	Fis	ArcA	NarL	Fis	IHF	NarL	Fis	
nuoF	b2284	NarL	Fis	ArcA	NarL	Fis	IHF	NarL	Fis	
b1199	b1199	DhaR	Fis	NarL	DhaR	CRP	FNR	DhaR		
nuoG	b2283	NarL	Fis	IHF	NarL	Fis	IHF	NarL	Fis	IHF
ycgC	b1198	DhaR	Fis	CRP	DhaR	CRP	FNR	DhaR	CRP	
nuoH	b2282	NarL	Fis	ArcA	NarL	Fis	IHF	NarL	Fis	
treA	b1197	NtrC	Fis	FNR	NtrC	Nac	PhoB	NtrC		
nuoI	b2281	NarL	Fis	FNR	NarL	Fis	IHF	NarL	Fis	
nuoJ	b2280	NarL	Fis	FNR	NarL	Fis	IHF	NarL	Fis	
ycgR	b1194	FlhDC	CpxR	H-NS	FlhDC	CpxR	FNR	FlhDC	CpxR	
dadX	b1190	Lrp	CRP	FNR	Lrp	CRP	FNR	Lrp	CRP	FNR
cysG	b3368	NarP	FruR	NarL	NarP	FruR	NarL	NarP	FruR	NarL
nirC	b3367	LacI	NarP	UxuR	NarP	NarL	FruR	NarP		
nirD	b3366	AllR	Cbl	NarP	NarP	NarL	FruR	NarP		
nirB	b3365	NarP	NarL	FruR	NarP	FruR	NarL	NarP	NarL	FruR
yhfC	b3364	CRP	Fis	H-NS	CRP	FNR	Fis	CRP	Fis	
ppiA	b3363	CytR	CpxR	NagC	CpxR	CytR	Fis	CytR	CpxR	
nuoK	b2279	NarL	Fis	FNR	NarL	Fis	ArcA	NarL	Fis	
nuoL	b2278	NarL	Fis	ArcA	NarL	Fis	ArcA	NarL	Fis	ArcA
fic	b3361	NtrC	Fis	H-NS	NtrC	Fis	CRP	NtrC	Fis	
nuoM	b2277	NarL	Fis	ArcA	NarL	Fis	IHF	NarL	Fis	
nuoN	b2276	NarL	Fis	IHF	NarL	Fis	IHF	NarL	Fis	IHF
dadA	b1189	Lrp	CRP	IHF	Lrp	CRP	ArcA	Lrp	CRP	
ycgB	b1188	FNR	NtrC	IHF	NtrC	Fis	CRP	NtrC		
fadR	b1187	BaeR	PhoB	FlhDC	NagC	SoxS	FlhDC	FlhDC		
yfbL	b2271	AgaR	FucR	CsgD	AgaR	NagC	RcsAB	AgaR		
dsbB	b1185	LexA	CpxR	Fur	Fis	Lrp	CpxR	CpxR		
umuC	b1184	LexA	CpxR	CRP	LexA	CRP	ArcA	LexA	CRP	
umuD	b1183	LexA	CpxR	H-NS	LexA	CRP	ArcA	LexA		
secA	b0098	ModE	PdhR	NarL	CpxR	FNR	NarL	NarL		
ycgN	b1181	PhoP	CpxR	RcsAB	SoxS	PhoP	Lrp	PhoP		
lpxC	b0096	rpoH	CpxR	FlhDC	CRP	FNR	CpxR	CpxR		
ftsZ	b0095	RcsAB	GntR	OxyR	RcsAB	CpxR	Lrp	RcsAB		
ftsA	b0094	RcsAB	Fis	H-NS	RcsAB	CpxR	FNR	RcsAB		
ftsQ	b0093	RcsAB	Fis	rpoH	RcsAB	rpoH	CpxR	RcsAB	rpoH	

ddlB	b0092	RcsAB	Fis	rpoH	RcsAB	CpxR	ArgR	RcsAB		
murC	b0091	Fur	Fis	FNR	FlhDC	ArgR	Fur	Fur		
argD	b3359	PhoP	ArgR	CRP	ArgR	PhoP	Lrp	PhoP	ArgR	
crp	b3357	FruR	CRP	PdhR	CRP	Lrp	IHF	CRP		
minC	b1176	H-NS	Fis	CpxR	H-NS	CRP	Lrp	H-NS		
b1170	b1170	CpxR	rpoH	NanR	CpxR	PhoB	PhoP	CpxR		
murE	b0085	ModE	GadE	FlhDC	CpxR	rpoH	FlhDC	FlhDC		
ftsI	b0084	CpxR	IclR	FlhDC	SoxS	FlhDC	Fis	FlhDC		
yabB	b0081	H-NS	GadE	Fis	CpxR	rpoH	Fis	Fis		
fkpA	b3347	LexA	CpxR	FlhDC	CpxR	Fis	FNR	CpxR		
yheN	b3345	ModE	CpxR	LexA	CpxR	Lrp	rpoH	CpxR		
yheL	b3343	rpoH	LexA	CpxR	rpoH	IHF	CRP	rpoH		
rpsL	b3342	FNR	ArcA	Fis	FNR	Fis	CpxR	FNR	Fis	
b2258	b2258	PhoB	H-NS	CpxR	FlhDC	CpxR	LexA	CpxR		
rpsG	b3341	FNR	Fis	ArcA	FNR	Fis	ArcA	FNR	Fis	ArcA
b2257	b2257	NarL	Fur	FlhDC	FlhDC	rpoH	CpxR	FlhDC		
fusA	b3340	ArcA	Fis	FNR	FNR	Fis	ArcA	ArcA	Fis	FNR
b1169	b1169	Fis	FNR	CRP	CpxR	CRP	FNR	FNR	CRP	
b1168	b1168	CpxR	FNR	Fis	CpxR	rpoH	Fis	CpxR	Fis	
ymgA	b1165	CpxR	FNR	IHF	CRP	IHF	Lrp	IHF		
ycgZ	b1164	CpxR	FNR	IHF	IHF	CRP	Fis	IHF		
b1163	b1163	CRP	IHF	Fis	CpxR	FNR	CRP	CRP		
ilvH	b0078	Lrp	OmpR	H-NS	Lrp	H-NS	PurR	Lrp	H-NS	
ilvI	b0077	Lrp	ArgR	GadE	Lrp	H-NS	CRP	Lrp		
leuA	b0074	LeuO	Lrp	CRP	Lrp	CRP	IHF	Lrp	CRP	
leuB	b0073	LeuO	Lrp	CRP	CRP	Lrp	IHF	Lrp	CRP	
leuC	b0072	LeuO	Lrp	CRP	Lrp	CRP	IHF	Lrp	CRP	
leuD	b0071	LeuO	Lrp	CRP	Lrp	CRP	IHF	Lrp	CRP	
tufA	b3339	Fis	ArcA	FNR	Fis	FNR	ArcA	Fis	ArcA	FNR
bfr	b3336	Fur	Ada	FNR	CRP	ArcA	FNR	FNR		
yheJ	b3332	Rob	SoxS	GntR	RcsAB	YiaJ	GntR	GntR		
b2248	b2248	PhoP	NarL	FlhDC	RcsAB	rpoH	FlhDC	FlhDC		
b2247	b2247	RcsAB	GalR	GalS	NagC	RcsAB	Rob	RcsAB		
b2246	b2246	AllR	RcsAB	ModE	UxuR	RcsAB	MarA	RcsAB		
yfaD	b2244	CsgD	RcsAB	GadX	RcsAB	rpoH	FlhDC	RcsAB		
glpC	b2243	FlhDC	FNR	ArcA	FlhDC	ArcA	FNR	FlhDC	FNR	ArcA
glpB	b2242	FlhDC	CRP	ArcA	FlhDC	ArcA	FNR	FlhDC	ArcA	
glpA	b2241	FlhDC	FNR	ArcA	FlhDC	ArcA	FNR	FlhDC	FNR	ArcA
glpT	b2240	CRP	ArcA	GlpR	IHF	CRP	FNR	CRP		
yabK	b0067	FhlA	AllR	NarP	NtrC	NarP	NarL	NarP		
araC	b0064	AraC	DcuR	NarL	AraC	CRP	FNR	AraC		
araB	b0063	AraC	CaiF	MngR	AraC	CRP	H-NS	AraC		
araD	b0061	MngR	AraC	NhaR	CRP	AraC	H-NS	AraC		
polB	b0060	LexA	FlhDC	IHF	LexA	CRP	ArcA	LexA		
glpQ	b2239	GlpR	CRP	FNR	IHF	CRP	FNR	CRP	FNR	
rpsJ	b3321	ArcA	FNR	Fis	FNR	ArcA	Fis	ArcA	FNR	Fis
inaA	b2237	Rob	MarA	BetI	Rob	SoxS	MarA	Rob	MarA	
rplC	b3320	ArcA	FNR	Fis	ArcA	FNR	Fis	ArcA	FNR	Fis
yfaE	b2236	ArgP	AraC	DnaA	DnaA	Fis	CRP	DnaA		
nrdB	b2235	DnaA	Fis	CRP	Fis	DnaA	CRP	DnaA	Fis	CRP
nrdA	b2234	DnaA	Fis	ArgP	Fis	DnaA	CRP	DnaA	Fis	
gyrA	b2231	Fis	CRP	Lrp	Fis	CRP	PurR	Fis	CRP	
yabO	b0058	OmpR	PurR	rpoH	PurR	IHF	CRP	PurR		
surA	b0053	NarL	Fis	IHF	Fis	H-NS	CRP	Fis		
pdxA	b0052	Fis	FlhDC	NarL	Fis	H-NS	PhoP	Fis		
ksgA	b0051	LexA	Fis	ModE	Fis	NagC	rpoH	Fis		
rplD	b3319	FNR	ArcA	Fis	ArcA	FNR	Fis	FNR	ArcA	Fis
yjjY	b4402	FruR	FNR	CRP	CpxR	H-NS	FNR	FNR		
rplW	b3318	ArcA	FNR	Fis	ArcA	FNR	Fis	ArcA	FNR	Fis

arcA	b4401	FNR	CRP	IHF	FNR	ArcA	CRP	FNR	CRP	
rplB	b3317	FNR	ArcA	Fis	ArcA	FNR	Fis	FNR	ArcA	Fis
rpsS	b3316	ArcA	FNR	Fis	ArcA	FNR	Fis	ArcA	FNR	Fis
rplV	b3315	ArcA	FNR	Fis	ArcA	FNR	Fis	ArcA	FNR	Fis
rpsC	b3314	ArcA	FNR	Fis	FNR	ArcA	Fis	ArcA	FNR	Fis
rplP	b3313	ArcA	FNR	Fis	FNR	ArcA	Fis	ArcA	FNR	Fis
b2228	b2228	FruR	NarL	GntR	NagC	GntR	TrpR	GntR		
rplN	b3310	FNR	ArcA	Fis	FNR	ArcA	Fis	FNR	ArcA	Fis
lit	b1139	MalT	CaiF	CpxR	CRP	CpxR	rpoH	CpxR		
icdA	b1136	FruR	ArcA	CRP	FruR	ArcA	CRP	FruR	ArcA	CRP
b1134	b1134	PurR	CpxR	rpoH	LexA	PurR	NagC	PurR		
ycfC	b1132	PurR	DgsA	CRP	PurR	FNR	Fis	PurR		
purB	b1131	PurR	CRP	FNR	PurR	Fis	CRP	PurR	CRP	
phoP	b1130	PhoP	PhoB	FNR	PhoP	FNR	CRP	PhoP	FNR	
fixC	b0043	CaiF	NarP	AllR	AllR	NtrC	NagC	AllR		
rplE	b3308	FNR	ArcA	Fis	FNR	ArcA	Fis	FNR	ArcA	Fis
rpsN	b3307	FNR	ArcA	Fis	FNR	ArcA	Fis	FNR	ArcA	Fis
rpsH	b3306	FNR	ArcA	Fis	FNR	ArcA	Fis	FNR	ArcA	Fis
rplF	b3305	FNR	ArcA	Fis	FNR	ArcA	Fis	FNR	ArcA	Fis
rplR	b3304	ArcA	FNR	Fis	FNR	ArcA	Fis	ArcA	FNR	Fis
rpsE	b3303	FNR	ArcA	Fis	FNR	ArcA	Fis	FNR	ArcA	Fis
rpmD	b3302	Fis	FNR	ArcA	FNR	ArcA	Fis	Fis	FNR	ArcA
rcsC	b2218	PhoB	PhoP	H-NS	PhoP	PurR	PhoB	PhoB	PhoP	
rplO	b3301	FNR	ArcA	Fis	FNR	ArcA	Fis	FNR	ArcA	Fis
prlA	b3300	FNR	ArcA	Fis	FNR	ArcA	CRP	FNR	ArcA	
yojN	b2216	CpxR	H-NS	FlhDC	IHF	CRP	FlhDC	FlhDC		
ompC	b2215	OmpR	Lrp	EnvY	Lrp	CpxR	OmpR	OmpR	Lrp	
phoQ	b1129	PhoP	FruR	MtlR	PhoP	Fis	PhoB	PhoP		
ada	b2213	Ada	UidR	UxuR	RcsAB	ArgR	UxuR	UxuR		
mqo	b2211	Fur	IHF	CRP	Fur	FNR	Fis	Fur		
potA	b1126	Fis	CRP	IHF	Fis	CRP	FNR	Fis	CRP	
yojH	b2210	CRP	FNR	Lrp	ArcA	CRP	IHF	CRP		
potB	b1125	Fis	CRP	IHF	CRP	FNR	Fis	Fis	CRP	
potC	b1124	Fis	CRP	IHF	FNR	CRP	Fis	Fis	CRP	
potD	b1123	FNR	CRP	Fis	CRP	ArcA	Fis	CRP	Fis	
caiB	b0038	CaiF	Rob	HU	AllR	NtrC	CaiF	CaiF		
caiF	b0034	NarL	H-NS	LexA	NarL	H-NS	FNR	NarL	H-NS	
carB	b0033	ArgR	PurR	IHF	ArgR	PurR	IHF	ArgR	PurR	IHF
carA	b0032	ArgR	PurR	IHF	ArgR	PurR	IHF	ArgR	PurR	IHF
eco	b2209	Lrp	CRP	FNR	Lrp	CRP	FNR	Lrp	CRP	FNR
napF	b2208	GntR	NarP	IscR	NarP	ModE	NarL	NarP		
napD	b2207	NarP	IscR	ModE	NarP	NarL	ModE	NarP	ModE	
napA	b2206	IscR	NarP	ModE	NarP	NarL	FlhDC	NarP		
napG	b2205	NarP	IscR	ModE	NarP	NarL	ModE	NarP	ModE	
napH	b2204	MtlR	MngR	NarP	NarP	ModE	NarL	NarP		
napB	b2203	NarP	IscR	ModE	NarP	NarL	ModE	NarP	ModE	
ycfW	b1118	Rob	SoxS	MarA	SoxS	NarL	PhoP	SoxS		
napC	b2202	Cbl	AscG	NarP	NarP	NarL	FlhDC	NarP		
ycfS	b1113	CpxR	BaeR	Fur	CpxR	CRP	rpoH	CpxR		
ycfR	b1112	IHF	CRP	FNR	CRP	IHF	FNR	IHF	CRP	FNR
ribF	b0025	ArgR	CpxR	GadE	FlhDC	ArgR	H-NS	ArgR		
rpsT	b0023	Fis	FNR	IHF	CRP	Fis	FNR	Fis	FNR	
insA_1	b0022	CpxR	H-NS	Fis	CRP	IHF	Fis	Fis		
ndh	b1109	ArcA	Fis	IHF	Fis	ArcA	FNR	ArcA	Fis	
ycfO	b1107	PhoB	CpxR	NarL	rpoH	CpxR	TyrR	CpxR		
nhaA	b0019	NhaR	H-NS	IHF	H-NS	Fis	FNR	H-NS		
fhuE	b1102	Fur	CRP	IHF	Fur	CRP	IHF	Fur	CRP	IHF
ptsG	b1101	DgsA	ArcA	CRP	DgsA	Fis	ArcA	DgsA	ArcA	
yi81_1	b0016	CRP	ArcA	IHF	CRP	IHF	FNR	CRP	IHF	

dnaJ	b0015	rpoH	CRP	Fis	rpoH	CRP	IHF	rpoH	CRP	
dnaK	b0014	rpoH	IHF	CRP	rpoH	CRP	IHF	rpoH	IHF	CRP
htgA	b0012	rpoH	Rob	SoxS	rpoH	IHF	FNR	rpoH		
thiC	b3994	CRP	IHF	FNR	CRP	Lrp	IHF	CRP	IHF	
thiE	b3993	CRP	Fis	IHF	Lrp	TrpR	CRP	CRP		
thiF	b3992	H-NS	CpxR	Lrp	Lrp	CRP	IHF	Lrp		
thiG	b3991	CRP	IHF	FNR	Lrp	TrpR	CRP	CRP		
talB	b0008	FNR	Fis	FruR	CRP	FNR	ArcA	FNR		
thrC	b0004	IHF	GadE	Lrp	IHF	Lrp	CRP	IHF	Lrp	
thrB	b0003	Lrp	CRP	IHF	Lrp	IHF	CRP	Lrp	CRP	IHF
thrA	b0002	Lrp	IHF	CRP	IHF	CRP	Lrp	Lrp	IHF	CRP
htrC	b3989	rpoH	PhoB	NtrC	rpoH	Rob	LexA	rpoH		
rpoC	b3988	ModE	FNR	FruR	Fis	FNR	ArgR	FNR		
rpoB	b3987	FNR	ArcA	Fis	Fis	FNR	ArcA	FNR	ArcA	Fis
rplJ	b3985	Fis	FNR	CRP	FNR	Fis	ArcA	Fis	FNR	
rplA	b3984	Fis	FNR	CRP	FNR	ArcA	Fis	Fis	FNR	
rplK	b3983	Fis	FNR	CRP	FNR	ArcA	Fis	Fis	FNR	
nusG	b3982	rpoH	H-NS	CpxR	CRP	Fis	H-NS	H-NS		
secE	b3981	ArgR	rpoH	Fis	Fis	ArgR	CRP	ArgR	Fis	
tufB	b3980	Fis	ArcA	FNR	Fis	FNR	ArcA	Fis	ArcA	FNR
b2896	b2896	SoxS	MarA	LexA	SoxS	NarL	CpxR	SoxS		
fldB	b2895	SoxS	NarL	rpoH	SoxS	CpxR	Nac	SoxS		
xerD	b2894	CpxR	GadE	H-NS	CpxR	rpoH	FlhDC	CpxR		
dsbC	b2893	MalT	CpxR	FadR	CpxR	FlhDC	rpoH	CpxR		
prfB	b2891	Fis	FNR	ArcA	Fis	CpxR	FNR	Fis	FNR	
lysS	b2890	FNR	CpxR	MarA	Fis	rpoH	CpxR	CpxR		
murB	b3972	H-NS	Fis	CRP	CpxR	H-NS	FlhDC	H-NS		
ygfU	b2888	GntR	NarL	GalR	RcsAB	AllR	GntR	GntR		
ygfQ	b2884	CusR	AllR	Cbl	AllR	RcsAB	GntR	AllR		
yeaN	b1791	FadR	RcsAB	CytR	UxuR	SoxS	RcsAB	RcsAB		
yeaM	b1790	FlhDC	CpxR	ModE	ArgR	LexA	FlhDC	FlhDC		
murI	b3967	rpoH	Fis	CRP	Lrp	Fis	CpxR	Fis		
btuB	b3966	PhoP	Fur	Fis	CpxR	rpoH	PhoP	PhoP		
trmA	b3965	rpoH	Fis	Nac	rpoH	Fis	CRP	rpoH	Fis	
udhA	b3962	CRP	IHF	ArcA	ArcA	CRP	FNR	CRP	ArcA	
oxyR	b3961	OxyR	H-NS	Fur	OxyR	FruR	SoxS	OxyR		
argH	b3960	ArgR	CRP	IHF	ArgR	PhoP	IHF	ArgR	IHF	
yeaL	b1789	ChbR	NagC	SoxS	PhoB	rpoH	NagC	NagC		
yeaH	b1784	NtrC	Fis	CRP	NtrC	Fis	CRP	NtrC	Fis	CRP
yeaG	b1783	NtrC	Fis	IHF	NtrC	Fis	CRP	NtrC	Fis	
mipA	b1782	FNR	ArcA	CRP	FNR	CRP	IHF	FNR	CRP	
yeaD	b1780	IHF	CRP	Fis	CRP	IHF	FNR	IHF	CRP	
argB	b3959	ArgR	CRP	IHF	ArgR	CRP	IHF	ArgR	CRP	IHF
argC	b3958	ArgR	CRP	IHF	ArgR	PhoP	CRP	ArgR	CRP	
argE	b3957	ArgR	CRP	PurR	ArgR	GadE	NtrC	ArgR		
ppc	b3956	Lrp	CRP	IHF	CRP	FNR	IHF	CRP	IHF	
pflD	b3951	AllR	GntR	LsrR	GntR	CsgD	AgaR	GntR		
b2866	b2866	GntR	FhlA	DeoR	AllR	AgaR	GntR	GntR		
gapA	b1779	FruR	rpoH	CRP	rpoH	FruR	CRP	FruR	rpoH	CRP
yeaA	b1778	Fur	FruR	ArcA	CRP	ArcA	FNR	ArcA		
b1777	b1777	Fur	FNR	CRP	CRP	ArcA	FNR	FNR	CRP	
b1776	b1776	NagC	GlpR	MetJ	NagC	FlhDC	CpxR	NagC		
pgm	b0688	HU	Lrp	GadE	HU	PhoP	SoxS	HU		
seqA	b0687	HU	PhoP	GadE	HU	PhoP	Lrp	HU	PhoP	
b1770	b1770	CpxR	FlhDC	Fis	CpxR	FlhDC	Fis	CpxR	FlhDC	Fis
fldA	b0684	SoxS	FNR	IHF	SoxS	FNR	CRP	SoxS	FNR	
fur	b0683	OxyR	SoxS	LexA	OxyR	SoxS	Fur	OxyR	SoxS	
ybfM	b0681	Lrp	RcsAB	CpxR	NagC	RcsAB	rpoH	RcsAB		
gldA	b3945	FNR	IHF	NarL	NarL	FlhDC	FruR	NarL		

katG	b3942	OxyR	FNR	GadE	OxyR	FNR	IHF	OxyR	FNR	
metF	b3941	MetJ	CRP	PhoP	MetJ	rpoH	CRP	MetJ	CRP	
metL	b3940	MetJ	PhoP	EnvY	MetJ	AllS	AllR	MetJ		
ansA	b1767	rpoH	PhoP	SoxS	PhoP	CRP	FNR	PhoP		
nagE	b0679	NagC	CRP	ArcA	NagC	CpxR	CRP	NagC	CRP	
topB	b1763	NtrC	H-NS	rpoH	PhoB	UxuR	rpoH	rpoH		
nagB	b0678	NagC	CRP	PhoP	NagC	NarL	PhoP	NagC	PhoP	
b1762	b1762	FruR	MarA	CpxR	RcsAB	CpxR	Fur	CpxR		
nagA	b0677	NagC	PhoP	CRP	NagC	PhoP	HU	NagC	PhoP	
gdhA	b1761	Nac	CRP	IHF	Nac	CRP	Lrp	Nac	CRP	
nagC	b0676	NagC	PhoP	SdiA	NagC	LexA	HU	NagC		
nagD	b0675	NagC	AppY	NarP	NagC	SoxS	PhoP	NagC		
asnB	b0674	GadX	FNR	CRP	GadX	ArgR	IHF	GadX		
metB	b3939	PhoP	MetJ	MetR	MetJ	PhoP	Fur	PhoP	MetJ	
metJ	b3938	Fur	MetR	rpoH	Fur	IHF	Lrp	Fur		
cytR	b3934	CytR	NanR	FlhDC	CytR	CRP	FNR	CytR		
hslV	b3932	rpoH	CRP	Fis	rpoH	CRP	IHF	rpoH	CRP	
hslU	b3931	rpoH	Fis	CRP	rpoH	CRP	IHF	rpoH	CRP	
menA	b3930	PhoB	Fis	NarL	PhoB	RcsAB	SoxS	PhoB		
yqeF	b2844	CRP	Fis	ArcA	Fis	Rob	FlhDC	Fis		
araE	b2841	AraC	Cbl	ModE	CRP	AraC	H-NS	AraC		
b1755	b1755	FruR	NarL	NtrC	UxuR	SoxS	NarL	NarL		
b1754	b1754	NarP	NarL	PhoB	ArgR	FlhDC	NarL	NarL		
ydjX	b1750	RcsAB	NarL	NikR	FNR	NarL	IHF	NarL		
yleA	b0661	LexA	MalT	rpoH	PurR	Fis	LexA	LexA		
glpF	b3927	GlpR	CRP	ArcA	CRP	GlpR	FNR	GlpR	CRP	
glpK	b3926	CRP	ArcA	FNR	CRP	FNR	IHF	CRP	FNR	
glpX	b3925	FlhDC	IHF	FNR	IHF	FNR	NarL	IHF	FNR	
fpr	b3924	MarA	SoxS	Fur	SoxS	MarA	CRP	MarA	SoxS	
lysA	b2838	FNR	CRP	Lrp	Lrp	MetJ	ArgR	Lrp		
yiiQ	b3920	SoxS	GadE	H-NS	ArgR	SoxS	CpxR	SoxS		
astC	b1748	ArgR	NtrC	CRP	ArgR	NtrC	Lrp	ArgR	NtrC	
astA	b1747	ArgR	NtrC	CRP	ArgR	NtrC	Lrp	ArgR	NtrC	
ygdP	b2830	H-NS	NarL	CpxR	FNR	CpxR	FlhDC	CpxR		
astD	b1746	ArgR	NtrC	FruR	ArgR	NtrC	Lrp	ArgR	NtrC	
astB	b1745	ArgR	NtrC	CRP	ArgR	NtrC	Lrp	ArgR	NtrC	
astE	b1744	ArgR	NtrC	PhoP	ArgR	NtrC	Lrp	ArgR	NtrC	
ybeY	b0659	rpoH	Fis	CRP	rpoH	PhoP	CRP	rpoH	CRP	
spy	b1743	BaeR	CpxR	CRP	BaeR	CpxR	CRP	BaeR	CpxR	CRP
ybeX	b0658	rpoH	Fis	CRP	rpoH	IHF	CRP	rpoH	CRP	
b1742	b1742	CpxR	FlhDC	LexA	FlhDC	CpxR	FNR	CpxR	FlhDC	
nadE	b1740	TyrR	CpxR	FruR	CpxR	CRP	IHF	CpxR		
gltJ	b0654	AraC	FlhDC	PhoB	FlhDC	ArgR	NtrC	FlhDC		
gltK	b0653	AraC	FlhDC	PhoB	FlhDC	NtrC	IHF	FlhDC		
gltL	b0652	FlhDC	rpoH	RbsR	FlhDC	NtrC	Lrp	FlhDC		
sbp	b3917	IHF	CRP	FNR	CysB	FlhDC	IHF	IHF		
pfkA	b3916	FruR	NarL	Rob	FruR	Fis	FNR	FruR		
b3914	b3914	CpxR	ArcA	CRP	CpxR	CRP	IHF	CpxR	CRP	
b3913	b3913	CpxR	CRP	Fis	CRP	CpxR	IHF	CpxR	CRP	
cpxR	b3912	CpxR	Fur	GadE	CpxR	SoxS	rpoH	CpxR		
thyA	b2827	CpxR	OmpR	rpoH	rpoH	CpxR	Fis	CpxR	rpoH	
ppdB	b2825	AgaR	GntR	Cbl	AgaR	GntR	NagC	AgaR	GntR	
osmE	b1739	Fis	SoxS	IHF	Fis	IHF	CRP	Fis	IHF	
celA	b1738	NagC	ChbR	AllS	NagC	Fis	IHF	NagC		
celB	b1737	NagC	ChbR	CusR	NagC	PurR	NarL	NagC		
celD	b1735	NagC	ChbR	EvgA	NagC	RcsAB	FlhDC	NagC		
celF	b1734	NagC	ChbR	CusR	NagC	RcsAB	CpxR	NagC		
ydjC	b1733	ChbR	NagC	SoxS	NagC	UxuR	LexA	NagC		
katE	b1732	NtrC	IHF	CRP	H-NS	CRP	IHF	IHF	CRP	

ybeL	b0643	H-NS	Fis	MarA	CRP	FNR	H-NS	H-NS		
leuS	b0642	NarL	rpoH	FNR	H-NS	PhoP	FNR	FNR		
sodA	b3908	Rob	MarA	SoxS	Rob	SoxS	MarA	Rob	MarA	SoxS
argA	b2818	ArgR	CRP	PurR	ArgR	CRP	IHF	ArgR	CRP	
b1729	b1729	IHF	FNR	CysB	IHF	H-NS	CRP	IHF		
b1728	b1728	LexA	CpxR	FlhDC	LexA	rpoH	CpxR	LexA	CpxR	
b1724	b1724	Fis	IHF	CRP	CRP	ArcA	H-NS	CRP		
b1722	b1722	FNR	IHF	CRP	CRP	IHF	FNR	FNR	IHF	CRP
b1720	b1720	LrhA	CpxR	FlhDC	CpxR	Fis	rpoH	CpxR		
mrdA	b0635	Fis	FNR	CRP	PurR	rpoH	Fis	Fis		
dacA	b0632	Fis	Lrp	FNR	Fis	CpxR	CRP	Fis		
ybeD	b0631	rpoH	TrpR	OxyR	rpoH	CRP	IHF	rpoH		
b2809	b2809	IHF	H-NS	FNR	IHF	ArcA	CRP	IHF		
fucR	b2805	FucR	AraC	DcuR	FucR	CpxR	CRP	FucR		
fucU	b2804	FucR	CRP	FNR	FucR	rpoH	CpxR	FucR		
fucK	b2803	FucR	CdaR	LsrR	FucR	CpxR	FlhDC	FucR		
fucI	b2802	FucR	FlhDC	CRP	FucR	FlhDC	NarL	FucR	FlhDC	
infC	b1718	FNR	Fis	CRP	FNR	CpxR	Fis	FNR	Fis	
fucP	b2801	FucR	RcsAB	GlpR	FucR	CRP	Fis	FucR		
rpmI	b1717	rpoH	FNR	Fis	FNR	IHF	CRP	FNR		
fucA	b2800	GntR	FucR	GalR	FucR	Fis	CRP	FucR		
rplT	b1716	rpoH	FNR	TyrR	FNR	IHF	ArcA	FNR		
pheS	b1714	ArcA	FNR	Fis	Fis	CRP	IHF	Fis		
crcB	b0624	OxyR	LexA	GntR	OxyR	CpxR	Lrp	OxyR		
crcA	b0622	PhoP	RcsAB	PhoB	PhoP	PhoB	ArgR	PhoP	PhoB	
nlpC	b1708	CpxR	PhoB	TrpR	Fis	CpxR	IHF	CpxR		
b1707	b1707	PhoB	CpxR	DicA	CpxR	PhoB	NarL	PhoB	CpxR	
b1706	b1706	IscR	Fur	CRP	IscR	SoxS	IHF	IscR		
aroH	b1704	TrpR	IHF	CRP	TrpR	FruR	FNR	TrpR		
citC	b0618	CsgD	LexA	AgaR	AgaR	NarL	Fur	AgaR		
ppsA	b1702	FruR	CRP	ArcA	FruR	CRP	Fis	FruR	CRP	
rna	b0611	rpoH	CpxR	PhoP	SoxS	Fis	CpxR	CpxR		
ybdR	b0608	RcsAB	GadW	FhlA	CpxR	Lrp	RcsAB	RcsAB		
ybdQ	b0607	rpoH	Fur	NarL	rpoH	CRP	FNR	rpoH		
ahpF	b0606	OxyR	MetJ	CRP	MetJ	OxyR	FNR	OxyR	MetJ	
ahpC	b0605	OxyR	MetJ	Fur	MetJ	OxyR	IHF	OxyR	MetJ	
ybdL	b0600	rpoH	NarL	CRP	rpoH	ArgR	PurR	rpoH		
creB	b4398	AraC	GntR	ModE	HyfR	BaeR	GntR	GntR		
creA	b4397	AraC	PhoB	NagC	SoxS	AlsR	PhoB	PhoB		
rob	b4396	MarA	Fur	IHF	MarA	NarL	CpxR	MarA		
trpR	b4393	TrpR	GalR	GalS	TrpR	ChbR	HyfR	TrpR		
deoD	b4384	ModE	CytR	DeoR	ModE	CytR	Fis	ModE	CytR	
deoB	b4383	ModE	CytR	DeoR	ModE	Fis	CytR	ModE	CytR	
rpmJ	b3299	Fis	FNR	ArgR	FNR	Fis	ArcA	Fis	FNR	
deoA	b4382	ModE	CytR	DeoR	ModE	CytR	Fis	ModE	CytR	
rpsM	b3298	FNR	ArcA	Fis	FNR	Fis	ArcA	FNR	ArcA	Fis
deoC	b4381	ModE	CytR	DeoR	ModE	CytR	Fis	ModE	CytR	
rpsK	b3297	FNR	ArcA	Fis	FNR	Fis	ArcA	FNR	ArcA	Fis
yjjI	b4380	LexA	NarL	ModE	NarL	FNR	IHF	NarL		
rpsD	b3296	FNR	ArcA	Fis	FNR	ArcA	Fis	FNR	ArcA	Fis
rpoA	b3295	FNR	ArcA	Fis	FNR	ArcA	Fis	FNR	ArcA	Fis
rplQ	b3294	FNR	Fis	ArcA	FNR	Fis	CRP	FNR	Fis	
yjjU	b4377	Lrp	NarL	IHF	H-NS	Lrp	CRP	Lrp		
osmY	b4376	Lrp	IHF	CRP	Lrp	IHF	CRP	Lrp	IHF	CRP
prfC	b4375	Fis	LexA	CRP	Fis	FNR	IHF	Fis		
yjjG	b4374	CpxR	NagC	PhoB	Fis	CpxR	PurR	CpxR		
rimI	b4373	Lrp	CpxR	LexA	rpoH	LexA	Fis	LexA		
rrmB	b3289	CpxR	rpoH	ArgR	CpxR	IHF	FNR	CpxR		
holD	b4372	Nac	LexA	Fis	Fis	LexA	CRP	LexA	Fis	

rsmC	b4371	CpxR	CRP	rpoH	Nac	LexA	CpxR	CpxR		
smg	b3284	CpxR	CRP	Fis	CpxR	CRP	H-NS	CpxR	CRP	
yrdD	b3283	H-NS	CpxR	CRP	CRP	FNR	H-NS	H-NS	CRP	
ccmC	b2199	NarP	Cbl	MngR	NarP	TyrR	NarL	NarP		
yrdC	b3282	PhoP	H-NS	CpxR	CpxR	FNR	IHF	CpxR		
ccmD	b2198	NarP	ModE	PspF	NarP	ModE	SoxS	NarP	ModE	
aroE	b3281	CpxR	AgaR	rpoH	CpxR	IHF	FNR	CpxR		
fhuF	b4367	Fur	FNR	IHF	Fur	IHF	CRP	Fur	IHF	
bglJ	b4366	Fur	CRP	Fis	Fur	IHF	H-NS	Fur		
rplY	b2185	rpoH	CRP	Fis	Fis	FNR	CRP	CRP	Fis	
holB	b1099	PurR	ArgR	Fis	Fis	LexA	FruR	Fis		
tmk	b1098	NhaR	FlhDC	CpxR	LexA	CpxR	Fis	CpxR		
fabF	b1095	FNR	CRP	Fis	Fis	CRP	ArcA	CRP	Fis	
fabG	b1093	Fis	Lrp	FNR	Fis	CRP	FNR	Fis	FNR	
fabD	b1092	GadE	CpxR	FNR	Fis	FNR	ArcA	FNR		
fabH	b1091	Fis	GadE	CpxR	Fis	ArcA	FNR	Fis		
plsX	b1090	Fis	CRP	FNR	Fis	FNR	ArcA	Fis	FNR	
yjiZ	b4356	CpxR	FlhDC	GadE	LexA	CpxR	Fur	CpxR		
tsr	b4355	CpxR	FlhDC	H-NS	CpxR	FlhDC	FNR	CpxR	FlhDC	
fis	b3261	Fis	IHF	CRP	Fis	IHF	CRP	Fis	IHF	CRP
yhdG	b3260	Fis	IHF	CRP	Fis	IHF	CRP	Fis	IHF	CRP
spr	b2175	H-NS	IHF	CRP	CRP	IHF	Lrp	IHF	CRP	
rpmF	b1089	Fis	SoxS	rpoH	FNR	Fis	IHF	Fis		
yceD	b1088	CpxR	FNR	rpoH	FNR	Fis	CRP	FNR		
b1085	b1085	FlhDC	CpxR	NagC	CpxR	FlhDC	ArgR	FlhDC	CpxR	
flgL	b1083	FlhDC	H-NS	CpxR	FlhDC	CpxR	H-NS	FlhDC	H-NS	CpxR
flgK	b1082	FlhDC	H-NS	FNR	FlhDC	H-NS	CpxR	FlhDC	H-NS	
flgJ	b1081	FlhDC	FNR	ArcA	FlhDC	H-NS	FNR	FlhDC	FNR	
flgI	b1080	FlhDC	FNR	ArcA	FlhDC	H-NS	FNR	FlhDC	FNR	
hsdM	b4349	RcsAB	SoxS	MarA	CpxR	ArgR	SoxS	SoxS		
hsdS	b4348	IHF	CRP	Fis	rpoH	IHF	Fis	IHF	Fis	
mcrB	b4346	rpoH	CpxR	Fis	CRP	CpxR	ArcA	CpxR		
mcrD	b4344	CsgD	NikR	GntR	NagC	GntR	FlhDC	GntR		
yjiT	b4342	H-NS	IHF	CpxR	FlhDC	H-NS	IHF	H-NS	IHF	
accC	b3256	FNR	Fis	ArcA	Fis	CRP	FNR	FNR	Fis	
accB	b3255	FNR	CRP	ArcA	Fis	CRP	FNR	FNR	CRP	
fruB	b2169	FruR	CRP	IHF	FruR	FNR	CRP	FruR	CRP	
fruK	b2168	FruR	IHF	FNR	FruR	CRP	FNR	FruR	FNR	
mreB	b3251	FlhDC	Fis	CRP	FlhDC	CpxR	Fis	FlhDC	Fis	
fruA	b2167	FruR	CRP	FNR	FruR	Fur	RcsAB	FruR		
mreC	b3250	FlhDC	Fis	FruR	FlhDC	LexA	rpoH	FlhDC		
flgH	b1079	FlhDC	FNR	CpxR	FlhDC	H-NS	FNR	FlhDC	FNR	
yeiL	b2163	MprA	CsgD	AraC	Rob	NagC	CsgD	CsgD		
flgG	b1078	FlhDC	FNR	ArcA	FlhDC	H-NS	FNR	FlhDC	FNR	
flgF	b1077	FlhDC	FNR	ArcA	FlhDC	H-NS	FNR	FlhDC	FNR	
flgE	b1076	FlhDC	H-NS	CpxR	FlhDC	H-NS	FNR	FlhDC	H-NS	
flgD	b1075	FlhDC	H-NS	CpxR	FlhDC	FNR	H-NS	FlhDC	H-NS	
flgC	b1074	FlhDC	CpxR	H-NS	FlhDC	H-NS	FNR	FlhDC	H-NS	
flgB	b1073	FlhDC	CpxR	H-NS	FlhDC	H-NS	FNR	FlhDC	H-NS	
flgA	b1072	FlhDC	CpxR	H-NS	FlhDC	FNR	H-NS	FlhDC	H-NS	
flgM	b1071	FlhDC	CpxR	H-NS	FlhDC	H-NS	FNR	FlhDC	H-NS	
flgN	b1070	FlhDC	CpxR	H-NS	FlhDC	H-NS	FNR	FlhDC	H-NS	
yjiL	b4334	Cbl	Rob	HU	Rob	ModE	GntR	Rob		
yhdE	b3248	FlhDC	NtrC	ModE	FlhDC	CpxR	AgaR	FlhDC		
rng	b3247	FlhDC	ArgR	LexA	FlhDC	ExuR	MarA	FlhDC		
nfo	b2159	Rob	MarA	SoxS	Rob	SoxS	MarA	Rob	MarA	SoxS
lysP	b2156	CRP	Fur	FNR	CRP	ArcA	Fis	CRP		
cirA	b2155	Fur	CRP	FNR	Fur	CRP	IHF	Fur	CRP	
yeiG	b2154	FruR	H-NS	Fis	TrpR	FlhDC	FruR	FruR		

folE	b2153	CRP	Fis	NtrC	FNR	CRP	IHF	CRP		
galS	b2151	GalR	GalS	CRP	FlhDC	GalR	GalS	GalR	GalS	
rimJ	b1066	PhoB	CpxR	Fur	FNR	CRP	CpxR	CpxR		
mglB	b2150	GalR	GalS	FlhDC	GalR	GalS	FlhDC	GalR	GalS	FlhDC
pyrC	b1062	PurR	IHF	ArgR	PurR	CRP	FNR	PurR		
dinI	b1061	LexA	SoxS	CpxR	LexA	CRP	ArcA	LexA		
yceP	b1060	rpoH	MarA	CaiF	rpoH	CRP	FNR	rpoH		
yjiD	b4326	Rob	FNR	Fur	FNR	IHF	Lrp	FNR		
yjiC	b4325	AgaR	TdcA	TdcR	AgaR	RcsAB	rpoH	AgaR		
uxuR	b4324	UxuR	ExuR	FruR	ExuR	BirA	MetR	ExuR		
uxuB	b4323	UxuR	GadE	GadX	ExuR	UxuR	CysB	UxuR		
uxuA	b4322	UxuR	ExuR	CRP	ExuR	UxuR	CRP	UxuR	ExuR	CRP
yhcN	b3238	IHF	CRP	FNR	CRP	IHF	ArcA	IHF	CRP	
gntP	b4321	UxuR	CRP	FlhDC	UxuR	AgaR	NagC	UxuR		
argR	b3237	ArgR	MelR	CytR	ArgR	rpoH	PhoP	ArgR		
fimH	b4320	Lrp	FlhDC	H-NS	Lrp	IHF	H-NS	Lrp	H-NS	
mdh	b3236	ArcA	CRP	FlhDC	FlhDC	ArcA	CRP	ArcA	CRP	FlhDC
mglA	b2149	GalR	GalS	FlhDC	FlhDC	GalR	GalS	GalR	GalS	FlhDC
mglC	b2148	GalR	GalS	FlhDC	FlhDC	Fis	GalR	GalR	FlhDC	
rplM	b3231	FNR	ArcA	Fis	FNR	ArcA	Fis	FNR	ArcA	Fis
rpsI	b3230	FNR	Fis	ArcA	FNR	ArcA	Fis	FNR	Fis	ArcA
solA	b1059	PhoB	CpxR	TrpR	CpxR	rpoH	RcsAB	CpxR		
cdd	b2143	CytR	CRP	Fis	CytR	CRP	FNR	CytR	CRP	
yceA	b1055	Fis	IHF	CRP	FNR	CRP	ArcA	CRP		
htrB	b1054	ArgR	PhoB	FruR	PurR	TyrR	PhoB	PhoB		
yceE	b1053	CytR	SoxS	CdaR	SoxS	Fis	MarA	SoxS		
msyB	b1051	NtrC	FNR	IHF	IHF	CRP	Fis	IHF		
fimG	b4319	Lrp	H-NS	FlhDC	Lrp	H-NS	IHF	Lrp	H-NS	
fimF	b4318	Lrp	H-NS	FlhDC	Lrp	H-NS	IHF	Lrp	H-NS	
fimD	b4317	H-NS	Lrp	IHF	Lrp	AgaR	H-NS	H-NS	Lrp	
fimC	b4316	Lrp	H-NS	IHF	Lrp	H-NS	IHF	Lrp	H-NS	IHF
fimI	b4315	H-NS	Lrp	IHF	Lrp	H-NS	IHF	H-NS	Lrp	IHF
fimA	b4314	Lrp	H-NS	IHF	Lrp	H-NS	IHF	Lrp	H-NS	IHF
fimB	b4312	H-NS	PhoB	MalT	H-NS	CRP	IHF	H-NS		
sspB	b3228	LexA	rpoH	CpxR	CpxR	LexA	FNR	LexA	CpxR	
yjhA	b4311	NagC	NanR	CdaR	NagC	NanR	GntR	NagC	NanR	
dcuD	b3227	PhoP	IHF	CRP	BaeR	PhoP	FlhDC	PhoP		
yjhT	b4310	NagC	NanR	CRP	NagC	NanR	CpxR	NagC	NanR	
nanR	b3226	NarL	SoxS	DicA	PhoP	NagC	SoxS	SoxS		
nanA	b3225	NanR	CRP	ArcA	NanR	CRP	FNR	NanR	CRP	
nanT	b3224	NanR	FlhDC	CRP	NanR	CRP	FNR	NanR	CRP	
nanE	b3223	NanR	NikR	TrpR	NanR	CRP	FNR	NanR		
nanK	b3222	NtrC	Zur	FlhDC	NanR	FlhDC	NarL	FlhDC		
yhcH	b3221	NanR	FlhDC	CRP	NanR	CRP	FNR	NanR	CRP	
yohF	b2137	MarA	H-NS	SoxS	SoxS	NtrC	RcsAB	SoxS		
yohD	b2136	FadR	Rob	SoxS	SoxS	ModE	FruR	SoxS		
dld	b2133	ArgR	PurR	FNR	FNR	CRP	ArcA	FNR		
mdoG	b1048	FNR	Fis	IHF	LexA	FNR	Fis	FNR	Fis	
yehZ	b2131	BaeR	RcsAB	OxyR	RcsAB	OxyR	NarL	RcsAB	OxyR	
csgA	b1042	CsgD	CpxR	GadX	CsgD	CpxR	ArgR	CsgD	CpxR	
csgB	b1041	CsgD	CpxR	OmpR	CsgD	CpxR	ArgR	CsgD	CpxR	
csgD	b1040	CsgD	RcsAB	OmpR	RcsAB	CsgD	CpxR	CsgD	RcsAB	
yjhS	b4309	CdaR	NagC	CysB	NagC	GntR	RcsAB	NagC		
yjhR	b4308	LexA	Fur	NarL	rpoH	NagC	LexA	LexA		
yjhQ	b4307	Cbl	MprA	Rob	Rob	TrpR	NagC	Rob		
sgcE	b4301	GntR	IdnR	RcsAB	GntR	AllS	CsgD	GntR		
sgcR	b4300	MarA	FadR	GntR	GntR	LexA	FlhDC	GntR		
gltF	b3214	Nac	GadE	ArgR	ArgR	Nac	Lrp	Nac	ArgR	
gltD	b3213	Nac	ArgR	GadE	Nac	ArgR	Lrp	Nac	ArgR	

gltB	b3212	Nac	GadE	ArgR	Nac	ArgR	Lrp	Nac	ArgR	
yehW	b2128	MprA	CsgD	GntR	AsnC	MprA	AllR	MprA		
yehV	b2127	OmpR	MarA	CpxR	NtrC	SoxS	CpxR	CpxR		
yehT	b2125	Fis	H-NS	CRP	IHF	CRP	FlhDC	CRP		
csgE	b1039	CsgD	RcsAB	OmpR	RcsAB	CsgD	CpxR	CsgD	RcsAB	
csgF	b1038	CsgD	RcsAB	OmpR	CsgD	RcsAB	CpxR	CsgD	RcsAB	
csgG	b1037	CsgD	RcsAB	OmpR	RcsAB	CsgD	CpxR	CsgD	RcsAB	
ycdX	b1034	MprA	PhoP	Cbl	FlhDC	FNR	PhoP	PhoP		
yrbL	b3207	PhoP	CpxR	rpoH	PhoP	CRP	IHF	PhoP		
ptsO	b3206	NarP	NarL	CpxR	CpxR	Lrp	NarL	NarL	CpxR	
yhbJ	b3205	rpoH	ModE	CRP	Lrp	FNR	CRP	CRP		
yehL	b2119	FhlA	HyfR	AtoC	HyfR	PhoB	CsgD	HyfR		
rpoN	b3202	rpoH	CRP	Fis	Lrp	FruR	Fis	Fis		
yhbG	b3201	rpoH	GntR	H-NS	CpxR	rpoH	FlhDC	rpoH		
yhbN	b3200	rpoH	Fis	ModE	rpoH	SoxS	CpxR	rpoH		
molR_2	b2116	PhoP	PdhR	RcsAB	NagC	RcsAB	LexA	RcsAB		
ycdT	b1025	NhaR	CpxR	H-NS	CpxR	LexA	PhoB	CpxR		
ycdP	b1021	NhaR	PhoB	AraC	rpoH	CpxR	PhoB	PhoB		
phoH	b1020	PhoB	CRP	Fis	PhoB	CRP	IHF	PhoB	CRP	
thiM	b2104	Fur	H-NS	CpxR	Lrp	H-NS	CpxR	H-NS	CpxR	
ycdO	b1018	Fur	CRP	FNR	CRP	IHF	FNR	CRP	FNR	
b1017	b1017	OmpR	Fur	GadE	Fur	CRP	IHF	Fur		
b2100	b2100	Rob	GntR	FhlA	UxuR	RcsAB	Rob	Rob		
putP	b1015	CRP	IHF	FNR	CRP	Lrp	IHF	CRP	IHF	
b1012	b1012	CsgD	PhoP	OmpR	PhoP	NtrC	ArcA	PhoP		
ycdG	b1006	PhoP	NtrC	ArcA	PhoP	NtrC	ArcA	PhoP	NtrC	ArcA
ycdF	b1005	Fis	FNR	H-NS	H-NS	CRP	IHF	H-NS		
wrbA	b1004	FNR	IHF	CRP	IHF	CRP	Fis	IHF	CRP	
agp	b1002	CRP	Fis	FNR	CRP	FNR	IHF	CRP	FNR	
cbpA	b1000	H-NS	FNR	ArcA	Lrp	H-NS	CRP	H-NS		
fdoG	b3894	FNR	CRP	IHF	IHF	CRP	Fis	CRP	IHF	
fdoI	b3892	FNR	IHF	CRP	IHF	FNR	CRP	FNR	IHF	CRP
exo	b2798	DhaR	GntR	IclR	GntR	NhaR	PhoP	GntR		
sdaB	b2797	H-NS	CRP	Lrp	Fis	IHF	CRP	CRP		
sdaC	b2796	ArcA	CRP	FNR	CRP	IHF	ArcA	ArcA	CRP	
ygdH	b2795	Lrp	CRP	FNR	PurR	FNR	CRP	CRP	FNR	
yihK	b3871	Fis	CRP	FNR	Fis	FNR	ArcA	Fis	FNR	
glnA	b3870	NtrC	Fis	CRP	NtrC	Fis	CRP	NtrC	Fis	CRP
barA	b2786	ModE	NarP	GadX	PaaX	CaiF	NarP	NarP		
relA	b2784	Rob	IscR	NarL	H-NS	CpxR	NarL	NarL		
ydiS	b1699	NagC	ChbR	CysB	UxuR	NagC	ExuR	NagC		
chpA	b2782	FruR	H-NS	CpxR	Fis	H-NS	CRP	H-NS		
mazG	b2781	Fis	rpoH	H-NS	Fis	H-NS	CpxR	Fis	H-NS	
pyrG	b2780	FNR	CRP	ArcA	Fis	CRP	FNR	FNR	CRP	
glnL	b3869	NtrC	Fis	CRP	NtrC	Fis	CRP	NtrC	Fis	CRP
glnG	b3868	NtrC	Fis	CRP	NtrC	AgaR	HyfR	NtrC		
hemN	b3867	H-NS	NtrC	CpxR	PhoB	ArgR	CpxR	CpxR		
yihI	b3866	PurR	PhoP	MetJ	Fur	CpxR	PurR	PurR		
polA	b3863	DnaA	H-NS	CpxR	PurR	CpxR	NarL	CpxR		
eno	b2779	FruR	FNR	ArcA	FruR	rpoH	Fis	FruR		
dsbA	b3860	CpxR	MarA	PurR	CpxR	IHF	FNR	CpxR		
ygcS	b2771	FhlA	NarL	PhoB	PhoB	NagC	EvgA	PhoB		
ydiJ	b1687	CRP	Fis	IHF	Lrp	IHF	H-NS	IHF		
b1685	b1685	CRP	GadE	H-NS	CRP	FNR	ArcA	CRP		
ydiC	b1684	OxyR	IscR	Fur	OxyR	Fur	IHF	OxyR	Fur	
ynhE	b1683	OxyR	IscR	Fur	OxyR	Fur	IscR	OxyR	IscR	Fur
cstA	b0598	CRP	ArcA	Fis	CRP	ArcA	FNR	CRP	ArcA	
ynhD	b1682	OxyR	IscR	Fur	OxyR	Fur	IHF	OxyR	Fur	
ybdB	b0597	Fur	CRP	OxyR	Fur	CRP	IHF	Fur	CRP	

sufD	b1681	OxyR	IscR	Fur	OxyR	Fur	IscR	OxyR	IscR	Fur
entA	b0596	Fur	CRP	IHF	Fur	CRP	IHF	Fur	CRP	IHF
sufS	b1680	OxyR	IscR	Fur	OxyR	Fur	IscR	OxyR	IscR	Fur
entB	b0595	Fur	CRP	IHF	Fur	CRP	IHF	Fur	CRP	IHF
entE	b0594	Fur	CRP	FNR	Fur	CRP	IHF	Fur	CRP	
entC	b0593	Fur	CRP	FNR	Fur	CRP	IHF	Fur	CRP	
fepB	b0592	Fur	CpxR	IscR	Fur	CRP	IHF	Fur		
ybdA	b0591	Fur	PhoP	GadE	Fur	CRP	FNR	Fur		
yihE	b3859	CpxR	H-NS	Fis	CpxR	H-NS	FNR	CpxR	H-NS	
cysJ	b2764	CysB	IHF	CRP	CysB	IHF	H-NS	CysB	IHF	
cysI	b2763	CysB	IHF	CRP	CysB	IHF	CRP	CysB	IHF	CRP
ynhA	b1679	OxyR	IscR	MarA	OxyR	Fur	IscR	OxyR	IscR	
cysH	b2762	CysB	IHF	CRP	CysB	IHF	CRP	CysB	IHF	CRP
ynhG	b1678	NtrC	IHF	CRP	Fis	CRP	IHF	IHF	CRP	
lpp	b1677	IHF	OxyR	CpxR	CRP	CpxR	FNR	CpxR		
b2760	b2760	GntR	FhlA	NagC	CpxR	GadE	GntR	GntR		
pykF	b1676	FruR	IHF	CRP	FruR	Fis	CRP	FruR	CRP	
b1674	b1674	CRP	IHF	NarL	NarL	FNR	IHF	IHF	NarL	
fepC	b0588	Fur	CpxR	NtrC	Fur	OxyR	FadR	Fur		
fepE	b0587	HU	Fur	NarL	PhoB	Fur	AllR	Fur		
entF	b0586	Fur	CRP	FNR	Fur	CRP	FNR	Fur	CRP	FNR
ydhU	b1670	GntR	FhlA	NagC	NagC	SoxS	Rob	NagC		
fes	b0585	Fur	CRP	FNR	Fur	CRP	FNR	Fur	CRP	FNR
fepA	b0584	Fur	CRP	IHF	Fur	CRP	IHF	Fur	CRP	IHF
entD	b0583	Fur	ExuR	CRP	Fur	CRP	IHF	Fur	CRP	
fadB	b3846	FadR	ArcA	CRP	FadR	ArcA	Fis	FadR	ArcA	
fadA	b3845	ArcA	FadR	Fis	FadR	Rob	Fis	FadR	Fis	
ubiB	b3844	MarA	TyrR	NarL	Fis	NarL	Lrp	NarL		
cysD	b2752	CysB	IHF	CRP	CysB	IHF	H-NS	CysB	IHF	
b1668	b1668	NtrC	H-NS	MarA	CpxR	NtrC	RcsAB	NtrC		
cysN	b2751	CysB	IHF	CRP	CysB	IHF	CRP	CysB	IHF	CRP
b1667	b1667	CpxR	Fis	ArcA	CRP	FNR	Fis	Fis		
cysC	b2750	CysB	IHF	Lrp	CysB	IHF	CRP	CysB	IHF	
b1664	b1664	CRP	IHF	FNR	CRP	FNR	IHF	CRP	IHF	FNR
ydhE	b1663	SoxS	Rob	NarL	CpxR	SoxS	Rob	SoxS	Rob	
nfnB	b0578	MarA	CpxR	Lrp	MarA	Fis	CRP	MarA		
cfa	b1661	FNR	CRP	H-NS	CRP	FNR	IHF	FNR	CRP	
ybdE	b0575	CusR	NagC	FNR	CusR	Lrp	CsgD	CusR		
ylcD	b0574	CusR	CRP	Fis	CusR	FNR	TrpR	CusR		
ylcC	b0573	CusR	IHF	CRP	CusR	FNR	CRP	CusR	CRP	
ylcB	b0572	CusR	CRP	IHF	CusR	FNR	CRP	CusR	CRP	
ybcZ	b0570	CusR	NagC	Fur	CusR	BirA	CsgD	CusR		
b3838	b3838	LexA	H-NS	Fis	Fis	ArgR	FNR	Fis		
yigN	b3832	FNR	CRP	H-NS	CRP	FNR	ArcA	FNR	CRP	
b2748	b2748	ArgR	NtrC	FlhDC	LexA	Fis	FlhDC	FlhDC		
udp	b3831	CytR	CRP	FNR	CytR	CRP	FNR	CytR	CRP	FNR
ysgA	b3830	FNR	CRP	Lrp	Lrp	IscR	FNR	FNR	Lrp	
surE	b2744	CusR	ArgR	MngR	GntR	PhoB	ArgR	ArgR		
pcm	b2743	RcsAB	H-NS	CpxR	AcrR	rpoH	RcsAB	RcsAB		
nlpD	b2742	FruR	CRP	H-NS	CRP	IHF	Fis	CRP		
rpoS	b2741	GadX	ArcA	CRP	GadX	ArcA	CRP	GadX	ArcA	CRP
sodB	b1656	Fur	H-NS	IHF	Fur	H-NS	IHF	Fur	H-NS	IHF
ydhO	b1655	H-NS	SoxS	IHF	SoxS	PurR	Fur	SoxS		
nfrB	b0569	Rob	FlhDC	NtrC	FlhDC	NarL	NtrC	FlhDC	NtrC	
ompT	b0565	CRP	IHF	FNR	CRP	IHF	H-NS	CRP	IHF	
appY	b0564	H-NS	NarL	NtrC	H-NS	CRP	FNR	H-NS		
ybcX	b0561	Fur	CpxR	FNR	Fur	CRP	FNR	Fur	FNR	
metE	b3829	MetJ	MetR	CRP	MetR	MetJ	CRP	MetJ	MetR	CRP
metR	b3828	MetJ	CsgD	GadW	MetR	MetJ	PspF	MetJ		

ygbL	b2738	FucR	NikR	GntR	GntR	AgaR	MtlR	GntR		
pldA	b3821	rpoH	MetJ	H-NS	ArgR	rpoH	FlhDC	rpoH		
ygbI	b2735	GutM	GutR	CaiF	HyfR	CueR	CaiF	CaiF		
b1648	b1648	Zur	NtrC	FlhDC	ArgR	FlhDC	rpoH	FlhDC		
fhlA	b2731	FhlA	NarL	SoxS	FhlA	AgaR	NarL	FhlA	NarL	
hypE	b2730	FhlA	YiaJ	LsrR	FhlA	NarL	IHF	FhlA		
sodC	b1646	NtrC	Fis	SoxS	Lrp	Fis	CRP	Fis		
b1645	b1645	CpxR	rpoH	PhoP	RcsAB	DgsA	rpoH	rpoH		
ybcU	b0557	PhoP	CRP	IHF	PhoP	CRP	IHF	PhoP	CRP	IHF
slyB	b1641	PhoP	Fis	NarL	PhoP	Fis	FNR	PhoP	Fis	
nmpC	b0553	OmpR	CRP	IHF	OmpR	IHF	CRP	OmpR	CRP	IHF
corA	b3816	PhoP	CRP	FNR	FruR	PhoP	FNR	PhoP	FNR	
uvrD	b3813	LexA	H-NS	FlhDC	LexA	TrpR	NagC	LexA		
hypD	b2729	FhlA	IHF	IscR	FhlA	IHF	FNR	FhlA	IHF	
hypC	b2728	FhlA	NarP	OxyR	FhlA	IHF	FNR	FhlA		
hypB	b2727	FhlA	IscR	NarP	FhlA	IHF	FNR	FhlA		
hypA	b2726	FhlA	NarP	IscR	FhlA	IHF	FNR	FhlA		
hycA	b2725	FhlA	PaaX	Zur	FhlA	ModE	IHF	FhlA		
hycC	b2723	FhlA	ModE	NarL	FhlA	ModE	IscR	FhlA	ModE	
hycE	b2721	FhlA	ModE	NarL	FhlA	ModE	IHF	FhlA	ModE	
tyrS	b1637	rpoH	FruR	H-NS	FruR	Fis	FNR	FruR		
hycF	b2720	FhlA	UlaR	RhaS	FhlA	ModE	IHF	FhlA		
gst	b1635	IHF	Fis	ArcA	CRP	IHF	ArcA	IHF	ArcA	
ydgR	b1634	OmpR	ArgR	Lrp	OmpR	FNR	CRP	OmpR		
b1631	b1631	DeoR	OxyR	rpoH	rpoH	Fis	NarP	rpoH		
ybcM	b0546	DicA	PhoB	MalT	CpxR	PhoB	rpoH	PhoB		
cyaA	b3806	CpxR	CRP	IscR	CRP	FNR	CpxR	CpxR	CRP	
aslB	b3800	Rob	Cbl	GntR	Rob	LexA	H-NS	Rob		
ascB	b2716	AscG	CytR	HyfR	HyfR	GntR	FhlA	HyfR		
hypF	b2712	FhlA	NarL	LsrR	HyfR	TyrR	FhlA	FhlA		
b1627	b1627	PurR	PhoB	CpxR	Fis	CpxR	FNR	CpxR		
b1626	b1626	NtrC	FNR	IHF	FNR	Fis	IHF	FNR	IHF	
add	b1623	Fis	FNR	CRP	CRP	FNR	IHF	FNR	CRP	
srlD	b2705	GutM	GutR	CRP	GutM	GutR	CRP	GutM	GutR	CRP
srlB	b2704	PhoB	GutM	GutR	GutM	GutR	CRP	GutM	GutR	
srlE	b2703	GutM	GutR	CRP	GutM	GutR	CRP	GutM	GutR	CRP
srlA	b2702	GutM	GutR	CRP	GutM	GutR	CRP	GutM	GutR	CRP
uidA	b1617	UidR	UxuR	CRP	UxuR	FlhDC	MarA	UxuR		
uidC	b1615	UidR	UxuR	CRP	UxuR	SoxS	Rob	UxuR		
ydgA	b1614	Fis	rpoH	FNR	PhoP	Fis	IHF	Fis		
folD	b0529	Fur	CpxR	CRP	ArgR	CpxR	Fis	CpxR		
manA	b1613	Rob	SoxS	MarA	rpoH	FruR	SoxS	SoxS		
fumA	b1612	ArcA	FNR	CRP	ArcA	FNR	CRP	ArcA	FNR	CRP
tus	b1610	Fis	CpxR	IHF	SoxS	FlhDC	CpxR	CpxR		
ppiB	b0525	Fis	CpxR	FNR	Fis	rpoH	CRP	Fis		
purE	b0523	PurR	PhoP	H-NS	PurR	PhoP	Lrp	PurR	PhoP	
purK	b0522	PurR	PhoP	CRP	PurR	Lrp	Nac	PurR		
rstB	b1609	PhoP	rpoH	H-NS	PhoP	FNR	Fis	PhoP		
rstA	b1608	PhoP	Lrp	IHF	PhoP	FNR	IHF	PhoP	IHF	
ydgB	b1606	PurR	FruR	NarL	SoxS	NarL	rpoH	NarL		
b1605	b1605	rpoH	MalT	FruR	PurR	PhoP	rpoH	rpoH		
b1604	b1604	CpxR	H-NS	FNR	CpxR	NtrC	PhoP	CpxR		
pntA	b1603	Lrp	OmpR	CRP	CRP	Lrp	IHF	Lrp	CRP	
fdrA	b0518	AgaR	MngR	AllR	AllR	PhoB	NarP	AllR		
pntB	b1602	OmpR	Lrp	CRP	CRP	Lrp	IHF	Lrp	CRP	
b1601	b1601	CpxR	FlhDC	RcsAB	SoxS	FlhDC	YiaJ	FlhDC		
ybbZ	b0514	AllR	DhaR	LexA	AllR	PhoB	HyfR	AllR		
ybbY	b0513	AllR	AtoC	PdhR	AllR	FlhDC	RcsAB	AllR		
ybbX	b0512	AllR	GntR	FucR	AllR	rpoH	IHF	AllR		

ybbW	b0511	AllR	NarL	FruR	AllR	ArgR	rpoH	AllR		
ybbQ	b0509	AllR	MngR	PdhR	AllR	PhoB	MprA	AllR		
hyi	b0508	AllR	Fis	FruR	AllR	NagC	EvgA	AllR		
gcl	b0507	AllR	FhlA	NarP	AllR	NagC	NtrC	AllR		
ybbU	b0506	AraC	Lrp	ArgR	Lrp	rpoH	H-NS	Lrp		
ybbS	b0504	AllR	TdcA	TdcR	AcrR	AllR	GntR	AllR		
fecI	b4293	Fur	CRP	FNR	Fur	CRP	IHF	Fur	CRP	
fecR	b4292	Fur	H-NS	CRP	Fur	CRP	IHF	Fur	CRP	
fecA	b4291	Fur	CRP	IHF	Fur	CRP	H-NS	Fur	CRP	
fecB	b4290	Fur	CRP	IHF	Fur	CRP	FNR	Fur	CRP	
fecC	b4289	Fur	CRP	IHF	Fur	CRP	H-NS	Fur	CRP	
fecD	b4288	Fur	PhoB	CysB	Fur	CRP	FNR	Fur		
fecE	b4287	Fur	CRP	FNR	Fur	CRP	FNR	Fur	CRP	FNR
yrbI	b3198	CpxR	H-NS	Fis	rpoH	CpxR	SoxS	CpxR		
yrbB	b3191	TyrR	IscR	rpoH	rpoH	LexA	PurR	rpoH		
yrbA	b3190	Nac	Fis	FruR	Fis	CRP	CpxR	Fis		
yjhB	b4279	CRP	Lrp	Fur	CRP	FNR	FlhDC	CRP		
yjgX	b4275	CpxR	Nac	Fis	CpxR	Fis	rpoH	CpxR	Fis	
murA	b3189	IHF	CRP	FNR	CpxR	Fis	FNR	FNR		
ispB	b3187	NagC	FruR	LexA	CpxR	ArgR	NagC	NagC		
rplU	b3186	FNR	Fis	ArcA	Fis	FNR	CRP	FNR	Fis	
rpmA	b3185	FNR	Fis	ArcA	FNR	Fis	IHF	FNR	Fis	
yhbE	b3184	PurR	Fis	IHF	Fis	CRP	ArcA	Fis		
yhbZ	b3183	Fis	IHF	FNR	Fis	CRP	ArcA	Fis		
greA	b3181	Fis	PhoP	FNR	Fis	IHF	FNR	Fis	FNR	
gatY	b2096	ArcA	CRP	IHF	ArcA	CRP	FNR	ArcA	CRP	
gatZ	b2095	ArcA	CRP	FNR	ArcA	CRP	FNR	ArcA	CRP	FNR
gatA	b2094	ArcA	CRP	FNR	ArcA	CRP	FNR	ArcA	CRP	FNR
gatB	b2093	ArcA	CRP	FNR	ArcA	CRP	FNR	ArcA	CRP	FNR
gatC	b2092	ArcA	CRP	FNR	ArcA	CRP	FNR	ArcA	CRP	FNR
gatD	b2091	ArcA	CRP	FNR	ArcA	CRP	FNR	ArcA	CRP	FNR
yjgB	b4269	SoxS	MarA	NarL	SoxS	MarA	OxyR	SoxS	MarA	
idnK	b4268	GntR	RcsAB	DgsA	GntR	IdnR	YiaJ	GntR		
idnD	b4267	GntR	IdnR	XylR	GntR	IdnR	NagC	GntR	IdnR	
idnO	b4266	GntR	CusR	RhaS	GntR	IdnR	NagC	GntR		
idnR	b4264	GntR	IdnR	DeoR	GntR	IdnR	FhlA	GntR	IdnR	
ftsJ	b3179	rpoH	IHF	CRP	rpoH	CpxR	CRP	rpoH	CRP	
hflB	b3178	rpoH	RcsAB	GadX	rpoH	CpxR	IHF	rpoH		
yjgP	b4261	CpxR	NanR	LexA	CpxR	Fis	ArgR	CpxR		
secG	b3175	Fis	ArgR	IHF	Fis	CRP	FNR	Fis		
argG	b3172	ArgR	CRP	IHF	ArgR	CRP	IHF	ArgR	CRP	IHF
yhbC	b3170	ArgR	Fis	CRP	ArgR	Fis	CRP	ArgR	Fis	CRP
yegQ	b2081	CRP	Fis	IHF	Fis	Fur	FNR	Fis		
b2080	b2080	H-NS	GadE	FNR	H-NS	CRP	IHF	H-NS		
valS	b4258	CpxR	H-NS	FNR	rpoH	CpxR	IHF	CpxR		
argI	b4254	ArgR	PhoP	CRP	ArgR	CRP	IHF	ArgR	CRP	
nusA	b3169	ArgR	Fis	CRP	ArgR	Fis	FNR	ArgR	Fis	
infB	b3168	ArgR	Fis	FNR	ArgR	Fis	CRP	ArgR	Fis	
rbfA	b3167	ArgR	ModE	Fis	ArgR	Fis	CRP	ArgR	Fis	
truB	b3166	ArgR	Fis	FNR	ArgR	Fis	CRP	ArgR	Fis	
rpsO	b3165	ArgR	Fis	CRP	ArgR	Fis	FNR	ArgR	Fis	
pnp	b3164	ArgR	Fis	CRP	ArgR	Fis	CRP	ArgR	Fis	CRP
nlpI	b3163	H-NS	FNR	IHF	Fis	CRP	FNR	FNR		
deaD	b3162	FNR	IHF	Fis	Fis	CRP	FNR	FNR	Fis	
yegB	b2077	BaeR	FhlA	NikR	NagC	BaeR	CpxR	BaeR		
yhbW	b3160	TrpR	IHF	FlhDC	SoxS	MarA	IHF	IHF		
b2074	b2074	BaeR	CpxR	PhoB	BaeR	CpxR	FlhDC	BaeR	CpxR	
b2072	b2072	Cbl	Rob	GntR	NagC	TrpR	Rob	Rob		
yjgH	b4248	Cbl	PhoB	GadW	PhoB	RcsAB	ArcA	PhoB		

pyrB	b4245	IHF	FNR	ArgR	IHF	PurR	FNR	IHF	FNR	
pyrI	b4244	PurR	CRP	IHF	IHF	FNR	PurR	PurR	IHF	
yjgF	b4243	CRP	FNR	IHF	FNR	IHF	CRP	CRP	FNR	IHF
mgtA	b4242	PhoP	CRP	FNR	PhoP	CRP	FNR	PhoP	CRP	FNR
treR	b4241	PhoP	HcaR	PhoB	PhoP	DeoR	NsrR	PhoP		
treB	b4240	ArcA	CRP	Fis	ArcA	CRP	IHF	ArcA	CRP	
yhbO	b3153	NtrC	MarA	SoxS	SoxS	Lrp	MarA	MarA	SoxS	
yegD	b2069	FadR	Fis	FNR	PurR	FadR	PhoB	FadR		
yraP	b3150	FlhDC	CpxR	RcsAB	CpxR	Fis	Fur	CpxR		
udk	b2066	SoxS	FNR	Fis	FNR	PurR	SoxS	SoxS	FNR	
wza	b2062	Cbl	RcsAB	AllR	RcsAB	FlhDC	CpxR	RcsAB		
treC	b4239	ArcA	CRP	IHF	ArcA	CRP	IHF	ArcA	CRP	IHF
nrdD	b4238	FNR	Fis	NtrC	FNR	CRP	IHF	FNR		
yjfG	b4233	LexA	CRP	NarL	AgaR	SoxS	LexA	LexA		
yraO	b3149	CpxR	MarA	ArgR	CpxR	PhoP	H-NS	CpxR		
ytfT	b4230	FlhDC	GntR	Fur	GntR	NagC	PhoB	GntR		
wcaB	b2058	RcsAB	HyfR	AgaR	RcsAB	FlhDC	rpoH	RcsAB		
agaI	b3141	AgaR	Cbl	CusR	AgaR	RcsAB	NagC	AgaR		
agaD	b3140	AgaR	Cbl	CsgD	AgaR	YiaJ	NagC	AgaR		
ytfR	b4228	CRP	Fis	NarL	PhoB	NtrC	Fis	Fis		
ytfQ	b4227	CRP	Fis	FNR	CRP	Fis	IHF	CRP	Fis	
ppa	b4226	Fis	CRP	CpxR	Fis	CRP	FNR	Fis	CRP	
ytfN	b4221	rpoH	NagC	CpxR	CpxR	PhoP	ArgR	CpxR		
agaS	b3136	AgaR	TdcA	TdcR	AgaR	NagC	Rob	AgaR		
agaA	b3135	AgaR	RcsAB	FhlA	AgaR	AllR	EvgA	AgaR		
agaW	b3134	AgaR	NtrC	NarP	EvgA	AgaR	DeoR	AgaR		
agaV	b3133	AgaR	TrpR	PhoP	AgaR	Rob	NagC	AgaR		
agaZ	b3132	AgaR	ExuR	AraC	AgaR	HyfR	CdaR	AgaR		
agaR	b3131	AgaR	RbsR	ExuR	AgaR	CpxR	FlhDC	AgaR		
yhaV	b3130	OxyR	PhoP	AgaR	CpxR	PhoP	CRP	PhoP		
galF	b2042	H-NS	Fis	LexA	LexA	Lrp	CRP	LexA		
rfbB	b2041	H-NS	Fis	FNR	IHF	CRP	FNR	FNR		
rfbD	b2040	Fis	SoxS	IHF	rpoH	CRP	IHF	IHF		
ytfK	b4217	H-NS	OmpR	FNR	CRP	FNR	ArcA	FNR		
sohA	b3129	Fis	CpxR	IHF	Fis	FNR	CRP	Fis		
yhaG	b3128	CdaR	FNR	CRP	CdaR	FNR	CRP	CdaR	FNR	CRP
yhaU	b3127	CdaR	CRP	FNR	CdaR	FNR	CRP	CdaR	CRP	FNR
yhaF	b3126	CdaR	FNR	CRP	CdaR	FNR	CRP	CdaR	FNR	CRP
yhaE	b3125	CdaR	FNR	CRP	CdaR	FNR	CRP	CdaR	FNR	CRP
yhaD	b3124	CdaR	FNR	CRP	CdaR	FNR	FlhDC	CdaR	FNR	
rfbA	b2039	FNR	H-NS	CRP	IHF	CRP	FNR	FNR	CRP	
rplI	b4203	FNR	CpxR	ArcA	Fis	FNR	CRP	FNR		
rpsR	b4202	FNR	ArcA	Fis	FNR	ArcA	Fis	FNR	ArcA	Fis
priB	b4201	FNR	ArcA	Fis	FNR	ArcA	Fis	FNR	ArcA	Fis
tdcB	b3117	TdcA	TdcR	CpxR	TdcA	TdcR	IHF	TdcA	TdcR	
rpsF	b4200	FNR	Fis	ArcA	FNR	ArcA	Fis	FNR	Fis	ArcA
gnd	b2029	GadE	Fis	IHF	GadE	rpoH	Lrp	GadE		
wzzB	b2027	CRP	IHF	H-NS	Fis	CRP	FNR	CRP		
hisI	b2026	IHF	CRP	Lrp	TrpR	Lrp	CRP	CRP	Lrp	
hisF	b2025	Lrp	IHF	ArgR	IHF	CRP	Lrp	Lrp	IHF	
hisA	b2024	ArgR	IHF	Lrp	Nac	IHF	Lrp	IHF	Lrp	
hisH	b2023	IHF	Lrp	ArgR	IHF	CRP	FNR	IHF		
hisB	b2022	IHF	CRP	Lrp	CRP	IHF	FNR	IHF	CRP	
hisC	b2021	IHF	CRP	ArgR	CRP	IHF	FNR	IHF	CRP	
hisD	b2020	IHF	CRP	FNR	IHF	CRP	FNR	IHF	CRP	FNR
hisG	b2019	CRP	IHF	Lrp	CRP	IHF	FNR	CRP	IHF	
b2016	b2016	Fur	CpxR	OxyR	Fis	CpxR	PhoB	CpxR		
yeeY	b2015	Fur	CpxR	H-NS	rpoH	CpxR	FruR	CpxR		
yeeF	b2014	FNR	CRP	IHF	CRP	Fis	ArcA	CRP		

yeeE	b2013	CysB	IHF	CRP	IHF	CRP	H-NS	IHF	CRP	
yeeD	b2012	CysB	IHF	CRP	IHF	CRP	ArcA	IHF	CRP	
sbmC	b2009	CRP	FNR	ArcA	CRP	ArcA	FNR	CRP	FNR	ArcA
yeeA	b2008	NanR	LexA	LrhA	LexA	CRP	H-NS	LexA		
yeeX	b2007	Fis	rpoH	PhoB	Fis	CRP	ArcA	Fis		
b2001	b2001	OxyR	GadE	GadX	OxyR	NarL	ArcA	OxyR		
flu	b2000	OxyR	FNR	FruR	OxyR	NarL	ArcA	OxyR		
wecF	b3793	FlhDC	NtrC	rpoH	FlhDC	ArgR	NtrC	FlhDC	NtrC	
rffG	b3788	Lrp	Fis	CpxR	Fis	FlhDC	CpxR	Fis	CpxR	
wecC	b3787	Lrp	CpxR	FlhDC	rpoH	H-NS	CpxR	CpxR		
rfe	b3784	CytR	H-NS	NhaR	Fis	FlhDC	H-NS	H-NS		
rho	b3783	ArcA	CpxR	CRP	Fis	CRP	CpxR	CpxR	CRP	
recA	b2699	LexA	ArcA	IHF	LexA	CRP	ArcA	LexA	ArcA	
oraA	b2698	LexA	CpxR	IHF	LexA	CRP	ArcA	LexA		
alaS	b2697	FNR	GadE	FruR	Fis	IHF	FNR	FNR		
rhlB	b3780	RcsAB	LexA	CpxR	CpxR	ArgR	FNR	CpxR		
ilvC	b3774	CRP	Lrp	IHF	CRP	IHF	Lrp	CRP	Lrp	IHF
ilvA	b3772	Lrp	IHF	Fur	Lrp	IHF	FNR	Lrp	IHF	
gshA	b2688	FruR	H-NS	CpxR	H-NS	Fis	FNR	H-NS		
ilvD	b3771	Lrp	IHF	CRP	Lrp	IHF	FNR	Lrp	IHF	
ygaG	b2687	OxyR	Fis	NarL	FruR	CpxR	Fis	Fis		
ilvE	b3770	Lrp	IHF	Fur	Lrp	IHF	FNR	Lrp	IHF	
emrR	b2684	FNR	FruR	IHF	MprA	CRP	FNR	FNR		
b1599	b1599	NtrC	H-NS	Fis	Fis	IHF	CRP	Fis		
b1598	b1598	PhoP	CpxR	PhoB	PhoB	SoxS	ArgR	PhoB		
asr	b1597	PhoB	CRP	ArcA	PhoB	CRP	IHF	PhoB	CRP	
mlc	b1594	DgsA	TorR	AraC	DgsA	CRP	IHF	DgsA		
b1593	b1593	NarL	IscR	NikR	FNR	NarL	IHF	NarL		
b1592	b1592	NarL	RcsAB	FhlA	SoxS	RcsAB	FlhDC	RcsAB		
b1590	b1590	FNR	NikR	Zur	FNR	NarL	IHF	FNR		
ilvM	b3769	Lrp	IHF	Fur	Lrp	IHF	CRP	Lrp	IHF	
ilvG_1	b3767	Lrp	IHF	CRP	Lrp	IHF	CRP	Lrp	IHF	CRP
ilvL	b3766	CusR	Lrp	LexA	Lrp	IHF	CRP	Lrp		
proX	b2679	H-NS	FNR	ArcA	H-NS	CRP	IHF	H-NS		
proW	b2678	H-NS	CRP	ArcA	H-NS	CRP	Fis	H-NS	CRP	
proV	b2677	H-NS	Fis	CRP	H-NS	CRP	ArcA	H-NS	CRP	
nrdF	b2676	Fur	IHF	CRP	Fur	CRP	IHF	Fur	IHF	CRP
nrdE	b2675	Fur	CRP	FNR	Fur	CRP	IHF	Fur	CRP	
nrdI	b2674	Fur	FNR	IHF	Fur	CRP	IHF	Fur	IHF	
nrdH	b2673	Fur	FNR	IHF	Fur	CRP	IHF	Fur	IHF	
b1589	b1589	NarL	NarP	IscR	FNR	NarL	IHF	NarL		
ygaM	b2672	IHF	H-NS	Lrp	CRP	IHF	Fis	IHF		
b1588	b1588	FNR	Fur	NarL	FNR	NarL	IHF	FNR	NarL	
ygaC	b2671	Fur	CpxR	IHF	Fur	RcsAB	NagC	Fur		
b2670	b2670	CpxR	Fur	IHF	IHF	FNR	CRP	IHF		
b1586	b1586	OmpR	PhoB	NtrC	NtrC	Fis	PhoB	PhoB	NtrC	
b0499	b0499	FlhDC	CpxR	rpoH	PhoB	CpxR	NarL	CpxR		
b1583	b1583	FruR	FNR	CRP	PhoP	FNR	CRP	FNR	CRP	
ybbN	b0492	rpoH	GadX	Rob	rpoH	CRP	IHF	rpoH		
yieO	b3754	RcsAB	rpoH	MarA	rpoH	FruR	CpxR	rpoH		
rbsB	b3751	RbsR	CRP	FNR	CRP	FNR	IHF	CRP	FNR	
rbsC	b3750	RbsR	IHF	FNR	CRP	RbsR	FNR	RbsR	FNR	
gabP	b2663	Nac	GadE	NhaR	Nac	Lrp	H-NS	Nac		
b1579	b1579	DicA	Fur	CpxR	RcsAB	LexA	CpxR	CpxR		
gabT	b2662	Nac	H-NS	Lrp	Nac	Lrp	H-NS	Nac	H-NS	Lrp
gabD	b2661	Nac	Lrp	H-NS	Nac	Lrp	H-NS	Nac	Lrp	H-NS
ygaF	b2660	Lrp	NtrC	H-NS	Lrp	H-NS	CRP	Lrp	H-NS	
ybaT	b0486	GadX	MarA	H-NS	GadX	Lrp	H-NS	GadX	H-NS	
ybaS	b0485	GadX	H-NS	MarA	GadX	H-NS	ArcA	GadX	H-NS	

ybaQ	b0483	CpxR	Fur	LexA	CpxR	CRP	IHF	CpxR		
ybaP	b0482	Fur	SoxS	LexA	SoxS	Cbl	CpxR	SoxS		
ushA	b0480	FadR	CueR	FNR	FadR	PurR	rpoH	FadR		
rbsA	b3749	RbsR	CRP	Fis	CRP	RbsR	ArcA	RbsR	CRP	
asnC	b3743	Nac	AsnC	PhoB	Nac	AsnC	CpxR	Nac	AsnC	
b2659	b2659	Lrp	CsiR	H-NS	Lrp	H-NS	CRP	Lrp	H-NS	
mioC	b3742	Nac	AsnC	CRP	Nac	AsnC	CRP	Nac	AsnC	CRP
gidA	b3741	Nac	AsnC	Fis	Nac	AsnC	Fis	Nac	AsnC	Fis
flxA	b1566	FlhDC	H-NS	GadE	FlhDC	CpxR	H-NS	FlhDC	H-NS	
gsk	b0477	PurR	rpoH	H-NS	rpoH	Fis	SoxS	rpoH		
hemH	b0475	OxyR	MetR	IclR	OxyR	MetJ	rpoH	OxyR		
adk	b0474	CRP	FNR	Fis	CRP	FNR	IHF	CRP	FNR	
htpG	b0473	rpoH	CRP	IHF	rpoH	CRP	IHF	rpoH	CRP	IHF
recR	b0472	CytR	DeoR	CpxR	SoxS	CpxR	Fis	CpxR		
dnaX	b0470	Fis	GadE	FNR	Fis	CpxR	SoxS	Fis		
atpI	b3739	FlhDC	CpxR	Fis	CRP	FNR	CpxR	CpxR		
atpB	b3738	FNR	Fis	CRP	FNR	CRP	IHF	FNR	CRP	
atpF	b3736	FNR	Fis	ArcA	IHF	FNR	Fis	FNR	Fis	
atpH	b3735	CRP	IHF	FNR	FNR	Fis	CRP	CRP	FNR	
atpA	b3734	FNR	ArcA	CRP	FNR	IHF	CRP	FNR	CRP	
atpG	b3733	FNR	ArcA	Fis	FNR	Fis	IHF	FNR	Fis	
atpD	b3732	FNR	Fis	ArcA	FNR	CRP	IHF	FNR		
glmU	b3730	NagC	CpxR	CRP	NagC	FNR	IHF	NagC		
b1556	b1556	GntR	NarL	FlhDC	FlhDC	CpxR	NagC	FlhDC		
apt	b0469	FNR	Fis	ArcA	Fis	CRP	IHF	Fis		
priC	b0467	ZraR	MtlR	Cbl	Cbl	LsrR	AgaR	Cbl		
acrB	b0462	Rob	MarA	SoxS	Rob	SoxS	PhoP	Rob	SoxS	
ybaJ	b0461	CpxR	FadR	Fis	CpxR	FNR	CRP	CpxR		
hha	b0460	CpxR	Fur	PhoB	CpxR	FNR	CRP	CpxR		
glmS	b3729	NagC	CRP	Fis	NagC	FNR	CRP	NagC	CRP	
pstS	b3728	PhoB	IHF	FNR	PhoB	IHF	FNR	PhoB	IHF	FNR
pstC	b3727	PhoB	IHF	FNR	PhoB	IHF	FNR	PhoB	IHF	FNR
pstA	b3726	PhoB	IHF	FNR	PhoB	IHF	FNR	PhoB	IHF	FNR
pstB	b3725	PhoB	IHF	FNR	PhoB	IHF	FNR	PhoB	IHF	FNR
phoU	b3724	PhoB	UxuR	HcaR	PhoB	IHF	FNR	PhoB		
ydfM	b1546	Fur	H-NS	CpxR	Fur	CRP	FNR	Fur		
ylaC	b0458	IHF	CRP	ArcA	CRP	ArcA	FNR	CRP	ArcA	
ylaB	b0457	CytR	CpxR	H-NS	PhoB	CpxR	NtrC	CpxR		
b1541	b1541	NarL	IHF	ArcA	FNR	ArcA	CRP	ArcA		
ybaY	b0453	IHF	CRP	Fis	Lrp	IHF	Fis	IHF	Fis	
tesB	b0452	HU	PhoB	HcaR	CueR	PhoB	Cbl	PhoB		
glnK	b0450	NtrC	GadX	IHF	NtrC	GadX	Nac	NtrC	GadX	
yieE	b3712	CpxR	H-NS	FNR	CpxR	CRP	rpoH	CpxR		
ydeH	b1535	CpxR	LrhA	NhaR	CpxR	CRP	FNR	CpxR		
marA	b1531	Rob	MarA	SoxS	Rob	SoxS	MarA	Rob	MarA	SoxS
marR	b1530	Rob	MarA	SoxS	Rob	SoxS	MarA	Rob	MarA	SoxS
ybaU	b0441	rpoH	CpxR	HU	rpoH	CpxR	RcsAB	rpoH	CpxR	
tnaB	b3709	TorR	FlhDC	H-NS	TorR	CRP	ArcA	TorR		
tnaA	b3708	TorR	CRP	ArcA	TorR	CRP	IHF	TorR	CRP	
tnaL	b3707	TorR	CRP	Fis	TorR	CRP	IHF	TorR	CRP	
thdF	b3706	rpoH	GntR	NarL	rpoH	NagC	PhoB	rpoH		
rnpA	b3704	Fis	FNR	CRP	Fis	CRP	FNR	Fis	FNR	CRP
rpmH	b3703	LexA	FNR	Fis	Fis	FNR	CRP	FNR	Fis	
dnaA	b3702	DnaA	Fis	LexA	rpoH	Fis	LexA	Fis	LexA	
dnaN	b3701	DnaA	LexA	CpxR	Fis	DnaA	CRP	DnaA		
recN	b2616	LexA	IHF	ArcA	LexA	CRP	ArcA	LexA	ArcA	
grpE	b2614	rpoH	CRP	Fis	rpoH	CRP	IHF	rpoH	CRP	
ydeA	b1528	ArcA	CRP	FNR	ArcA	Fis	FNR	ArcA	FNR	
ffh	b2610	rpoH	FNR	Fis	LexA	Fis	CpxR	Fis		

yneH	b1524	PhoB	IclR	MarA	SoxS	PhoP	MarA	MarA		
lon	b0439	rpoH	GadX	CRP	rpoH	GadX	CRP	rpoH	GadX	CRP
clpX	b0438	rpoH	Fur	IHF	rpoH	CRP	CpxR	rpoH		
clpP	b0437	rpoH	CRP	IHF	rpoH	IHF	CRP	rpoH	CRP	IHF
tig	b0436	FNR	Fis	ArcA	Fis	FNR	CpxR	FNR	Fis	
bolA	b0435	OmpR	H-NS	Fis	OmpR	H-NS	CRP	OmpR	H-NS	
yajG	b0434	Fis	CRP	FNR	Fis	CRP	CpxR	Fis	CRP	
cyoA	b0432	GadE	Fur	ArcA	Fur	GadE	ArcA	GadE	Fur	ArcA
cyoB	b0431	GadE	ArcA	Fur	Fur	GadE	ArcA	GadE	ArcA	Fur
cyoC	b0430	GadE	Fur	ArcA	Fur	GadE	ArcA	GadE	Fur	ArcA
rpsP	b2609	FNR	ArcA	Fis	FNR	ArcA	Fis	FNR	ArcA	Fis
yfjA	b2608	FNR	Fis	ArcA	FNR	ArcA	Fis	FNR	Fis	ArcA
trmD	b2607	FNR	ArcA	Fis	FNR	ArcA	Fis	FNR	ArcA	Fis
rplS	b2606	FNR	Fis	ArcA	FNR	ArcA	Fis	FNR	Fis	ArcA
yfiN	b2604	CpxR	rpoH	Fur	CpxR	FNR	rpoH	CpxR	rpoH	
b2603	b2603	PhoP	CpxR	H-NS	CpxR	FNR	CRP	CpxR		
tam	b1519	NtrC	FNR	CRP	NtrC	Lrp	RcsAB	NtrC		
b1518	b1518	LsrR	CRP	FNR	CRP	ArcA	FNR	CRP	FNR	
aroF	b2601	TyrR	TrpR	IHF	TyrR	CRP	ArcA	TyrR		
yneB	b1517	LsrR	CRP	FNR	CRP	IHF	Fis	CRP		
tyrA	b2600	TyrR	TrpR	IHF	TyrR	CRP	H-NS	TyrR		
b1516	b1516	CRP	LsrR	FNR	CRP	ArcA	FNR	CRP	FNR	
cyoD	b0429	GadE	Fur	ArcA	Fur	GadE	ArcA	GadE	Fur	ArcA
b1513	b1513	CRP	LsrR	FNR	NagC	CRP	FruR	CRP		
cyoE	b0428	GadE	Fur	ArcA	Fur	GadE	ArcA	GadE	Fur	ArcA
ydeW	b1512	CRP	Fis	ArcA	CRP	FNR	IHF	CRP		
ydeV	b1511	CRP	FNR	Fur	PhoB	CRP	Fis	CRP		
apbA	b0425	CpxR	Fur	MarA	PhoP	CpxR	rpoH	CpxR		
yajK	b0423	PurR	Fis	rpoH	Fis	CpxR	IHF	Fis		
xseB	b0422	FlhDC	H-NS	PhoB	CpxR	H-NS	CRP	H-NS		
ispA	b0421	FlhDC	CRP	Fis	H-NS	Fis	CpxR	Fis		
b1505	b1505	CRP	FNR	IHF	CpxR	Fur	IHF	IHF		
b1502	b1502	PhoB	FlhDC	CpxR	CpxR	RcsAB	rpoH	CpxR		
nusB	b0416	ArgR	LexA	rpoH	IHF	rpoH	Fur	rpoH		
ribD	b0414	LexA	FruR	Fis	CpxR	Fis	FNR	Fis		
tsx	b0411	CytR	DeoR	CRP	CytR	CRP	ArcA	CytR	CRP	
secD	b0408	Fis	CRP	FNR	Fis	CRP	H-NS	Fis	CRP	
yajC	b0407	Fis	PhoP	PurR	Fis	CRP	CpxR	Fis		
tgt	b0406	FNR	Fis	CRP	Fis	FNR	CRP	FNR	Fis	CRP
queA	b0405	Fis	CRP	FNR	Fis	IHF	ArcA	Fis		
phoR	b0400	PhoB	MalT	DhaR	PhoB	Fur	NarL	PhoB		
ptxA	b4195	UlaR	HyfR	IdnR	AllS	IdnR	FucR	IdnR		
yjfP	b4190	CsgD	AraC	CaiF	MarR	MprA	CsgD	CsgD		
yjfO	b4189	Fis	CRP	FNR	CRP	FNR	H-NS	CRP	FNR	
aidB	b4187	Ada	MarA	Lrp	Lrp	Ada	H-NS	Ada	Lrp	
yqjE	b3099	MarA	Fur	Fis	IHF	Lrp	Fis	Fis		
yqjB	b3096	CpxR	NarP	ModE	CpxR	ArgR	Fis	CpxR		
yqjA	b3095	CpxR	rpoH	Fis	CpxR	SoxS	TrpR	CpxR		
exuR	b3094	ExuR	rpoH	H-NS	ExuR	CpxR	SoxS	ExuR		
exuT	b3093	ExuR	GadX	NarL	ExuR	FadR	TrpR	ExuR		
uxaC	b3092	ExuR	CRP	ArcA	ExuR	FNR	UxuR	ExuR		
uxaA	b3091	FlhDC	ExuR	FNR	ExuR	AgaR	MngR	ExuR		
vacB	b4179	H-NS	CpxR	ArcA	CpxR	AgaR	TyrR	CpxR		
purA	b4177	MarA	GadE	PurR	MarA	PurR	GadE	MarA	GadE	PurR
hflC	b4175	ModE	CpxR	Fis	rpoH	SoxS	CpxR	CpxR		
hflK	b4174	Fis	rpoH	CpxR	rpoH	CpxR	Rob	rpoH	CpxR	
hflX	b4173	rpoH	FNR	Fis	rpoH	CpxR	Fis	rpoH	Fis	
ygjU	b3089	IHF	CRP	Lrp	FNR	IHF	ArcA	IHF		
hfq	b4172	rpoH	FruR	H-NS	CpxR	rpoH	CRP	rpoH		

miaA	b4171	IHF	CRP	ArcA	rpoH	CpxR	CRP	CRP		
yjeS	b4166	CpxR	IHF	DnaA	CpxR	rpoH	Fis	CpxR		
psd	b4160	NikR	CpxR	GntR	CpxR	rpoH	GadE	CpxR		
ygjG	b3073	NtrC	Fis	IHF	NtrC	Fis	IHF	NtrC	Fis	IHF
aer	b3072	FNR	ArcA	CRP	FlhDC	FNR	CpxR	FNR		
yqjI	b3071	FNR	ArcA	CRP	FNR	Fis	FruR	FNR		
frdA	b4154	DcuR	NarL	FNR	NarL	FNR	IHF	NarL	FNR	
frdB	b4153	NarL	DcuR	FNR	NarL	FNR	IHF	NarL	FNR	
frdC	b4152	NarL	ModE	NarP	NarL	FNR	IHF	NarL		
frdD	b4151	Cbl	NarL	CaiF	NarL	FNR	IHF	NarL		
rpoD	b3067	LexA	rpoH	Fis	LexA	rpoH	IHF	LexA	rpoH	
dnaG	b3066	LexA	Fis	IHF	LexA	Fis	CpxR	LexA	Fis	
rpsU	b3065	LexA	Fis	FNR	LexA	Fis	FNR	LexA	Fis	FNR
blc	b4149	Lrp	CRP	FNR	Lrp	FNR	CRP	Lrp	CRP	FNR
efp	b4147	CpxR	GadE	Fis	Fis	FNR	CpxR	CpxR	Fis	
yjeK	b4146	FlhDC	H-NS	PhoB	FlhDC	BaeR	ArgR	FlhDC		
mopA	b4143	rpoH	IHF	CRP	rpoH	CRP	IHF	rpoH	IHF	CRP
mopB	b4142	rpoH	CRP	IHF	rpoH	CRP	IHF	rpoH	CRP	IHF
bacA	b3057	CpxR	MtlR	AsnC	BaeR	NhaR	MtlR	MtlR		
fxsA	b4140	rpoH	TyrR	GadX	rpoH	CRP	IHF	rpoH		
ygiM	b3055	CpxR	H-NS	CRP	Lrp	CpxR	ArcA	CpxR		
b3052	b3052	DeoR	FadR	rpoH	ArgR	rpoH	PurR	rpoH		
aspA	b4139	NarL	CRP	FNR	NarL	CRP	FNR	NarL	CRP	FNR
dcuA	b4138	NarL	Nac	FNR	NarL	FNR	CRP	NarL	FNR	
cadB	b4132	GadX	CadC	GadE	GadE	GadX	H-NS	GadX	GadE	
cadA	b4131	GadE	GadX	CadC	GadE	GadX	H-NS	GadE	GadX	
ygiE	b3040	CpxR	LexA	Fis	FhlA	PurR	CpxR	CpxR		
lysU	b4129	Lrp	IHF	FNR	Lrp	CRP	FNR	Lrp	FNR	
yjdI	b4126	NtrC	IHF	Fis	CRP	FNR	Fis	Fis		
dcuS	b4125	GadX	SoxS	IclR	NarL	MarA	SoxS	SoxS		
dcuB	b4123	DcuR	NarL	FNR	NarL	FNR	FhlA	NarL	FNR	
ygiD	b3039	DgsA	DeoR	Rob	DeoR	CytR	EnvY	DeoR		
fumB	b4122	DcuR	NarL	Fis	NarL	Fur	FNR	NarL		
yjdF	b4121	FhlA	Cbl	AppY	CaiF	FhlA	YiaJ	FhlA		
melA	b4119	Fis	CRP	IHF	CRP	FNR	H-NS	CRP		
adiY	b4116	NarL	FNR	ArcA	ArcA	CRP	H-NS	ArcA		
yjdE	b4115	NarL	FNR	IHF	H-NS	IHF	ArcA	IHF		
proP	b4111	Fis	CRP	H-NS	Fis	CRP	ArcA	Fis	CRP	
yjcZ	b4110	FlhDC	CpxR	H-NS	FlhDC	CpxR	FNR	FlhDC	CpxR	
ygiX	b3025	NarP	QseB	NarL	QseB	CysB	SoxS	QseB		
ygiW	b3024	Fis	CRP	IHF	CRP	IHF	FNR	CRP	IHF	
b3022	b3022	ArcA	FNR	Fis	CRP	FNR	IHF	FNR		
b3021	b3021	IHF	FNR	Fis	CRP	IHF	FNR	IHF	FNR	
phnC	b4106	PhoB	FhlA	GntR	PhoB	CysB	MalT	PhoB		
phnD	b4105	MngR	PhoB	AllR	PhoB	AgaR	MprA	PhoB		
phnF	b4102	AgaR	MngR	AllR	PhoB	AgaR	RcsAB	AgaR		
phnH	b4100	AllR	CusR	PhoB	PhoB	AgaR	RcsAB	PhoB		
ygiR	b3016	Fis	FNR	CRP	Fis	rpoH	CpxR	Fis		
yqhE	b3012	IHF	Fis	CRP	SoxS	IHF	Fis	IHF	Fis	
metC	b3008	MetJ	rpoH	CRP	MetJ	Nac	PurR	MetJ		
exbB	b3006	Fur	FNR	IHF	Fur	CRP	IHF	Fur	IHF	
exbD	b3005	Fur	FNR	IscR	Fur	CRP	IHF	Fur		
b3001	b3001	CRP	FNR	H-NS	CRP	FNR	H-NS	CRP	FNR	H-NS
gyrB	b3699	Fis	LexA	CpxR	Fis	LexA	CpxR	Fis	LexA	CpxR
b3694	b3694	KdpE	FlhDC	ExuR	MtlR	ExuR	AgaR	ExuR		
yidQ	b3688	CpxR	CRP	FNR	CpxR	CRP	H-NS	CpxR	CRP	
ibpA	b3687	rpoH	IHF	CRP	rpoH	IHF	CRP	rpoH	IHF	CRP
ibpB	b3686	rpoH	IHF	Fis	rpoH	IHF	CRP	rpoH	IHF	
pheA	b2599	IHF	FNR	CRP	IHF	CRP	NtrC	IHF	CRP	

yfiA	b2597	IHF	CRP	FNR	CRP	IHF	H-NS	IHF	CRP	
clpB	b2592	rpoH	IHF	CRP	rpoH	CRP	IHF	rpoH	IHF	CRP
ilvB	b3671	CRP	IHF	FNR	CRP	IHF	FNR	CRP	IHF	FNR
kgtP	b2587	ArcA	Fis	CRP	ArcA	CRP	FNR	ArcA	CRP	
ilvN	b3670	CRP	IHF	CpxR	CRP	IHF	FNR	CRP	IHF	
b1499	b1499	H-NS	FlhDC	CpxR	CpxR	FlhDC	rpoH	FlhDC	CpxR	
trxC	b2582	OxyR	PhoB	TrpR	OxyR	Fur	ArcA	OxyR		
b1498	b1498	CRP	Fis	FNR	CRP	FNR	IHF	CRP	FNR	
b1497	b1497	CRP	Fis	IHF	CpxR	Fis	FlhDC	Fis		
ung	b2580	CpxR	H-NS	PhoB	CpxR	LexA	PurR	CpxR		
yddA	b1496	FhlA	Fur	CpxR	RcsAB	CpxR	rpoH	CpxR		
yddB	b1495	CRP	Fis	FNR	Fur	CRP	H-NS	CRP		
gadB	b1493	GadE	GadX	H-NS	GadE	GadX	H-NS	GadE	GadX	H-NS
xasA	b1492	GadE	GadX	H-NS	GadE	GadX	H-NS	GadE	GadX	H-NS
b1491	b1491	RcsAB	NarP	IscR	RcsAB	ArgR	FruR	RcsAB		
b1490	b1490	NtrC	MprA	PhoP	NtrC	FNR	IHF	NtrC		
yfiD	b2579	PdhR	FNR	ArcA	ArcA	PdhR	FNR	PdhR	FNR	ArcA
nlpA	b3661	IHF	Lrp	CRP	IHF	CRP	H-NS	IHF	CRP	
yicL	b3660	PhoB	Fur	NtrC	AgaR	PspF	PhoB	PhoB		
srmB	b2576	Fis	IHF	LexA	Fis	FNR	CRP	Fis		
rpoE	b2573	CpxR	IHF	FNR	CpxR	CRP	FNR	CpxR	FNR	
rseA	b2572	CpxR	H-NS	IHF	CpxR	CRP	FNR	CpxR		
rseB	b2571	CpxR	FruR	NarL	CpxR	FNR	ArcA	CpxR		
ddpA	b1487	NtrC	ArgR	rpoH	NtrC	ArgR	Lrp	NtrC	ArgR	
rseC	b2570	IclR	CpxR	SoxS	CpxR	PhoP	Fis	CpxR		
ddpB	b1486	NtrC	NarL	IdnR	NtrC	ArgR	Lrp	NtrC		
ddpD	b1484	NtrC	NarL	CpxR	NtrC	ArgR	Lrp	NtrC		
phoB	b0399	PhoB	FNR	IHF	PhoB	Fis	SoxS	PhoB		
osmC	b1482	RcsAB	NhaR	Lrp	RcsAB	Lrp	H-NS	RcsAB	Lrp	
b1481	b1481	RcsAB	CRP	ArcA	RcsAB	H-NS	CRP	RcsAB	CRP	
araJ	b0396	AraC	CdaR	CytR	CRP	AraC	FNR	AraC		
rpsV	b1480	RcsAB	OmpR	Fis	RcsAB	OmpR	Fis	RcsAB	OmpR	Fis
aroM	b0390	TyrR	Cbl	NtrC	TrpR	TyrR	CRP	TyrR		
yicH	b3655	CpxR	IHF	Fis	CpxR	PurR	SoxS	CpxR		
lepA	b2569	Fis	Lrp	FNR	Fis	FNR	CRP	Fis	FNR	
recG	b3652	H-NS	GadE	FlhDC	FlhDC	rpoH	FruR	FlhDC		
lepB	b2568	ModE	RcsAB	SoxS	SoxS	CpxR	FhlA	SoxS		
spoU	b3651	FNR	H-NS	ArcA	rpoH	CpxR	H-NS	H-NS		
rnc	b2567	H-NS	Fis	FlhDC	Fis	CpxR	CRP	Fis		
spoT	b3650	rpoH	H-NS	FruR	rpoH	CRP	Fis	rpoH		
era	b2566	FruR	FlhDC	CpxR	FlhDC	Fis	CpxR	FlhDC	CpxR	
recO	b2565	CpxR	FlhDC	Fis	PhoB	CsgD	FlhDC	FlhDC		
sfcA	b1479	NarL	FruR	GalR	FruR	Fis	PhoP	FruR		
fdnI	b1476	NarP	NarL	AgaR	NarP	NarL	FNR	NarP	NarL	
fdnH	b1475	NarP	NarL	CsgD	NarP	NarL	FNR	NarP	NarL	
fdnG	b1474	NarP	NarL	CaiF	NarP	NarL	FNR	NarP	NarL	
yaiA	b0389	TyrR	TrpR	ArgP	TrpR	TyrR	CRP	TyrR	TrpR	
aroL	b0388	TyrR	TrpR	CRP	TrpR	TyrR	CRP	TyrR	TrpR	CRP
yaiC	b0385	CsgD	Rob	DicA	CsgD	FadR	GntR	CsgD		
psiF	b0384	PhoB	EvgA	GadW	PhoB	CRP	FNR	PhoB		
phoA	b0383	PhoB	Fur	FNR	PhoB	CRP	FNR	PhoB	FNR	
yaiB	b0382	CRP	Fis	IHF	CRP	ArcA	IHF	CRP	IHF	
gmk	b3648	rpoH	CpxR	LexA	CpxR	LexA	Fis	CpxR	LexA	
dinD	b3645	CRP	IHF	FNR	LexA	CRP	ArcA	CRP		
rph	b3643	FNR	Fis	CRP	FNR	Fis	IHF	FNR	Fis	
pyrE	b3642	MprA	DnaA	RcsAB	BaeR	RcsAB	PhoB	RcsAB		
yfhD	b2558	Rob	Fis	Fur	Rob	BaeR	RcsAB	Rob		
ttk	b3641	PhoP	FruR	Lrp	Lrp	FNR	IHF	Lrp		
purL	b2557	PurR	PhoP	CRP	PurR	PhoP	Lrp	PurR	PhoP	

yfhA	b2554	FhlA	YiaJ	XylR	CaiF	HyfR	YiaJ	YiaJ		
narU	b1469	GadW	RcsAB	CsgD	RcsAB	UxuR	ArgR	RcsAB		
glyA	b2551	MetR	PurR	FNR	MetR	PurR	CRP	MetR	PurR	
b1463	b1463	NtrC	NarL	CRP	PhoP	NtrC	LexA	NtrC		
yaiT	b0371	NarL	Fur	DicA	rpoH	NarL	Fur	NarL	Fur	
dfp	b3639	FNR	CRP	Fis	Fis	CRP	SoxS	CRP	Fis	
radC	b3638	AgaR	ExuR	LacI	FadR	AgaR	FlhDC	AgaR		
rpmB	b3637	Fis	FNR	CRP	FNR	CpxR	Fis	Fis	FNR	
rpmG	b3636	FNR	FadR	DeoR	FNR	CRP	Fis	FNR		
mutM	b3635	rpoH	Nac	SoxS	rpoH	FlhDC	FruR	rpoH		
rfaQ	b3632	rpoH	PhoP	Fis	rpoH	CpxR	IHF	rpoH		
yphF	b2548	Cbl	PdhR	GntR	GntR	EvgA	NagC	GntR		
rfaG	b3631	LexA	SoxS	rpoH	CpxR	FlhDC	rpoH	rpoH		
rfaP	b3630	FNR	Fis	CRP	CpxR	IHF	CRP	CRP		
yphC	b2545	Rob	DeoR	CsgD	RcsAB	UxuR	Rob	Rob		
hcaD	b2542	HcaR	GntR	RcsAB	RcsAB	NtrC	NagC	RcsAB		
b1458	b1458	CpxR	CRP	FNR	LexA	CRP	IHF	CRP		
hemB	b0369	rpoH	Fis	NarL	SoxS	rpoH	CpxR	rpoH		
b1452	b1452	Fur	CRP	FNR	Fur	CRP	IHF	Fur	CRP	
tauB	b0366	Cbl	CysB	FadR	CysB	FNR	CpxR	CysB		
b1450	b1450	Fis	CRP	rpoH	PhoB	CRP	Fis	Fis	CRP	
tauA	b0365	Cbl	CysB	AllR	CysB	FNR	ModE	CysB		
rfaS	b3629	rpoH	SoxS	Fis	CpxR	CRP	Fis	Fis		
rfaJ	b3626	CRP	Fis	H-NS	CRP	rpoH	CpxR	CRP		
rfaZ	b3624	MalT	rpoH	LrhA	rpoH	CpxR	Fis	rpoH		
rfaK	b3623	CpxR	H-NS	PhoP	CRP	rpoH	CpxR	CpxR		
rfaL	b3622	rpoH	H-NS	CpxR	rpoH	CRP	IHF	rpoH		
hcaA1	b2538	HcaR	ExuR	PhoB	PhoB	CysB	PhoP	PhoB		
rfaC	b3621	rpoH	H-NS	CpxR	rpoH	Fis	FNR	rpoH		
hcaR	b2537	HcaR	CRP	Fis	DnaA	Fis	NtrC	Fis		
rfaF	b3620	rpoH	Lrp	CpxR	rpoH	CpxR	Fis	rpoH	CpxR	
csiE	b2535	H-NS	CRP	Lrp	H-NS	CRP	FNR	H-NS	CRP	
b2531	b2531	IscR	CRP	IHF	IscR	CRP	IHF	IscR	CRP	IHF
b1447	b1447	H-NS	CpxR	OxyR	FlhDC	CpxR	SoxS	CpxR		
yfhO	b2530	IscR	CRP	FNR	IscR	CRP	FNR	IscR	CRP	FNR
b1446	b1446	ModE	Rob	SoxS	PhoB	rpoH	Rob	Rob		
yaiN	b0357	FadR	FruR	CRP	CRP	IHF	FNR	CRP		
adhC	b0356	FadR	FruR	CRP	CRP	IHF	rpoH	CRP		
mhpT	b0353	OxyR	rpoH	GntR	GntR	NagC	PurR	GntR		
rfaD	b3619	rpoH	NarL	Fis	rpoH	GadE	Lrp	rpoH		
htrL	b3618	CRP	Fis	H-NS	CRP	CpxR	ArcA	CRP		
kbl	b3617	Lrp	CRP	Fis	Lrp	CRP	IHF	Lrp	CRP	
tdh	b3616	Lrp	Fis	H-NS	Lrp	CRP	IHF	Lrp		
yibP	b3613	LexA	Fis	H-NS	ArgR	FlhDC	LexA	LexA		
b2529	b2529	IscR	FNR	Fur	IscR	CRP	FNR	IscR	FNR	
yfhF	b2528	IscR	FNR	IHF	IscR	CRP	IHF	IscR	IHF	
yfhE	b2527	IscR	CRP	H-NS	CRP	IscR	IHF	IscR	CRP	
hscA	b2526	IscR	Fis	FNR	FNR	IHF	ArcA	FNR		
fdx	b2525	FNR	Fur	IHF	FNR	IHF	Fis	FNR	IHF	
yfhJ	b2524	IscR	H-NS	FNR	FNR	IHF	ArcA	FNR		
b1437	b1437	LsrR	FlhDC	CpxR	RcsAB	FlhDC	NtrC	FlhDC		
mhpC	b0349	YiaJ	MhpR	AllR	ArgR	NagC	MhpR	MhpR		
b1431	b1431	EnvY	MprA	NtrC	Fis	NtrC	CRP	NtrC		
mhpR	b0346	CRP	ArcA	FNR	NtrC	Fis	CRP	CRP		
lacI	b0345	NtrC	FucR	AraC	CRP	NtrC	IHF	NtrC		
lacZ	b0344	H-NS	CRP	Fis	H-NS	CRP	LacI	H-NS	CRP	
lacY	b0343	H-NS	Fis	CRP	H-NS	CRP	FNR	H-NS	CRP	
lacA	b0342	H-NS	CRP	Fis	H-NS	CRP	FNR	H-NS	CRP	
gpsA	b3608	FruR	Fis	rpoH	rpoH	NagC	IHF	rpoH		

cysE	b3607	CpxR	CusR	GntR	CpxR	FNR	ArgR	CpxR		
lldD	b3605	PdhR	ArcA	FlhDC	ArcA	CRP	Fis	ArcA		
lldR	b3604	PdhR	ArcA	IHF	ArcA	PdhR	CRP	PdhR	ArcA	
lldP	b3603	PdhR	ArcA	CRP	ArcA	PdhR	CRP	PdhR	ArcA	CRP
ndk	b2518	Fis	CRP	IHF	CRP	Fis	FNR	Fis	CRP	
mtlR	b3601	FruR	Fis	H-NS	FruR	Fis	PhoP	FruR	Fis	
yfgB	b2517	rpoH	FNR	Fis	Fis	rpoH	ArcA	rpoH	Fis	
mtlD	b3600	FruR	Fis	GadE	FruR	Fis	CRP	FruR	Fis	
yfgA	b2516	ArgR	rpoH	FlhDC	rpoH	CpxR	NagC	rpoH		
gcpE	b2515	FNR	rpoH	ArgR	rpoH	CpxR	SoxS	rpoH		
b1428	b1428	NtrC	CRP	FNR	SoxS	MarA	NtrC	NtrC		
b2511	b2511	ArgR	FruR	Fis	Fis	PurR	NarL	Fis		
b1422	b1422	CRP	FNR	ArcA	CRP	IHF	Fis	CRP		
codA	b0337	Nac	PurR	CRP	Nac	PurR	IHF	Nac	PurR	
trg	b1421	H-NS	CRP	FNR	FlhDC	CpxR	FNR	FNR		
codB	b0336	Nac	PurR	CRP	Nac	PurR	IHF	Nac	PurR	
prpE	b0335	AllR	FucR	PrpR	AllR	FadR	NagC	AllR		
prpD	b0334	PrpR	Fis	GntR	Fis	IHF	CRP	Fis		
prpC	b0333	PrpR	CRP	Fis	NagC	Fis	CRP	CRP	Fis	
guaB	b2508	PurR	DnaA	Fis	PurR	Fis	DnaA	PurR	DnaA	Fis
guaA	b2507	PurR	DnaA	Fis	PurR	Fis	CRP	PurR	Fis	
aldA	b1415	DnaA	ArcA	CRP	CRP	ArcA	DnaA	DnaA	ArcA	CRP
ydcF	b1414	NtrC	FlhDC	CpxR	RcsAB	TrpR	CpxR	CpxR		
b1410	b1410	GntR	AllR	RcsAB	UxuR	RcsAB	NagC	RcsAB		
yahG	b0321	SoxS	MarA	PhoB	AgaR	PhoB	NtrC	PhoB		
betI	b0313	ArcA	BetI	FNR	ArcA	CRP	PhoP	ArcA		
betB	b0312	ArcA	FNR	IHF	ArcA	CRP	Fis	ArcA		
torD	b0998	TorR	NarL	NarP	NarL	NagC	NtrC	NarL		
torS	b0993	CdaR	Cbl	Rob	NarL	Rob	NtrC	Rob		
appA	b0980	AppY	NarL	Rob	ArcA	NarL	H-NS	NarL		
phnJ	b4098	AllR	PhoB	CaiF	PhoB	AgaR	CysB	PhoB		
phnK	b4097	AllR	CaiF	PhoB	AgaR	NsrR	PhoB	PhoB		
phnL	b4096	PhoB	NarP	NarL	AgaR	PhoB	MprA	PhoB		
appB	b0979	AppY	NarP	ArcA	ArcA	NarL	H-NS	ArcA		
appC	b0978	AppY	ArcA	H-NS	ArcA	NarL	H-NS	ArcA	H-NS	
hyaF	b0977	NarP	IscR	AppY	NarP	NarL	ArcA	NarP		
hyaE	b0976	NarP	IscR	NarL	NarP	NarL	IscR	NarP	IscR	NarL
hyaD	b0975	NarP	IscR	NarL	NarP	NarL	IscR	NarP	IscR	NarL
hyaC	b0974	NarP	IscR	NarL	NarP	NarL	IscR	NarP	IscR	NarL
hyaB	b0973	NarP	IscR	AppY	NarP	NarL	ArcA	NarP		
hyaA	b0972	NarP	IscR	NarL	NarP	NarL	IscR	NarP	IscR	NarL
yccA	b0970	CpxR	FNR	CRP	CpxR	CRP	rpoH	CpxR	CRP	
yjcV	b4086	GntR	AscG	HU	GntR	AgaR	NagC	GntR		
yccV	b0966	rpoH	FruR	H-NS	CRP	rpoH	H-NS	rpoH	H-NS	
b0965	b0965	NtrC	AllS	OxyR	NtrC	PhoB	CpxR	NtrC		
yjcQ	b4081	NanR	AgaR	TdcA	AgaR	CsgD	NsrR	AgaR		
sulA	b0958	LexA	ArcA	CRP	LexA	CRP	ArcA	LexA	ArcA	CRP
nrfE	b4074	AllR	AtoC	NarP	NarP	AgaR	Rob	NarP		
ompA	b0957	CRP	FruR	ArcA	CRP	IHF	CpxR	CRP		
nrfD	b4073	NarP	MngR	AtoC	FlhDC	NarP	NarL	NarP		
nrfC	b4072	MngR	NarP	ZraR	NarP	NarL	FlhDC	NarP		
nrfA	b4070	NarP	PdhR	RcsAB	NarP	NarL	FlhDC	NarP		
pqiB	b0951	MarA	SoxS	rpoH	SoxS	MarA	Fis	MarA	SoxS	
pqiA	b0950	MarA	SoxS	TrpR	SoxS	MarA	RcsAB	MarA	SoxS	
acs	b4069	Fis	CRP	IHF	Fis	IHF	CRP	Fis	CRP	IHF
yjcH	b4068	Fis	CRP	H-NS	Fis	IHF	CRP	Fis	CRP	
yjcG	b4067	Fis	H-NS	CRP	Fis	IHF	CRP	Fis	CRP	
uup	b0949	Fis	H-NS	FlhDC	Fis	SoxS	FNR	Fis		
soxS	b4062	SoxS	SoxR	LexA	SoxS	CRP	IHF	SoxS		

pyrD	b0945	PurR	FNR	IHF	PurR	FNR	IHF	PurR	FNR	IHF
ycbF	b0944	CusR	NagC	GntR	UxuR	RcsAB	NagC	NagC		
yjcB	b4060	CRP	Fur	FNR	CRP	ArcA	IHF	CRP		
ssb	b4059	LexA	NhaR	PurR	LexA	rpoH	CpxR	LexA		
uvrA	b4058	LexA	ArcA	Lrp	LexA	ArcA	CRP	LexA	ArcA	
yjbQ	b4056	Fur	FNR	NarL	CRP	FNR	H-NS	FNR		
tyrB	b4054	TyrR	FruR	Lrp	TyrR	CpxR	IHF	TyrR		
ssuC	b0934	Cbl	NagC	CusR	NagC	PurR	PhoB	NagC		
pncB	b0931	CRP	Fis	IHF	Lrp	CpxR	CRP	CRP		
asnS	b0930	FNR	CRP	Fis	NagC	Fis	PhoP	Fis		
