# Supplementary material for: Reconstruction of Escherichia coli transcriptional regulatory networks via regulon-based associations
Source: BMC Syst Biol. 2009 Apr 14;3:39. doi: 10.1186/1752-0509-3-39 (PMC2689187; doi:10.1186/1752-0509-3-39)
Supplement: Additional file 4 — TF-Operon Refined Interactions. A refined catalog of transcriptional interactions. [file 1752-0509-3-39-S4.pdf]

Operon Name	Common Regulator 1	Common Regulator 2	Common Regulator 3
aaeXAB	HyfR	AgaR	CpxR
aas-lplT	AcrR	AllS	AscG
aat	AllS	AraC	Cbl
abgABT-ogt	NarL	CpxR	NhaR
abgR	CRP	FNR	FlhDC
abrB	FlhDC	CpxR	Fur
accA	CpxR	Fis	FlhDC
accBC	FNR	CRP	Fis
accD	LexA	Fis	Fur
aceBAK	FruR	ArcA	IclR
ackA-pta	ArcA	FNR	CRP
acnA	FruR	ArcA	Fis
acnB	ArcA	Fis	FruR
acpH	AllR	CpxR	FlhDC
acrAB	Rob	SoxS	MarA
acrD	BaeR	EvgA	GntR
acrEF	YiaJ	AgaR	AllR
acrR	AcrR	FruR	LsrR
acs-yjcH-actP	CRP	Fis	IHF
ada-alkB	RcsAB	UxuR	Ada
add	CRP	FNR	Fis
ade	AllS	CadC	GadW
adhE	Fis	FruR	NarL
adhP	CRP	Fis	FruR
adiA	FNR	H-NS	IHF
adiC	IHF	ArcA	FNR
adiY	ArcA	CRP	FNR
adk	CRP	FNR	Fis
adrA	CsgD	DicA	FadR
aegA	AgaR	AllR	FlhDC
aer	FNR	ArcA	CRP
aes	AllR	FadR	FlhDC
agaR	AgaR	CpxR	ExuR
agaS-kbaY-agaBCDI	AgaR	NagC	Cbl
agp	CRP	FNR	Fis
ahpCF	MetJ	OxyR	CRP
aidB	Ada	Lrp	H-NS
alaS	FNR	Fis	FruR
aldA	ArcA	CRP	DnaA
aldB	CRP	Fis	IHF
alkA	AllR	NagC	NarP
allA	AgaR	AllR	HyfR
allDC-ylbA	AllS	FlhDC	GntR
allR	Lrp	AraC	ArgR
allS	AllR	AcrR	GntR
alpA	AllR	GntR	GutM
alr	ArgR	AscG	EvgA
amiA-hemF	AgaR	AllR	AllS
amn	CRP	FNR	Fis
ampC	Fur	H-NS	ModE
ampDE	BetI	CRP	Cbl
ampG	AllR	FhlA	GadX
ampH	CpxR	CsgD	PhoB
amyA	NtrC	IHF	Lrp
anmK	LeuO	MarR	NagC
ansA-pncA	PhoP	CRP	AppY
ansB	AraC	CRP	CueR
ansP	FNR	MarA	NtrC

apbE	LeuO	MetJ	OxyR
aphA	CRP	FNR	FlhDC
appCB-yccB-appA	ArcA	H-NS	NarL
appY	H-NS	CRP	FNR
apt	Fis	ArcA	CRP
aqpZ	AllR	ArgR	AscG
araBAD	AraC	CRP	H-NS
araC	AraC	CRP	DcuR
araE	AraC	CRP	Cbl
araFGH	AraC	CRP	H-NS
araJ	AraC	CRP	CdaR
arcA	CRP	FNR	ArcA
argA	ArgR	CRP	IHF
argCBH	ArgR	CRP	IHF
argD	ArgR	PhoP	CRP
argE	ArgR	CRP	GadE
argF	ArgR	IHF	CRP
argG	ArgR	CRP	IHF
argI	ArgR	CRP	IHF
argO	CpxR	FNR	Fis
argP	PhoB	ArgR	FhlA
argR	ArgR	CytR	MelR
argS	ArcA	CRP	FNR
argT-hisJQMP	NtrC	IHF	ArgR
arnBCADTEF	FlhDC	CpxR	IHF
aroF-tyrA	TyrR	CRP	IHF
aroG	CpxR	TyrR	CRP
aroH	TrpR	CRP	FNR
aroKB-damX-dam-rpe-gph-trpS	CpxR	Fis	rpoH
aroL-yaiA-aroM	TyrR	TrpR	CRP
aroP	CRP	TyrR	IHF
arpA	FNR	FlhDC	GntR
arsRBC	RcsAB	AgaR	AllR
artJ	ArgR	CRP	IHF
artPIQM	IHF	ArcA	CRP
ascFB	AscG	CytR	HyfR
ascG	AraC	AscG	HyfR
asd	CRP	Lrp	FNR
aslA	FlhDC	MngR	NarP
aslB	Rob	Cbl	GntR
asmA	AsnC	LexA	Nac
asnA	AgaR	AllR	AsnC
asnB	GadX	ArgR	CRP
asnC-mioC-mnmG-gidB	Nac	AsnC	Fis
asnS	Fis	CRP	FNR
aspA-dcuA	FNR	NarL	CRP
aspC	ArgR	CRP	FNR
aspS	FNR	Fis	CRP
asr	CRP	PhoB	ArcA
astCADBE	ArgR	NtrC	Lrp
atl	ArgR	CpxR	CsgD
atoDAEB	AtoC	NagC	AgaR
atoSC	GalR	GalS	GntR
atpIBEFHAGDC	FNR	Fis	CRP
avtA	IHF	FNR	FlhDC
azoR	CsgD	LexA	Nac
bacA	MtlR	AsnC	BaeR
barA	NarP	CaiF	GadX
basRS	FlhDC	Fur	LexA

bax	FNR	Fis	CRP
bcr	MetJ	MetR	NarP
bcsABZC	CsgD	AgaR	PhoB
bcsEFG	CpxR	DnaA	ExuR
bdm-sra	RcsAB	CRP	Fis
betIBA	ArcA	CRP	FNR
bfd-bfr	CRP	FNR	Fur
bglA	CpxR	MetR	PhoB
bglGFB	Fis	H-NS	FlhDC
bglH-yieLK	GntR	BaeR	Cbl
bglX	AscG	CdaR	LrhA
bhsA	CRP	FNR	IHF
bioA	ArgR	CRP	CpxR
bioBFCD	CRP	CpxR	H-NS
bioH	AraC	IdnR	LexA
bisC	UlaR	BaeR	DicA
blc	CRP	FNR	Lrp
bolA	H-NS	OmpR	CRP
borD	CRP	IHF	PhoP
brnQ-proY	AppY	FhlA	Fur
bssR	ArcA	FNR	CRP
bssS	rpoH	CRP	CaiF
btuB-murI	Fis	CpxR	PhoP
btuCED	Lrp	MhpR	Cbl
cadBA	GadE	GadX	CadC
cadC	CytR	FNR	GalR
caiF	H-NS	NarL	FNR
caiTABCDE	CaiF	AllR	NtrC
can	CRP	CpxR	FNR
carAB	ArgR	IHF	PurR
cbl	CysB	NtrC	ArcA
cbpAM	H-NS	Lrp	AppY
cbrB	Cbl	FhlA	GntR
cbrC	AraC	CpxR	DicA
cdaR	CdaR	CRP	FNR
cdd	CRP	CytR	FNR
cdh	ArgR	CpxR	FruR
cdsA	AllS	CRP	Fis
cedA	NagC	NikR	NtrC
cfa	CRP	FNR	H-NS
chaA	CpxR	CRP	Fis
chaBC	PhoB	CRP	CpxR
chbBCARFG	NagC	ChbR	CusR
cho	CpxR	FlhDC	LexA
chpSB	DhaR	CRP	Cbl
cirA	CRP	Fur	FNR
citCDEFXG	AgaR	NagC	PhoB
citT	Cbl	CdaR	CsgD
clcA	CdaR	CpxR	DicA
clcB	RcsAB	FhlA	FlhDC
cld	CRP	FNR	Fis
clpA	ArcA	CRP	FNR
clpB	CRP	IHF	rpoH
clpPX-lon	rpoH	CRP	IHF
clpS	IHF	CRP	FruR
cls-yciU	PhoP	BaeR	DnaA
cmk-rpsA-ihfB	FNR	Fis	CRP
cmr	FadR	Fur	Nac
cmtBA-yggPFDC	AgaR	AtoC	CaiF

cnu	CRP	FNR	IHF
coaA	CRP	CpxR	LrhA
coaE-yacFG	FlhDC	NagC	ArgR
cobUST	ModE	PhoP	FruR
codBA	Nac	PurR	CRP
cof	AtoC	Cbl	CpxR
copA	CRP	CueR	FlhDC
corA	FNR	PhoP	CRP
cpdB	AraC	CRP	FruR
cpxRA	CpxR	AgaR	ExuR
crcB	OxyR	CpxR	GntR
creABCD	GntR	PhoB	AraC
crl	ArcA	CRP	FNR
crp	CRP	FruR	IHF
csdAE	FlhDC	FruR	RcsAB
csgBAC	CpxR	CsgD	ArgR
csgDEFG	CsgD	RcsAB	CpxR
csiD-ygaF-gabDTP	H-NS	Lrp	Nac
csiE	CRP	H-NS	FNR
csiR	ArgR	Fis	H-NS
cspA	CRP	FNR	Fis
cspB	AraC	CRP	FNR
cspD	H-NS	ArcA	CRP
cspE	CRP	FNR	GntR
cspF	CRP	CpxR	CytR
cspH	ArcA	ArgP	CRP
cspI	ArcA	CRP	FNR
csrA	CRP	CpxR	IHF
csrD	Cbl	CpxR	FruR
cstA	ArcA	CRP	FNR
cueR	GadX	IHF	Lrp
cusCFBA	CusR	CRP	FNR
cusRS	CusR	BirA	CsgD
cutC	CRP	Fur	IHF
cvpA-purF-ubiX	PurR	CRP	FNR
cvrA	FlhDC	MalT	NhaR
cyaA	CRP	CpxR	FNR
cyaY	ArgR	CpxR	GadE
cybB	CRP	FNR	Fur
cycA	ArcA	Fis	FruR
cydAB	ArcA	FruR	H-NS
cydDC	NarL	PhoB	NtrC
cynTSX	NarL	FhlA	Fur
cyoABCDE	ArcA	Fur	GadE
cysB	CysB	CpxR	FNR
cysDNC	CysB	IHF	CRP
cysE	CpxR	ArgR	CusR
cysJIH	CysB	IHF	CRP
cysK	CRP	CysB	IHF
cysPUWAM	CysB	IHF	CRP
cysQ	CpxR	HU	NtrC
cysS	CpxR	FadR	FruR
cysZ	ArgR	FadR	MetR
cytR	CytR	CRP	FNR
dacA	Fis	CRP	CpxR
dacC	FNR	ArcA	CRP
dacD	Cbl	CpxR	CsgD
dadAX	CRP	Lrp	FNR
dapA-nlpB	ArgR	CpxR	Lrp

dapB	CRP	CpxR	Fur
dcm-vsr	FlhDC	GadE	H-NS
dcp	ArcA	CpxR	Fis
dcrB	CRP	FNR	Fis
dctA	ArcA	CRP	DcuR
dcuB-fumB	NarL	FNR	DcuR
dcuC	AgaR	ArcA	FNR
dcuD	PhoP	BaeR	CRP
dcuSR	NarL	SoxS	GadX
dcyD-yecSC	FlhDC	Lrp	FNR
ddlA	AraC	CpxR	IHF
ddpXABCDF	NtrC	ArgR	Lrp
deaD	FNR	Fis	CRP
def-fmt	ArgR	CpxR	H-NS
degP	CpxR	H-NS	CRP
degQS	Ada	CsgD	FNR
deoCABD	CytR	ModE	DeoR
deoR	PhoP	Nac	NarL
dfp	CRP	Fis	FNR
dgkA	CpxR	Fis	GalR
dgoRKADT	AgaR	AppY	AtoC
dgsA-ynfK	DgsA	IHF	NarL
dgt	PhoB	CysB	HcaR
dhaKLM	DhaR	CRP	FNR
dhaR	LexA	ModE	NarL
diaA	CpxR	ArgR	H-NS
dicA	CRP	FNR	IHF
dicB-ydfDE-insD-intQ	CpxR	DicA	FlhDC
dicC-ydfXW	ArgR	FhlA	FlhDC
dinB	LexA	ArcA	CRP
dinD	CRP	ArcA	FNR
dinG	LexA	ArcA	CRP
dinI	LexA	ArcA	CRP
dinJ-yafQ	CRP	CpxR	FNR
dipZ-yjdC	SoxS	CRP	ChbR
dkgA	Fis	IHF	CRP
dkgB	CsgD	CysB	GadX
dksA	Fis	CRP	FNR
dld	FNR	ArcA	ArgR
dmsABC	ModE	NarL	FNR
dnaAN-recF	DnaA	Fis	LexA
dnaB	CpxR	GadE	LexA
dnaK-tpke11-dnaJ	CRP	rpoH	IHF
dnaQ	CpxR	CysB	FlhDC
dnaX	Fis	CpxR	FNR
dpiBA	AgaR	AsnC	CsgD
dppABCDF	FNR	IHF	CRP
dps	IHF	OxyR	CRP
dsbB	CpxR	Fis	Fur
dsbG	ArgR	AscG	GutM
dsdC	FlhDC	CpxR	LexA
dsdXA	FlhDC	CRP	CpxR
dsrB	AlsR	CpxR	DhaR
dusA	rpoH	CpxR	GadX
dusB-fis	CRP	Fis	IHF
dusC	AsnC	FadR	OmpR
dut-slmA	FNR	Lrp	CRP
dxr	CpxR	Lrp	Nac
eaeH-insE-1EF-1F-1	CsgD	EvgA	HU

eamA	CRP	Fis	GadE
eamB	FNR	FlhDC	Fur
ebgR	BaeR	GalR	GalS
eco	CRP	FNR	Lrp
ecpD-htrE	GntR	HU	IHF
edd-eda	GntR	FruR	PhoB
efeOB	CRP	FNR	Fur
efeU_1U_2	CRP	Fur	GntR
efp	CpxR	Fis	FNR
elaA	CaiF	CpxR	DhaR
elaB	Fis	IHF	Lrp
elaD	AgaR	AllS	EvgA
elbB-mtgA	BirA	CpxR	CsgD
emrD	CpxR	FruR	MetJ
emrKY	EvgA	AllR	CpxR
emtA	CpxR	Fis	Fur
endA	CaiF	Fis	FruR
entCEBAH	CRP	Fur	IHF
entS	Fur	CRP	FNR
envR	Ada	CpxR	Fis
envY-ompT	CRP	IHF	FNR
epd-pgk-fbaA	FruR	CRP	Fis
eptA	Cbl	FlhDC	IHF
eptB	CpxR	FruR	H-NS
erfK	FNR	Fis	FruR
essD-ybcS-rzpD	AllR	Fur	AppY
essQ-ydfRQP	CpxR	FlhDC	Fur
eutBCLKR	CpxR	NarL	NarP
eutHA	AgaR	AllS	AsnC
eutNEJG	AgaR	GntR	NagC
eutSPQTDM	NagC	AgaR	AcrR
evgAS	EvgA	AgaR	BaeR
exbBD	Fur	IHF	CRP
exuR	ExuR	CpxR	H-NS
exuT	ExuR	FadR	GadX
fabA	FadR	Fis	H-NS
fabB	FadR	CRP	CpxR
fabI	FruR	CpxR	FNR
fabR-yijD	CpxR	Lrp	NarL
fadBA	FadR	ArcA	Fis
fadD	ArcA	CRP	FadR
fadE	FadR	ArcA	ArgR
fadH	ArcA	CRP	FlhDC
fadIJ	FadR	ArcA	CRP
fadL	FadR	OmpR	PhoP
fadR	FlhDC	BaeR	NagC
fbaB	CRP	FNR	Fis
fbp	CRP	FNR	Fis
fdnGHI	NarL	NarP	FNR
fdoGHI-fdhE	IHF	CRP	FNR
fdrA-ylbE_1E_2F-ybcF	AllR	NagC	PhoB
feaB	FlhDC	GntR	ModE
feaR	CRP	Fis	H-NS
fecABCDE	Fur	CRP	FNR
fecIR	CRP	Fur	IHF
feoAB	FNR	Fur	CRP
fepA-entD	CRP	Fur	IHF
fepB	Fur	CRP	CpxR
fepDGC	Fur	OxyR	CpxR

fes-ybdZ-entF-fepE	Fur	CRP	FNR
ffh	Fis	CpxR	FNR
fhuACDB	Fur	CRP	CpxR
fhuE	CRP	Fur	IHF
fhuF	Fur	IHF	CRP
fimAICDFGH	H-NS	Lrp	IHF
fimB	H-NS	CRP	IHF
fimE	AraC	CusR	H-NS
fimZ	CRP	CpxR	CusR
fiu	CRP	Fur	ArgR
fixABCX	CaiF	AllR	NagC
fklB	FNR	Fis	Fur
fkpA	CpxR	FNR	Fis
fldA-uof-fur	SoxS	FNR	OxyR
fldB	SoxS	CpxR	Nac
flgAMN	FlhDC	H-NS	CpxR
flgBCDEFGHIJ	FlhDC	FNR	H-NS
flgKL	FlhDC	H-NS	CpxR
flhBAE	FlhDC	CpxR	H-NS
flhDC	RcsAB	Fur	LrhA
fliAZY	FlhDC	H-NS	CpxR
fliC	GadE	H-NS	CpxR
fliDST	FlhDC	CpxR	FNR
fliE	FlhDC	AgaR	FNR
fliFGHIJK	FlhDC	FNR	H-NS
fliLMNOPQR	FlhDC	H-NS	FNR
flk	FadR	LexA	Nac
flu	OxyR	ArcA	FNR
flxA	FlhDC	H-NS	CpxR
fnr	FNR	ArcA	CRP
focA-pflB	ArcA	NarL	FNR
folA	BaeR	CpxR	Fis
folC-dedD	AgaR	ArgR	CRP
folD-ybcJ	CpxR	ArgR	CRP
folE-yeiB	CRP	FNR	Fis
folP-glmM	Fis	rpoH	EvgA
folX-yfcH	PhoB	ArgR	CRP
fpr	MarA	SoxS	CRP
frc	CpxR	FNR	H-NS
frdABCD	NarL	FNR	IHF
fre	NarL	Fis	Lrp
frlABCDR	BaeR	IdnR	RcsAB
frmRAB	CRP	FadR	IHF
frr	FNR	CpxR	Fur
frsA	CysB	H-NS	ModE
fruBKA	FruR	CRP	FNR
frvABXR	AgaR	NagC	AtoC
frwCB	AraC	CaiF	FhlA
fryBC-ypdFE-fryA	AgaR	NagC	AllR
fsaA	AgaR	Cbl	FlhDC
fsr	ArgR	CpxR	FlhDC
ftnA	FNR	IHF	CRP
ftnB	CpxR	ArgR	CRP
ftsB	FlhDC	ArgR	Fis
ftsK	LexA	H-NS	Nac
ftsP	BaeR	CpxR	FadR
ftsYEX	rpoH	CpxR	FNR
fucAO	FucR	Fis	GntR
fucPIKUR	FucR	CRP	CpxR

fumA	ArcA	CRP	FNR
fxsA	rpoH	CRP	GadX
gadAXW	GadE	GadX	TorR
gadBC	GadE	GadX	H-NS
gadE-mdtEF	FNR	ArcA	EvgA
galETKM	GalR	GalS	HU
galF	LexA	CRP	Fis
galP	CRP	GalR	GalS
galR	AraC	DnaA	FucR
galS	GalR	GalS	CRP
galU	CpxR	Fis	FruR
gapA-yeaD	CRP	FruR	IHF
gapC_1C_2	CRP	H-NS	IHF
garD	CRP	CdaR	FNR
garPLRK-rnpB	CdaR	FNR	CRP
gatR_2	ArcA	CRP	CpxR
gatYZABCD	ArcA	CRP	FNR
gcl-hyi-glxR-ybbVW-allB-ybbY-glxK	AllR	FruR	NagC
gcvA	Fis	FruR	IscR
gcvR-bcp	CRP	CpxR	Lrp
gcvTHP	Lrp	PurR	GcvA
gdhA	CRP	Nac	IHF
gfcA	AraC	Cbl	CdaR
gfcBCD	Rob	CpxR	NagC
gfcE-etp-etk	ArgR	NagC	AcrR
ggt	GadE	GadX	HcaR
ghrA	ArgR	FlhDC	H-NS
ghrB	FNR	Fur	H-NS
glcC	CRP	GlcC	ArcA
glcDEFGBA	ArcA	IHF	GlcC
glf-rfc	AppY	ArcA	CRP
glgBX	FNR	CRP	IHF
glgCAP	CRP	FNR	IHF
glgS	CRP	EnvY	FNR
glk	FruR	CRP	FNR
glmUS	NagC	CRP	FNR
glnALG	NtrC	CRP	Fis
glnB	ArgR	Fis	GalR
glnHPQ	IHF	NtrC	Fis
glnK-amtB	GadX	NtrC	IHF
glnS	ArcA	FNR	Fis
gloA	ArcA	CpxR	CsgD
gloB	PhoB	CpxR	DnaA
glpABC	ArcA	FlhDC	FNR
glpEGR	LexA	Fis	Nac
glpFKX	FNR	CRP	IHF
glpTQ	CRP	FNR	GlpR
gltA	ArcA	CRP	IHF
gltBDF	ArgR	Nac	GadE
gltI-sroC-gltJKL	FlhDC	NtrC	AraC
gltP	Cbl	LacI	LexA
gltS	CpxR	GntR	IHF
gltX	Fis	CRP	FNR
glvCBG-ysdC	GntR	IdnR	NtrC
glyA	MetR	PurR	CRP
glyQS	CpxR	FNR	rpoH
gmhB	AgaR	NagC	NarL
gmk	CpxR	LexA	Fis
gmr	Fur	NtrC	CpxR

gnd	GadE	Fis	IHF
gnsB	CRP	FNR	IHF
gntP	UxuR	AgaR	CRP
gntRKU	GntR	IdnR	CRP
gntT	CRP	GntR	FlhDC
gntXY	CRP	GntR	IscR
gor	OxyR	FhlA	GntR
gpmA	Fur	CRP	FNR
gpmM-envC-yibQ	LexA	ArgR	Fis
gpp	ArgR	CpxR	IscR
gpt	Fis	CRP	FNR
greA	FNR	Fis	IHF
groSL	CRP	IHF	rpoH
grpE	CRP	rpoH	Fis
grxA	OxyR	CRP	FNR
grxB	ArcA	CRP	CpxR
grxC	CsgD	Fis	LexA
grxD	CRP	H-NS	HU
gshA	H-NS	CpxR	FNR
gsk	rpoH	Fis	H-NS
gsp	ArcA	FNR	H-NS
gspCDEFGHIJKLMO	H-NS	GntR	AgaR
gst	ArcA	IHF	CRP
guaBA	Fis	PurR	DnaA
guaC	ArgR	CpxR	FNR
gudPXD	AgaR	DeoR	EvgA
gyrA	CRP	Fis	Lrp
gyrB	CpxR	Fis	LexA
hcaEFCBD	RcsAB	HcaR	PhoB
hcaR	Fis	CRP	DnaA
hcaT	AgaR	ChbR	EvgA
hchA	CRP	FNR	Fis
hcp-hcr	NarP	AtoC	FlhDC
hdeAB-yhiD	MarA	GadE	TorR
hdeD	GadE	GadX	H-NS
helD	AgaR	DicA	DnaA
hemA-prfA-prmC	ArcA	FNR	IHF
hemB	rpoH	CpxR	Fis
hemCDXY	NagC	rpoH	AllS
hemH	OxyR	IclR	MetJ
hemL	PhoP	ArgR	CdaR
hemN	CpxR	ArgR	H-NS
hepA-rluA	PurR	CRP	IHF
hflD-purB	PurR	CRP	FNR
hinT-ycfLM-thiK-nagZ-ycfP	CpxR	PhoB	CRP
hipBA	CpxR	IHF	CsgD
hisLGDCBHAFI	IHF	CRP	Lrp
hisS	ArgR	CpxR	FNR
hmp	AgaR	AsnC	FNR
hns	Fis	GadX	H-NS
hofMNOP	AgaR	AlsR	ArgR
hofQ	AllS	CysB	DeoR
holC-valS	CpxR	IHF	rpoH
holD-rimI-yjjG	Fis	LexA	CpxR
holE	H-NS	CRP	Fis
hpt	CpxR	DgsA	Fis
hrpA	EnvY	GutM	GutR
hscBA-fdx-iscX	FNR	IHF	IscR
hscC	AgaR	DeoR	DgsA

hsdRMS	CRP	CpxR	Fis
hslJ	CysB	CRP	IHF
hslVU	CRP	rpoH	Fis
hspQ	H-NS	rpoH	CRP
htgA	rpoH	FNR	IHF
htpG	CRP	IHF	rpoH
htpX	IHF	rpoH	CRP
htrG-cca	CpxR	AllR	ArcA
htrL	CRP	ArcA	CpxR
hupA	CRP	Fis	CpxR
hupB	CRP	EnvY	Fis
hyaABCDEF	NarP	IscR	NarL
hybOABCDEFG	NarL	ArcA	NarP
hycABCDEFGHI	FhlA	ModE	IHF
hydN-hypF	FhlA	HyfR	LsrR
hyfABCDEFGHIJR-focB	FhlA	HyfR	AgaR
hypABCDE-fhlA	FhlA	IHF	FNR
hyuA	FadR	GntR	MngR
iaaA-gsiABCD	IHF	CRP	CysB
iap	CpxR	DnaA	FhlA
ibpAB	IHF	rpoH	CRP
icd	ArcA	CRP	FruR
iclR	CpxR	FadR	IclR
idi	CpxR	DeoR	FNR
idnDOTR	GntR	IdnR	NagC
idnK	GntR	DgsA	IdnR
ilvC	CRP	IHF	Lrp
ilvIH	Lrp	H-NS	ArgR
ilvLG_1G_2MEDA	Lrp	IHF	CRP
ilvY	CpxR	Fur	H-NS
imp-surA-pdxA-ksgA-apaGH	Fis	H-NS	LexA
inaA	MarA	Rob	BetI
infA	CRP	FNR	Fis
insA-1AB-1B-1	Fis	CRP	CpxR
insA-2AB-2B-2-afuBC	AgaR	AllR	AppY
insA-5AB-5B-5	AgaR	CRP	CpxR
insA-7	CpxR	Fis	H-NS
insC-1CD-1D-1	ArcA	CRP	CpxR
insC-2CD-2D-2	CRP	CpxR	DicA
insC-4CD-4D-4-ygeONM	FNR	ArcA	CRP
insC-5CD-5D-5-yqiGHI	CytR	GntR	IHF
insE-2EF-2F-2	CRP	FNR	ArcA
insE-4EF-4F-4	CRP	DicA	FNR
insG	CaiF	CpxR	MarA
insH-5	LexA	ArgR	CytR
insH-8	CRP	CytR	H-NS
insL-1	CRP	IHF	ArcA
insMI-3	CRP	FNR	H-NS
insN-1I-1O-1	BetI	FlhDC	NanR
insO-2-yjhWV	AgaR	AraC	AscG
intA	CRP	FlhDC	GntR
intB	AllS	ArcA	CRP
intD	CpxR	NarL	PhoB
intF	CRP	Cbl	EvgA
intS	GntR	IdnR	LsrR
intZ	AllR	ArcA	CRP
iraP	CRP	IHF	ArcA
iscRSUA	IscR	CRP	FNR
ispB	NagC	ArgR	CpxR

ispDF	CpxR	CysB	FlhDC
ispG	rpoH	ArgR	CpxR
ispU	CRP	Fis	H-NS
ivbL-ilvBN	CRP	IHF	FNR
ivy	CRP	IHF	FNR
katE	CRP	IHF	H-NS
katG	FNR	OxyR	GadE
kbaZ-agaVWA	AgaR	EvgA	AllR
kbl-tdh	Lrp	CRP	Fis
kch	CRP	CpxR	DicA
kdgK	FruR	ArcA	CRP
kdgR	CRP	CpxR	FNR
kdgT	ArcA	CRP	CpxR
kdpDE	ArgR	CaiF	CpxR
kdpFABC	ModE	IHF	NtrC
kdsC-yrbK	CpxR	Fis	H-NS
kduD	FadR	FruR	HU
kduI	FadR	FruR	Fur
kefFC	AgaR	AtoC	DgsA
kefGB-yheV	PhoB	AscG	Cbl
kgtP	ArcA	CRP	FNR
kilR-ydaE	CsgD	FlhDC	GntR
kptA	CpxR	CytR	DeoR
kup	AsnC	CpxR	FadR
lacZYA	CRP	H-NS	Fis
ldcA	FNR	Fis	FruR
ldcC	NtrC	AppY	Lrp
ldhA	IHF	rpoH	CRP
lepAB	FNR	Fis	SoxS
leuE	ArgR	CRP	CysB
leuLABCD	CRP	Lrp	IHF
leuO	CpxR	CytR	GntR
leuS-rlpB-holA-nadD-cobC	FNR	ModE	PhoP
lexA-dinF	LexA	CRP	ArcA
lgt-thyA	CpxR	rpoH	Fis
ligA-ypeB	PhoB	FlhDC	GntR
ligT	ArgP	AscG	CaiF
lipA	CRP	FNR	FruR
lit	CpxR	CRP	CaiF
livJ	CRP	IHF	Lrp
livKHMGF	Lrp	IHF	CRP
lldPRD	ArcA	PdhR	CRP
lolA-rarA	Fis	H-NS	LexA
lolB-ispE-prs	CpxR	CusR	CynR
lolCDE	PhoP	CpxR	NarL
lomR_2-stfR-tfaR	ArgR	AllR	CpxR
lpcA	CRP	CpxR	Fis
lpp	CpxR	CRP	FNR
lptAB-rpoN-hpf-ptsN-yhbJ-npr	rpoH	CpxR	Lrp
lpxL	PhoB	ArgR	FruR
lpxM	EvgA	H-NS	HU
lpxP	CRP	FNR	Fis
lrhA	CRP	FNR	GntR
lsrACDBFG	CRP	FNR	LsrR
lsrK	CRP	FNR	Fis
lsrR	CRP	ArcA	FNR
luxS	Fis	CpxR	FruR
lysA	Lrp	ArgR	CRP
lysC	CRP	Lrp	ArgR

lysP	CRP	ArcA	FNR
lysU	FNR	Lrp	CRP
maa	CRP	CpxR	Fis
macAB	rpoH	ChbR	CpxR
maeA	FruR	Fis	GalR
maeB	CRP	IHF	ArcA
mak	CpxR	H-NS	LsrR
malEFG	CRP	MalT	FNR
malI	AraC	AscG	GatR
malK-lamB-malM	CRP	MalT	FNR
malPQ	FNR	MalT	CRP
malT	CRP	DgsA	Lrp
malXY	CRP	FlhDC	IHF
manA	SoxS	FruR	MarA
manXYZ	CRP	DgsA	NagC
maoC	ArgR	FhlA	MngR
map-glnD-dapD	CpxR	CRP	FNR
marC	Cbl	CdaR	MetR
marRAB	MarA	Rob	SoxS
matA	FNR	IHF	LexA
matB	CRP	H-NS	IHF
matC-yagXWV	AllR	FlhDC	NagC
mcrA	ArgR	AscG	FlhDC
mcrBC	CRP	CpxR	Fis
mdh	ArcA	CRP	FlhDC
mdlAB	AtoC	BetI	CaiF
mdoC	ArcA	CytR	Fis
mdoGH	Fis	FNR	IHF
mdtABCD-baeSR	BaeR	CpxR	NagC
mdtG	SoxS	CdaR	CytR
mdtH	AgaR	CsgD	CytR
mdtJI	CRP	Fis	IHF
mdtK	Rob	SoxS	CpxR
mdtM-yjiN	BaeR	CsgD	FhlA
mdtNOP	AgaR	AllR	CsgD
mdtQ	CusR	FlhDC	GntR
melAB	CRP	Fis	H-NS
melR	CRP	FNR	Fis
menA-rraA	Fis	PhoB	AgaR
menFD-yfbB-menBCE	CpxR	FNR	FhlA
metA	MetJ	MetR	rpoH
metBL	MetJ	PhoP	AllR
metC	MetJ	CRP	Nac
metE	CRP	MetJ	MetR
metF	CRP	MetJ	PhoP
metG	ArcA	FNR	Fis
metH	MetR	Fur	Lrp
metJ	Fur	IHF	Lrp
metK	CRP	FNR	MetJ
metNIQ	MetJ	CRP	IHF
metR	MetJ	CsgD	GadW
metY-yhbC-nusA-infB-rbfA-truB-rpsO-pnp	ArgR	Fis	CRP
mfd	FlhDC	H-NS	NagC
mglBAC	FlhDC	GalR	GalS
mgsA	CRP	FNR	GlpR
mgtA	CRP	FNR	PhoP
mhpABCDFE	MhpR	NagC	AllR
mhpR-lacI	CRP	NtrC	AraC
mhpT	GntR	NagC	OxyR

miaB	LexA	Fis	MalT
minCDE	H-NS	CRP	CpxR
mipA	CRP	FNR	ArcA
mlrA	CpxR	MarA	NtrC
mltA	ArgR	FlhDC	Fur
mltB	CpxR	FNR	Fis
mltD	CRP	FNR	Fis
mmuPM	ArgR	MetJ	OmpR
mngAB	AtoC	EnvY	FadR
mngR	AraC	ArgR	IclR
mnmA	CpxR	FNR	Fis
mnmC	Cbl	ChbR	IclR
mnmE	rpoH	GntR	NagC
mntH	CRP	Fur	IHF
mntR-ybiR	BetI	CusR	FadR
moaABCDE	ModE	CueR	FNR
mobAB	CpxR	AgaR	CRP
modABC	ModE	CRP	IHF
modEF	Fur	CRP	CpxR
moeAB	NarL	ArgR	DicA
mokB	ArcA	CRP	CpxR
mokC-hokC	AgaR	AscG	FNR
molR_2	RcsAB	LexA	NagC
molR_3-yehI	NagC	AllR	CusR
motAB-cheAW	CpxR	FlhDC	FNR
mpl	LexA	AgaR	CRP
mppA	FlhDC	CRP	FNR
mprA-emrAB	FNR	CRP	IHF
mqo	CRP	ArcA	FNR
mqsR-ygiT	FNR	IHF	CRP
mraZW-ftsLI-murEF-mraY-murD-ftsW-murGC-ddlB-ftsQAZ-lpxC	CpxR	FlhDC	RcsAB
mreBCD	FlhDC	Fis	CRP
mrp	FNR	IHF	NagC
mrr	AraC	CpxR	IclR
mscS	CRP	FNR	H-NS
msrA	CRP	FNR	FadR
msrB	ArcA	CRP	FNR
msyB	IHF	CRP	FNR
mtfA	FNR	CRP	CpxR
mtlADR	Fis	FruR	CRP
mtn-btuF-yadS	DcuR	EnvY	EvgA
mtr	CaiF	CdaR	FhlA
mug	CpxR	FNR	FlhDC
murA	FNR	CRP	CpxR
murB-birA	H-NS	Fis	FlhDC
murQP-yfeW	AgaR	PhoB	AllR
mutH	CsgD	HyfR	MetR
mutS	AppY	AsnC	FlhDC
mutY-yggX-mltC-nupG	CpxR	CytR	Fis
nac	Nac	NtrC	ArcA
nadA-pnuC	CRP	IHF	FNR
nadB	CRP	IHF	ModE
nadC	HU	LexA	PhoB
nadE	CpxR	CRP	FruR
nagBACD	NagC	PhoP	CRP
nagE	CRP	NagC	ArcA
nagK-cobB	LexA	MarA	Rob
nanATEK-yhcH	NanR	CRP	FNR
nanCM	NagC	NanR	CRP

nanR	SoxS	DicA	NagC
napFDAGHBC-ccmABCDEFGH	NarP	ModE	NarL
narGHJI	IHF	NarL	FNR
narK	FNR	IHF	NarL
narP	FlhDC	Fur	IHF
narQ	ArgR	GadX	HyfR
narU	RcsAB	ArgR	CsgD
narXL	ModE	CRP	CdaR
narZYWV	ArgR	NarP	RcsAB
ndh	ArcA	Fis	FNR
ndk	CRP	Fis	FNR
nemA	AraC	CRP	Cbl
nfo	MarA	Rob	SoxS
nfrBA	FlhDC	NtrC	ArgR
nfsB	MarA	CRP	CpxR
nhaAR	H-NS	Fis	BirA
nhaB	CpxR	Fis	H-NS
nhoA	NtrC	CRP	LexA
nikABCDER	NikR	NarL	FNR
nirBDC-cysG	NarP	FruR	NarL
nlpA	CRP	IHF	H-NS
nlpC	CpxR	Fis	IHF
nlpD-rpoS	CRP	ArcA	GadX
nlpI	FNR	CRP	Fis
nmpC	CRP	IHF	OmpR
nohA-ydfN-tfaQ	Fur	CRP	CpxR
norR	AgaR	AtoC	CysB
norVW	AgaR	CusR	GntR
nrdAB	DnaA	Fis	CRP
nrdDG	FNR	CRP	IHF
nrdHIEF	Fur	IHF	CRP
nrdR-ribDE-nusB-thiL-pgpA	Fis	CpxR	FruR
nrfABCDEFG	NarP	FlhDC	NarL
nsrR-rnr-rlmB	CpxR	OxyR	AgaR
nudB-yebC-ruvC	Fis	PurR	ArgR
nudE	CRP	Fis	FruR
nudF-yqiB-cpdA-yqiA-parE	CpxR	FlhDC	Fis
nudG	DeoR	FadR	ModE
nudI	CpxR	IHF	OxyR
nuoABCEFGHIJKLMN	Fis	NarL	IHF
nupC	CytR	Nac	CRP
nupX	AgaR	CsgD	FadR
ogrK	CpxR	Fis	H-NS
ompA	CRP	ArcA	CpxR
ompC	Lrp	OmpR	CpxR
ompF	Lrp	OmpR	CpxR
ompG	CRP	ModE	NagC
ompL	AgaR	Fis	H-NS
ompN	CpxR	FlhDC	GalR
ompR-envZ	ArgR	UxuR	CpxR
ompW	ArcA	FNR	CRP
ompX	CRP	FNR	IHF
oppABCDF	ArcA	Lrp	ModE
orn	ChbR	DeoR	GalR
osmB	CRP	RcsAB	H-NS
osmC	Lrp	RcsAB	H-NS
osmE	Fis	IHF	CRP
osmF-yehYXW	ArgR	GntR	MprA
osmY	CRP	IHF	Lrp

otsBA	IHF	SoxS	H-NS
oxc	AgaR	CpxR	GadE
oxyR	OxyR	FruR	Fur
paaABCDEFGHIJK	FlhDC	PaaX	ArgR
paaXY	FlhDC	CpxR	EvgA
pabB-nudL	CpxR	FlhDC	GutM
pabC-yceG-tmk-holB-ycfH	Fis	CpxR	LexA
pagB	LacI	MarA	MarR
pagP	PhoB	PhoP	ArgR
panBC	CpxR	FNR	CRP
panD	CRP	CpxR	Fis
panE-yajL	CpxR	Fur	MarA
parC	ArgR	CpxR	FNR
pck	CRP	FruR	FNR
pcnB-folK	FNR	CRP	CysB
pdhR-aceEF-lpd	FNR	ArcA	CRP
pdxB-usg-truA-dedA	Fis	HU	LexA
pdxH-tyrS-pdxY	FNR	FruR	rpoH
pdxK	SoxS	CaiF	Nac
pepA	CRP	CpxR	FNR
pepD	CsgD	FNR	Fis
pepE	Cbl	CsgD	FNR
pepN	CRP	CpxR	Fis
pepQ-yigZ-trkH-hemG	ArgR	CpxR	NagC
pepT	ArcA	CRP	FNR
perR	AscG	DsdC	FlhDC
pfkA	FruR	FNR	Fis
pfkB	CRP	FNR	IHF
pflA	CRP	FNR	Fis
pflDC	AgaR	GntR	AllR
pgaABCD	CpxR	NhaR	CRP
pgi	CRP	FNR	Fis
pgl	IHF	FNR	H-NS
pgpB	HyfR	LexA	NikR
pgsA	AraC	H-NS	IclR
pheLA	CRP	IHF	FNR
pheP	DgsA	FadR	NtrC
phnCDEEFGHIJKLMNOP	PhoB	AgaR	AllR
phoA-psiF	PhoB	FNR	CRP
phoBR	PhoB	DhaR	FNR
phoE	PhoB	AllR	ArgR
phoH	CRP	PhoB	Fis
phoPQ	PhoP	FNR	PhoB
pinR	CRP	H-NS	FNR
pitA	Fis	ArgR	FNR
pldA	rpoH	ArgR	FlhDC
pldB-yigL	CpxR	SoxS	FadR
plsB	CpxR	Fis	FlhDC
plsC	ArgR	CaiF	CytR
pmbA	ArgR	FlhDC	LexA
pmrD	CRP	FNR	FlhDC
pncB	CRP	CpxR	Fis
pntAB	CRP	Lrp	IHF
polA	CpxR	DnaA	H-NS
polB	LexA	ArcA	CRP
potABCD	CRP	Fis	FNR
potFGHI	NtrC	ArgR	Nac
poxA	ArgR	CysB	HU
poxB-ltaE-ybjT	CpxR	MarA	OxyR

ppa	CRP	Fis	CpxR
ppc	CRP	IHF	FNR
ppdAB-ygdB-ppdC-recC	AgaR	Cbl	GntR
ppdD-hofBC	rpoH	CsgD	DeoR
pphA	rpoH	ArgR	OxyR
pphB	CpxR	LexA	NagC
ppiA	CpxR	CytR	Fis
ppiB-lpxH	CpxR	Fis	CRP
ppiC	CpxR	FNR	GadE
ppiD	CpxR	rpoH	HU
ppk-ppx	CpxR	Lrp	DhaR
pppA	Fis	FruR	IscR
pps	CRP	FruR	ArcA
pptA	CRP	FNR	FruR
pqiAB	MarA	SoxS	Fis
prfC	Fis	CRP	FNR
priA	ChbR	DcuR	FadR
priC-ybaM	Cbl	AgaR	LsrR
prkB	AgaR	ExuR	KdpE
prlC-yhiQ	rpoH	CRP	Fis
prmB-aroC-mepA-yfcAML	CpxR	FlhDC	rpoH
proBA	rpoH	AllS	AraC
proC	CytR	FucR	LexA
proP	CRP	Fis	ArcA
proQ-prc	Fis	CRP	ExuR
proS	CRP	CpxR	FNR
proVWX	H-NS	CRP	ArcA
prpBCDE	CRP	Fis	PrpR
prpR	AllR	NtrC	PhoB
psd-yjeP	CpxR	AsnC	FadR
pspABCDE	PspF	IHF	CRP
pspF	PspF	DicA	LrhA
psrN-alx	ArcA	CRP	CpxR
pssA	ArcA	CRP	Fis
pstSCAB-phoU	PhoB	FNR	IHF
pth-ychF	Fis	CRP	Lrp
ptrA-recBD	Fis	AcrR	AsnC
ptrB	NagC	BaeR	CpxR
ptsA-fsaB-gldA	NarL	FlhDC	AllS
ptsG	ArcA	DgsA	CRP
ptsHI-crr	FruR	CRP	DgsA
purA	GadE	MarA	PurR
purC	CRP	PurR	FNR
purEK	PurR	PhoP	Lrp
purHD	PhoP	PurR	FNR
purL	PhoP	PurR	CRP
purR	Fur	Lrp	PhoB
purT	CRP	FNR	IHF
purU	ArcA	CRP	FNR
putP	CRP	IHF	FNR
puuA	IHF	CRP	FNR
puuCBE	IHF	FNR	NtrC
puuDR	NtrC	IHF	PhoB
puuP	CRP	CpxR	Fur
pykA	NarL	FNR	Fur
pykF	CRP	FruR	Fis
pyrC	PurR	ArgR	CRP
pyrD	FNR	IHF	PurR
pyrG-eno	FNR	ArcA	CRP

pyrH	FNR	ArgR	CRP
pyrLBI	IHF	FNR	PurR
qmcA-ybbJ	CpxR	Fis	PhoB
qor	CRP	FNR	Fis
qseBC	QseB	CysB	NarL
queA	Fis	ArcA	CRP
queC	GntR	HU	MetJ
racC	AraC	CdaR	CpxR
racR	CRP	FNR	Fis
raiA	CRP	IHF	FNR
rarD	DhaR	GntR	NarP
ravA-viaA	FruR	BaeR	CpxR
rbn	FhlA	GlpR	GntR
rbsDACBKR	CRP	RbsR	FNR
rcnA	FlhDC	GntR	MetR
rcnR	AscG	Cbl	NagC
rcsA	GadE	RcsAB	FlhDC
rcsC	PhoB	PhoP	H-NS
rcsDB	FlhDC	CpxR	ArgR
rcsF-yaeB	Fis	CRP	CpxR
rdgC	CpxR	FNR	Fur
rdoA-dsbA	CpxR	FNR	H-NS
recAX	LexA	ArcA	CRP
recET-lar-ydaCQ-intR	rpoH	AllR	ArgR
recN	ArcA	LexA	CRP
recQ	FlhDC	LexA	OmpR
relA-chpRA-mazG	H-NS	CpxR	Fis
relBE-hokD	CRP	IHF	FNR
rep	CRP	Fis	IHF
rfaDFCL	rpoH	CpxR	Fis
rfaE	rpoH	ArgR	DeoR
rfaH	Fis	FruR	GadX
rfaQGPSBIJYZ-waaU	rpoH	CpxR	CRP
rfbBDACX	CRP	IHF	FNR
rfe-wzzE-rffEDGHCA-wzxE-rffT-wzyE-rffM	FlhDC	Fis	CpxR
rhaBAD	NagC	AgaR	AllR
rhaSR	AgaR	Cbl	FhlA
rhaT	AgaR	CysB	FhlA
rhlB	CpxR	ArgR	FNR
rhlE	CRP	Fis	FlhDC
rhoL-rho	CRP	CpxR	Fis
rhsB	CpxR	Fis	H-NS
rhsC-ybfB	Cbl	CpxR	FNR
rhsD-ybbC-ylbH-ybbD	CpxR	Lrp	PhoB
rhsE-ydcD	CRP	CpxR	FNR
rhtA	ExuR	FadR	IdnR
rhtB	AgaR	ArgR	CdaR
rhtC	AgaR	FlhDC	FruR
ribA	SoxS	DeoR	FNR
ribB	CRP	FNR	FruR
ribF-ileS-lspA-fkpB-ispH	ArgR	CpxR	GadE
rihA	CRP	FNR	FlhDC
rihC	CRP	FNR	GalR
rimJ	CpxR	CRP	FNR
rimL	CRP	DgsA	DicA
rlmF	ArgR	CysB	FadR
rlmG	CRP	CpxR	FadR
rlmL-uup	Fis	FNR	H-NS
rlmN	Fis	rpoH	ArcA

rluB	CytR	DeoR	Fis
rluC	Fis	Fur	HU
rluD-yfiH	ArgR	BaeR	CaiF
rluE-nudJ	PurR	AsnC	Cbl
rluF	ArgR	MarA	PhoB
rmf	ArcA	CRP	Fis
rmuC	CRP	FNR	ArcA
rna	CpxR	Fis	PhoP
rnb	CRP	FNR	Fis
rnc-era-recO-pdxJ-acpS	FlhDC	CpxR	Fis
rnd	FadR	IclR	LexA
rnhA	ArgR	EvgA	IdnR
rnk	CpxR	DcuR	FlhDC
rnlA-yfjO	AllR	CRP	IHF
rnt-lhr	AsnC	FadR	GntR
rob	MarA	CpxR	Fur
rph-pyrE	FNR	Fis	RcsAB
rpiA	rpoH	CpxR	FadR
rpiR-alsBACE	AgaR	GntR	NagC
rplKAJL-rpoBC	FNR	Fis	ArcA
rplM-rpsI	ArcA	FNR	Fis
rplNXE-rpsNH-rplFR-rpsE-rpmD-rplO-secY-rpmJ	FNR	ArcA	Fis
rplU-rpmA	FNR	Fis	ArcA
rplY	CRP	Fis	FNR
rpmE	CRP	CpxR	FNR
rpmH-rnpA	FNR	Fis	CRP
rpoE-rseABC	CpxR	FNR	CRP
rpoH	CytR	DnaA	IHF
rpoZ-spoT-trmH-recG	rpoH	H-NS	CRP
rppH-ptsP	CpxR	BaeR	EvgA
rpsB-tsf	FNR	Fis	ArcA
rpsF-priB-rpsR-rplI	FNR	ArcA	Fis
rpsJ-rplCDWB-rpsS-rplV-rpsC-rplP-rpmC-rpsQ	ArcA	FNR	Fis
rpsLG-fusA-tufA	FNR	Fis	ArcA
rpsMKD-rpoA-rplQ	FNR	Fis	ArcA
rpsP-rimM-trmD-rplS	ArcA	FNR	Fis
rpsT	FNR	Fis	CRP
rpsU-dnaG-rpoD	LexA	Fis	FNR
rraB	CRP	CytR	Fur
rrmA	FadR	NhaR	OmpR
rrmJ-hflB	rpoH	CRP	CpxR
rsd	ArcA	CRP	H-NS
rseP	Fis	ArgR	CpxR
rsgA	ArgR	CpxR	FlhDC
rsmB	CpxR	ArgR	FNR
rsmC	CpxR	CRP	LexA
rsmD-yhhL	ArgR	CpxR	FlhDC
rsmE-gshB	CpxR	SoxS	GntR
rsmF	PurR	Fis	GadE
rspAB	CpxR	rpoH	Cbl
rssAB	NtrC	DicA	FhlA
rstAB	PhoP	FNR	IHF
rsuA	CpxR	FNR	IHF
rtcBA	MngR	NagC	NarP
rtn	FruR	GadE	H-NS
rumA	DcuR	FlhDC	MalT
rutABCDEFG	PhoP	ArcA	NtrC
rutR	AllS	DhaR	MarA
ruvAB	LexA	CRP	CdaR

sanA-yeiS	FlhDC	GntR	IdnR
sapABCDF	LexA	SoxS	AllR
sbcB	Fis	Fur	H-NS
sbcDC	AgaR	AllR	AsnC
sbmA-yaiW	CytR	FlhDC	IscR
sbmC	ArcA	CRP	FNR
sbp	IHF	CRP	CysB
scpA-argK-scpBC	AgaR	CsgD	EvgA
sdaA	CRP	Lrp	IHF
sdaCB	CRP	ArcA	IHF
sdhCDAB--sucABCD	Fur	ArcA	IHF
sdiA	CRP	FNR	Fis
secB-gpsA	rpoH	NagC	CRP
secE-nusG	Fis	ArgR	CRP
secG	Fis	ArgR	CRP
secMA-mutT	ModE	NarL	BaeR
selAB	NarL	NarP	CpxR
seqA-pgm	HU	PhoP	GadE
serA	CRP	Lrp	Nac
serB-radA-nadR	CpxR	GadW	PhoB
serC-aroA	CRP	Lrp	IHF
serS	FNR	ArcA	CRP
setB	CsgD	GalR	HU
sfmA	AgaR	CpxR	CsgD
sfmCD	ArgR	AtoC	CpxR
sfmHF	AcrR	Cbl	CsgD
sfsA	H-NS	CRP	FNR
sgcXBCQAER	GntR	FadR	CdaR
sgrR-sroA-tbpA-thiPQ	CpxR	AllR	ModE
sgrST-setA	CsgD	Fur	GlpR
shiA	CRP	FNR	GadX
sieB	CRP	CpxR	CytR
sirA	FNR	Fur	GadE
sixA	CpxR	Fur	H-NS
slp-dctR	H-NS	MarA	ArcA
slt	CpxR	DicA	ExuR
slyA	CpxR	FruR	Fur
slyB	Fis	PhoP	FNR
smg	CRP	CpxR	Fis
smpA	ArcA	CRP	CpxR
smpB	FNR	Fis	FruR
smtA-mukFEB	H-NS	CpxR	SoxS
sodA	MarA	Rob	SoxS
sodB	Fur	H-NS	IHF
sodC	Fis	CRP	Lrp
sohA-yhaV	CRP	CpxR	Fis
sohB	CysB	LexA	OxyR
solA-yceO	CpxR	PhoB	RcsAB
soxR	FNR	Fis	MetJ
soxS	SoxS	CRP	IHF
speAB	PurR	CpxR	FNR
speC	BetI	CRP	GntR
speF-potE	CpxR	CsgD	AllR
sppA	LexA	MarA	NagC
spr	CRP	IHF	H-NS
spy	BaeR	CRP	CpxR
srlAEBD-gutM-srlR-gutQ	GutM	GutR	CRP
srmB	Fis	CRP	FNR
ssb	LexA	CpxR	NhaR

sscR	CpxR	DeoR	Fis
sseA	CRP	FNR	GlpR
sseB	ArcA	CdaR	DnaA
sspAB	LexA	CpxR	FNR
sstT	IHF	ArcA	CRP
ssuEADCB	Cbl	NagC	ArgR
sthA	ArcA	CRP	FNR
sufABCDSE	OxyR	Fur	IscR
sugE	CpxR	CsiR	DeoR
suhB	CRP	FNR	Fis
sulA	ArcA	CRP	LexA
surE-pcm	ArgR	RcsAB	AcrR
sxy	CsgD	DicA	FhlA
syd	ArgR	CpxR	FlhDC
symE	CaiF	CpxR	LacI
tag-yiaC	CpxR	FhlA	ModE
talA	SoxS	Fis	H-NS
talB	FNR	ArcA	CRP
tam	NtrC	CRP	FNR
tar-tap-cheRBYZ	CpxR	FNR	FlhDC
tas	ArgR	CRP	H-NS
tatABCD	ArgR	FNR	Fis
tatE	CRP	CpxR	HU
tauABCD	CysB	Cbl	CpxR
tdcABCDEFG	IHF	TdcA	CpxR
tdcR	AgaR	CRP	Fis
tdk	CRP	FNR	Fis
tehAB	AscG	CRP	FNR
tesA-ybbO	AppY	AraC	ArgR
tesB	PhoB	Cbl	CueR
tfaD	FNR	Fur	CRP
tgt-yajC-secDF	Fis	CRP	FNR
thiCEFSGH	CRP	IHF	Lrp
thiI	Fis	CpxR	IHF
thiMD	CpxR	H-NS	Lrp
thrLABC	IHF	CRP	Lrp
thrS-infC-rpmI-rplT-pheMST-ihfA	FNR	CRP	Fis
thrU-tyrU-glyT-thrT-tufB	ArcA	FNR	Fis
tig	FNR	Fis	ArcA
tilS	AgaR	DicA	HyfR
tktA	ArcA	CRP	FNR
tktB	SoxS	H-NS	IHF
tldD	CpxR	ExuR	Fis
tnaCAB	TorR	CRP	ArcA
tolB-pal-ybgF	CpxR	FNR	Fis
tolC-ygiABC	CpxR	Fis	rpoH
tonB	Fur	IHF	CRP
topA	Fis	rpoH	FlhDC
torCAD	NagC	NarL	NarP
torR	DgsA	NtrC	PhoB
torS	Rob	Cbl	CdaR
torT	CsgD	DicA	ExuR
torYZ	NagC	Rob	CaiF
tpiA	CRP	FNR	Fis
tppB	OmpR	ArgR	CRP
tpx	FNR	ArcA	FruR
tqsA	FlhDC	CpxR	RcsAB
treA	NtrC	FNR	Fis
treBC	ArcA	CRP	IHF

treF	NtrC	Ada	FNR
treR	PhoP	DeoR	HcaR
trg	FNR	CRP	CpxR
trkA	ArgR	CpxR	Fis
trkG	ArgP	CRP	CpxR
trmA	Fis	rpoH	CRP
trmI-yggL	FNR	Fis	AllS
trmJ	ArgR	CpxR	Fis
trpH-yciOQ	PhoB	FlhDC	LexA
trpLEDCBA	TrpR	IHF	Lrp
trpR	TrpR	ChbR	GalR
truD	CpxR	CusR	GadE
trxA	CRP	CpxR	Fis
trxB	Fis	FNR	H-NS
trxC	OxyR	ArcA	Fur
tsgA	CRP	Fis	FNR
tsr	CpxR	FlhDC	FNR
tsx	CRP	CytR	ArcA
ttcA	CRP	FNR	HU
ttdABT	AgaR	ChbR	BaeR
ttdR	CysB	FhlA	FlhDC
tus	CpxR	Fis	FlhDC
tusE	BaeR	LexA	NhaR
tynA	RcsAB	CusR	FlhDC
typA	FNR	Fis	ArcA
tyrB	TyrR	CpxR	FruR
tyrP	HU	TyrR	Fur
tyrTV-tpr	CRP	FNR	Fis
ubiCA	NarL	ArcA	IHF
ubiD	CpxR	FNR	FruR
ubiE-yigP-ubiB	ArgR	CpxR	Fis
ubiF	GadX	LexA	NarP
ubiG	AscG	Cbl	CpxR
ucpA	CRP	FNR	GntR
udk-dcd	FNR	SoxS	Fis
udp	CRP	CytR	FNR
ugd	CRP	CpxR	FlhDC
ugpBAECQ	PhoB	NtrC	Fis
uhpABC	FhlA	AgaR	CRP
uhpT	CRP	CaiF	FNR
uidABC	UxuR	CRP	MarA
uidR	Nac	NagC	NarL
ulaABCDEF	IdnR	UlaR	AgaR
ulaG	AgaR	FadR	GntR
umuDC	LexA	CRP	ArcA
ung	CpxR	H-NS	LexA
upp-uraA	FNR	IHF	CRP
ushA	FadR	CueR	FNR
uspA	CRP	FadR	Fis
uspC	AraC	ArgP	AscG
uspE	ArcA	CRP	FNR
uspF	CRP	DnaA	FNR
uspG	rpoH	CRP	FNR
uvrA	ArcA	LexA	CRP
uvrB	ArcA	LexA	CRP
uvrD	LexA	FlhDC	H-NS
uvrYC	LexA	CpxR	rpoH
uxaB	CRP	ExuR	FlhDC
uxaCA	ExuR	FNR	AgaR

uxuAB	UxuR	ExuR	CRP
uxuR	ExuR	BirA	FruR
vacJ	FruR	CpxR	GadX
ves	CpxR	FlhDC	FNR
waaA-coaD	AgaR	ArgR	ExuR
wbbIJK	CRP	IHF	ArcA
wcaCDEF-gmd-fcl-gmm-wcaI-cpsBG-wcaJ-wzxC	AgaR	ArgR	FhlA
wcaKLM	AtoC	NagC	RcsAB
wrbA-yccJ	CRP	IHF	Fis
wza-wzb-wzc-wcaAB	RcsAB	AllR	FlhDC
xapAB	GntR	RcsAB	ArgR
xapR	FlhDC	Fur	GntR
xdhABC	GntR	AgaR	AllR
xerD-dsbC-recJ-prfB-lysS	CpxR	rpoH	FNR
xni	GntR	DhaR	IclR
xseA	CRP	CpxR	Fis
xseB-ispA-dxs-yajO	H-NS	Fis	CRP
xthA	CpxR	FNR	IHF
xylAB	Fis	XylR	AgaR
xylE	BaeR	CpxR	FNR
xylFGHR	Fis	CRP	GntR
yaaA	AscG	IscR	NanR
yaaH	BaeR	Fur	MalT
yaaJ	CaiF	CsgD	FlhDC
yaaU	FadR	NagC	NarP
yaaW	AllR	GalR	GalS
yabPQ	BaeR	CRP	FNR
yacC-speED	CRP	Fis	IHF
yacH	AgaR	BaeR	Cbl
yacL	CRP	CpxR	Fur
yadE	AgaR	CdaR	FlhDC
yadGH	CpxR	Lrp	ModE
yadI	CytR	CdaR	FNR
yadMLKC	IHF	rpoH	CRP
yadN	AraC	AscG	CaiF
yaeF	AllR	LexA	MarA
yaeH	CRP	FNR	IHF
yaeI	ChbR	CytR	DnaA
yaeQJ-nlpE	CpxR	Cbl	PhoP
yaeT-hlpA-lpxD-fabZ-lpxAB-rnhB-dnaE	CpxR	GadE	Fis
yafC	ArgR	CpxR	DicA
yafDE	Fis	CpxR	CRP
yafJ	CpxR	FNR	H-NS
yafK	Fis	IHF	CRP
yafM	ArgR	CpxR	CynR
yafNOP	LexA	CRP	Cbl
yafS	ArgR	DhaR	GntR
yafT	AraC	CsgD	Fur
yafV	Cbl	FadR	MarA
yafY-ykfBF-yafX-ykfGH-yafW-ykfI	FlhDC	Cbl	CRP
yagAB-ykgN	AraC	CaiF	CsgD
yagEF	ArcA	CRP	Fis
yagGH	AllR	ArgR	FucR
yagI	AraC	ArgR	AscG
yagJ	FlhDC	GntR	HU
yagML	CpxR	IHF	NhaR
yagN	CRP	ArcA	IHF
yagP	CsgD	LexA	MetR
yagTSRQ	ArgR	FlhDC	MprA

yagU	CRP	IHF	FNR
yahA	CRP	Fis	Fur
yahB	AcrR	DgsA	Fis
yahC	Cbl	EnvY	EvgA
yahDEFG	PhoB	AgaR	NtrC
yahIJ	FNR	ModE	NagC
yahK	GadE	GadX	H-NS
yahL	CpxR	Fur	GntR
yahM	CRP	CueR	Fis
yahO	CRP	FNR	Fis
yaiE	CRP	DcuR	Fis
yaiF	DicA	Fur	LexA
yaiL	CpxR	GadE	LexA
yaiP	AllR	CusR	DicA
yaiT	Fur	NarL	DicA
yaiU	CytR	GntR	LexA
yaiY	CpxR	FlhDC	LexA
yaiZ	CRP	CpxR	DeoR
yajD	CpxR	Fur	HcaR
yajG	CRP	Fis	CpxR
yajQ	CRP	CpxR	Fis
yajR	BetI	MelR	PspF
ybaA	CRP	CdaR	Fis
ybaB-recR	CpxR	CytR	DeoR
ybaE	ArgR	DgsA	FlhDC
ybaJ-hha	CpxR	CRP	FNR
ybaK	AscG	CaiF	Cbl
ybaL	HU	Rob	SoxS
ybaO	CpxR	DicA	HcaR
ybaP	SoxS	Cbl	CpxR
ybaQ	CpxR	CRP	Fur
ybaST	GadX	H-NS	MarA
ybaW	DicA	GntR	HU
ybaY	Fis	IHF	CRP
ybbAP	ArgR	GalR	GalS
ybbB	ExuR	GntR	MetJ
ybbLM	CRP	CpxR	GlpR
ybbN	rpoH	CRP	GadX
ybcH	ArgR	IscR	NtrC
ybcI	AraC	CsgD	DhaR
ybcK	AgaR	CpxR	FNR
ybcLM	PhoB	CpxR	ArgR
ybcV	Ada	BetI	CRP
ybdG	AraC	IclR	MprA
ybdH	Fur	MetJ	ModE
ybdK	CytR	Fis	NtrC
ybdL	rpoH	ArgR	CRP
ybdM	AllR	BetI	GalR
ybdN	CpxR	NagC	NanR
ybdO	CpxR	CytR	IHF
ybdR	RcsAB	CpxR	FhlA
ybeBA-mrdAB-rlpA	Fis	CpxR	FNR
ybeD-lipB	rpoH	CRP	CpxR
ybeF	CytR	LexA	MarA
ybeHM	ArgR	Fur	GntR
ybeL	H-NS	CRP	FNR
ybeQ	CpxR	DgsA	LexA
ybeR-djlB	CpxR	CusR	AgaR
ybeU-djlC	AgaR	ArgR	CaiF

ybeZYX-lnt	CRP	rpoH	Fis
ybfA	ArcA	CRP	DcuR
ybfD	AgaR	ArgR	CRP
ybfE	ArcA	CRP	Fis
ybfF	IclR	NtrC	PhoB
ybfL	CRP	CpxR	CsgD
ybfM	RcsAB	CpxR	Lrp
ybfN	LexA	NagC	NtrC
ybgA-phr	Ada	CynR	EvgA
ybgC-tolQRA	Fis	ArgR	CpxR
ybgD	CRP	CusR	FNR
ybgE	FNR	FruR	ArcA
ybgH	ArgR	Cbl	ChbR
ybgIJKL-nei	Lrp	rpoH	IHF
ybgQPO	RcsAB	CdaR	Fur
ybgS	NtrC	ArcA	IHF
ybhA	H-NS	CRP	Fur
ybhC	CRP	CpxR	FNR
ybhD	ArgR	CRP	Fur
ybhH	AgaR	BetI	CRP
ybhK	LexA	ArcA	H-NS
ybhL	CRP	CdaR	FNR
ybhM	AgaR	CpxR	CsgD
ybhPON	NtrC	FhlA	MarA
ybhQ	CRP	CdaR	CpxR
ybiC	CRP	FNR	IHF
ybiH-ybhGFSR	FNR	rpoH	CRP
ybiI	CRP	Fur	IHF
ybiJ	Cbl	FNR	IHF
ybiM	CpxR	CsgD	DeoR
ybiP	Fur	Fis	FlhDC
ybiT	rpoH	FNR	FadR
ybiU	ArcA	CRP	FNR
ybiV	CRP	FNR	HU
ybiYW	AgaR	AtoC	CaiF
ybjC-nfsA-rimK-ybjN	SoxS	Fis	AgaR
ybjD	FadR	Fur	H-NS
ybjE	AppY	Cbl	CpxR
ybjG	PhoP	ArcA	IHF
ybjH	Cbl	FadR	GutM
ybjJI	SoxS	AgaR	AraC
ybjK	AgaR	DgsA	ExuR
ybjL	CpxR	CytR	FadR
ybjM	ArcA	CRP	FNR
ybjO-rumB	PurR	ExuR	Fis
ybjP	IHF	FNR	Fis
ybjQ-amiD	AppY	rpoH	Cbl
ybjS	FlhDC	FruR	H-NS
ybjX	CRP	FNR	H-NS
ycaC	BaeR	H-NS	FNR
ycaD	DicA	GntR	ModE
ycaI-msbA-lpxK-ycaQ	ArcA	AscG	CRP
ycaM	ArgR	CpxR	FhlA
ycaN	ArgR	CdaR	CpxR
ycaO	Fis	CRP	IHF
ycaP	Ada	Fis	MarA
ycaR-kdsB	AllR	ArcA	CpxR
ycbB	CpxR	FNR	Fis
ycbC	DeoR	DicA	GlpR

ycbG	Fis	FruR	IclR
ycbJ	CRP	FNR	FhlA
ycbKL	CRP	H-NS	IHF
ycbRSTUVF	NagC	RcsAB	UxuR
ycbW	CpxR	Fur	GlpR
ycbX	CpxR	Fur	H-NS
ycbZ	ArgR	Fis	FlhDC
yccA	CRP	CpxR	FNR
yccE	ArgR	CpxR	GalR
yccFS	CpxR	DeoR	DgsA
yccM	Fis	FlhDC	Fur
yccT	AscG	NagC	NanR
yccU	NtrC	AllS	CpxR
yccW	ArcA	FNR	Fis
yccX	BaeR	FlhDC	MtlR
ycdT	CpxR	H-NS	LexA
ycdXY	MprA	PhoP	Cbl
ycdZ	AraC	ArcA	CRP
yceA	CRP	ArcA	FNR
yceB	AllR	CaiF	FadR
yceD-rpmF-plsX-fabHDG-acpP-fabF	Fis	FNR	CRP
yceF	AppY	BaeR	Cbl
yceH	ArgP	CaiF	CpxR
yceJI	ArcA	CRP	FNR
yceK	ArgR	FNR	IHF
yceM	CpxR	DeoR	GntR
yceN	CpxR	FNR	FadR
yceQ	CpxR	FlhDC	ArgR
ycfD	ExuR	Fis	MtlR
ycfJ	FNR	Fis	IHF
ycfQ	CpxR	CytR	FNR
ycfS	CpxR	BaeR	CRP
ycgB	NtrC	CRP	FNR
ycgE	CRP	CpxR	FNR
ycgF	CRP	CpxR	FNR
ycgG	CpxR	Fis	FNR
ycgK	CRP	Fis	Fur
ycgL	ArgP	CRP	DnaA
ycgM	CpxR	CsgD	Fur
ycgN	PhoP	CpxR	Lrp
ycgR	CpxR	FlhDC	FNR
ycgV	ArgR	CytR	FlhDC
ycgX	AraC	CRP	Cbl
ycgZ	IHF	CRP	CpxR
ychE_2-yhcF	DnaA	FadR	FlhDC
ychH	CRP	CpxR	FNR
ychJ	CpxR	FNR	Fis
ychM	CRP	FNR	FruR
ychN	AllS	CRP	DgsA
ychO	GntR	NagC	NarP
ychQA-kdsA	CRP	FNR	Fis
ychS	Fis	FNR	FhlA
yciA	CRP	Fis	FlhDC
yciCB	ArcA	CRP	AraC
yciGFE	H-NS	FNR	CRP
yciI	CRP	IHF	Lrp
yciK-btuR	AscG	FlhDC	AraC
yciN	CpxR	FNR	GntR
yciSM-pyrF-yciH	Fis	Nac	CRP

yciW	CRP	IHF	CysB
yciZ-deoT	CpxR	H-NS	Lrp
ycjG	DgsA	FadR	Nac
ycjMNOPQRSTUV-ymjB	rpoH	GntR	NagC
ycjW	LexA	NarP	PurR
ycjXF-tyrR	rpoH	SoxS	TyrR
ycjY	CpxR	FlhDC	LexA
ycjZ	CdaR	CpxR	MalT
ydaGF	CpxR	CytR	Fis
ydaL	CRP	Fis	Lrp
ydaM	CRP	FNR	GntR
ydaN	ArcA	CRP	CpxR
ydaSTUVW-rzpR	GntR	ArgR	NagC
ydbA_1	CpxR	CusR	DgsA
ydbA_2	CRP	CpxR	CsgD
ydbC	ArgR	CpxR	NagC
ydbD	Cbl	CdaR	CpxR
ydbH-ynbE-ydbL	NarL	PaaX	PhoB
ydbK	CRP	CpxR	IHF
ydcF	CpxR	FlhDC	NtrC
ydcI	CRP	ArcA	FNR
ydcK	NtrC	CRP	FNR
ydcL	NtrC	CRP	EnvY
ydcN	AsnC	HcaR	MarA
ydcO	LexA	NtrC	PhoB
ydcP	CRP	CpxR	Fis
ydcR	ArgR	ExuR	NagC
ydcSTUV	NtrC	ArgR	Lrp
ydcW	ArgR	DeoR	DicA
ydcY	Rob	ModE	PhoB
yddAB	CRP	CpxR	Fur
yddE	DicA	IclR	LexA
yddG	CpxR	GadE	NarP
yddH	FlhDC	FruR	NagC
yddLKJ	CpxR	FNR	FlhDC
yddM	AraC	CRP	CpxR
yddV-dos	NtrC	AtoC	CaiF
yddW	RcsAB	ArgR	FruR
ydeA	ArcA	FNR	CRP
ydeE	HU	MalT	NagC
ydeH	CpxR	CRP	FNR
ydeM	Fis	CRP	CpxR
ydeN	CRP	FNR	Fis
ydeP	CRP	CpxR	H-NS
ydeQ	CpxR	FlhDC	PhoB
ydeTSR	CpxR	Fur	IHF
ydfAB-rzpQ	Fis	FlhDC	AllS
ydfG	ArcA	CRP	CytR
ydfH	CpxR	DicA	EnvY
ydfI	FNR	FlhDC	Fur
ydfJ	ArgR	CpxR	FlhDC
ydfUT	CpxR	FlhDC	GntR
ydfV	AgaR	ArgR	CpxR
ydfZ	ArcA	CRP	FNR
ydgA	Fis	FNR	IHF
ydgD	PhoB	ArgR	CpxR
ydgH	CpxR	FNR	H-NS
ydgI-folM	rpoH	FruR	NarL
ydgJ	GadX	LeuO	LexA

ydgK-rsxABCDGE-nth	Fis	FNR	CpxR
ydhB	MelR	MhpR	RcsAB
ydhC	DcuR	HU	IclR
ydhF	ArcA	CRP	CsiR
ydhIJK	rpoH	Fis	PhoP
ydhL	FlhDC	ArgR	NtrC
ydhM	CRP	ExuR	Fis
ydhO	SoxS	Fur	H-NS
ydhP	FruR	GntR	NtrC
ydhQ	CRP	FNR	IHF
ydhR	Fis	ArcA	CRP
ydhS	NtrC	CpxR	H-NS
ydhXUT	NagC	GntR	FhlA
ydhYVW	NarL	FNR	IHF
ydhZ	BaeR	CpxR	FadR
ydiA	CRP	FNR	Fis
ydiE	CRP	CytR	FhlA
ydiFO	RcsAB	ArgR	CpxR
ydiJIH	CRP	FNR	H-NS
ydiK	CsgD	HU	LexA
ydiL	CpxR	FlhDC	LexA
ydiM	CpxR	FlhDC	GntR
ydiNB-aroD	CpxR	RcsAB	rpoH
ydiP	CpxR	FNR	FlhDC
ydiQRST-fadK	NagC	GntR	UxuR
ydiU	IscR	CRP	Fur
ydiV	CpxR	PhoB	DicA
ydiY	CRP	FNR	IHF
ydiZ	CRP	ArcA	Fis
ydjA-selD-topB	rpoH	ArcA	FNR
ydjE	CpxR	CysB	FlhDC
ydjF	CpxR	Fis	FlhDC
ydjLKJIHG	CpxR	FlhDC	NagC
ydjM	CpxR	LexA	FlhDC
ydjN	IHF	CRP	CysB
ydjO	AraC	CpxR	CsgD
ydjXYZ-ynjABCD	NarL	IHF	FNR
yeaC	CRP	FNR	ArcA
yeaE	FlhDC	GntR	LeuO
yeaGH	Fis	NtrC	CRP
yeaI	CpxR	H-NS	IHF
yeaL	NagC	ChbR	PhoB
yeaM	FlhDC	ArgR	CpxR
yeaN	RcsAB	CytR	FadR
yeaO	Ada	CpxR	FlhDC
yeaP	CRP	CysB	FNR
yeaQ	FNR	Fis	IHF
yeaR-yoaG	FNR	IHF	NarP
yeaT	CRP	CpxR	CytR
yeaU	ArcA	CRP	IHF
yeaVW	NagC	GntR	CusR
yeaX	FadR	GntR	LexA
yeaZY	Fis	ArcA	BaeR
yebA	CpxR	Fis	FlhDC
yebB	CsgD	LexA	MetR
yebE	CpxR	ArcA	CRP
yebF	LexA	ArcA	CRP
yebG	LexA	ArcA	CRP
yebK	CRP	FNR	IHF

yebN	rpoH	CsgD	FruR
yebO	FNR	CRP	Fis
yebQ	CpxR	FlhDC	H-NS
yebR	CRP	CpxR	Fis
yebST	RcsAB	AgaR	AscG
yebV	H-NS	IHF	CRP
yebW	CpxR	FNR	Fur
yecA	PhoB	ArcA	CRP
yecDE	ArgR	CRP	CpxR
yecF	AraC	CaiF	CpxR
yecH	ArcA	FNR	GcvA
yecM	CaiF	CpxR	ModE
yecN-cmoAB	PhoB	PhoP	CRP
yecR	AraC	FNR	FlhDC
yecT	CdaR	CpxR	FlhDC
yedA	DgsA	IclR	ModE
yedD	AllR	FNR	IHF
yedEF	FNR	IHF	CRP
yedI	NtrC	FruR	PhoB
yedK	ArgR	DicA	HU
yedL	CRP	FNR	GadX
yedM	Cbl	FNR	FlhDC
yedP	Lrp	MarA	NtrC
yedQ	CpxR	ArgR	NtrC
yedRJ	PhoB	AllS	CRP
yedWV	CRP	CpxR	FNR
yedX	ArcA	CRP	FNR
yedYZ	CRP	FNR	FlhDC
yeeA	LexA	CRP	H-NS
yeeED	CRP	IHF	CysB
yeeF	CRP	ArcA	FNR
yeeN	CRP	ArcA	CpxR
yeeO	CytR	EnvY	NagC
yeeP	ArgR	CaiF	IdnR
yeeRSTUVW	ExuR	NarL	OxyR
yeeX	Fis	ArcA	CRP
yeeY	CpxR	FruR	Fur
yeeZ	CpxR	Fis	Fur
yefM-yoeB	CRP	CpxR	FNR
yegD	FadR	FNR	Fis
yegH	GatR	HcaR	IclR
yegLK	NagC	Rob	AtoC
yegP	H-NS	CRP	FNR
yegQ	Fis	CRP	FNR
yegS	CRP	DgsA	Lrp
yegTUV	RcsAB	Rob	UxuR
yehDCBA	AgaR	ArgP	ArgR
yehE	CRP	DgsA	Fis
yehLMPQ	AtoC	FadR	GntR
yehR	AraC	AscG	CdaR
yehS	CpxR	DhaR	FhlA
yehUT	CRP	IHF	Fis
yeiCN	GntR	AgaR	CRP
yeiE	BaeR	BirA	CpxR
yeiG	FruR	Fis	FlhDC
yeiH	Cbl	LrhA	MhpR
yeiI	AllR	CpxR	DeoR
yeiL	CsgD	AraC	MprA
yeiM	AgaR	AscG	HU

yeiP	FNR	Fis	Fur
yeiQ	CRP	ExuR	H-NS
yeiR-lpxT	AsnC	CRP	CpxR
yeiTA	CRP	FNR	IHF
yejABEF	Fur	CpxR	IHF
yejG	ArcA	CRP	FNR
yejH	AllR	HyfR	LexA
yejK	AsnC	CRP	Fis
yejLM	ArcA	ArgR	CRP
yejO	HyfR	NagC	PdhR
yfaATS_1S_2QP	AtoC	NagC	RcsAB
yfaD-ypaA	RcsAB	CsgD	FlhDC
yfaE	DnaA	AraC	ArgP
yfaH	CRP	Fis	GutM
yfaL	CaiF	CsgD	CytR
yfaVU	AllR	RcsAB	UxuR
yfaXW	RcsAB	FlhDC	GalR
yfaY	AsnC	HU	PhoB
yfaZ	CsgD	FadR	FhlA
yfbK	ArgR	CaiF	CsgD
yfbL	AgaR	CsgD	FucR
yfbM	AllR	ArgR	CpxR
yfbN	AgaR	ArgR	HU
yfbO	ArgR	CRP	CpxR
yfbP	AgaR	CpxR	LexA
yfbQ	CRP	CpxR	FNR
yfbUT	CRP	CpxR	CsgD
yfbV	rpoH	CpxR	FNR
yfcC	AgaR	ExuR	FucR
yfcD	IHF	CpxR	FNR
yfcE	Fis	CRP	FNR
yfcF	ArgR	BaeR	ModE
yfcG	HyfR	LrhA	NanR
yfcI	DicA	FhlA	HyfR
yfcUTSRQPO	NagC	RcsAB	Cbl
yfcZ	CRP	CadC	EvgA
yfdC	NtrC	CaiF	FlhDC
yfdE	CpxR	ModE	NagC
yfdF	CpxR	GadE	GalR
yfdGHI	CRP	IHF	Fur
yfdONMLK	RcsAB	ArgR	Cbl
yfdPQ	AgaR	ArgR	CusR
yfdRST	ArgR	rpoH	AgaR
yfdV	ArgR	CpxR	Fur
yfdX	AraC	ArgP	CpxR
yfdY	CRP	CaiF	Cbl
yfdZ	PhoB	FruR	PurR
yfeA	DicA	FhlA	GadX
yfeCD	CRP	FNR	NanR
yfeKS	CpxR	AtoC	CueR
yfeO	CpxR	H-NS	MarA
yfeR	CaiF	ChbR	FhlA
yfeT	AgaR	CadC	DgsA
yfeYX	CpxR	FlhDC	NarL
yffB-dapE-ypfN	PhoB	rpoH	ArgP
yffL	ArcA	CRP	HU
yffMN	Fur	IscR	NarL
yffOP	RcsAB	AllR	ExuR
yffS	ArcA	CRP	IHF

yfgA	rpoH	ArgR	CpxR
yfgCD	CpxR	CysB	AgaR
yfgF	AllR	Cbl	Fis
yfgG	CRP	CpxR	Fis
yfgHI	CsgD	AgaR	AscG
yfgL-der	Fis	ArgR	FruR
yfgO	CsiR	DeoR	RcsAB
yfhA	YiaJ	CaiF	FhlA
yfhB-tadA	CpxR	DicA	FadR
yfhD	Rob	BaeR	Fis
yfhG	CpxR	CusR	HyfR
yfhH	FhlA	GalR	GalS
yfhK	CusR	FadR	MtlR
yfhL	CRP	Cbl	ChbR
yfhM-pbpC	CaiF	Cbl	CynR
yfhR	AgaR	CaiF	NagC
yfiC	AlsR	AraC	Cbl
yfiD	ArcA	FNR	PdhR
yfiF	AgaR	GadX	MarA
yfiL	CaiF	Cbl	NtrC
yfiM	CpxR	CsgD	Fis
yfiO	CpxR	Fis	FlhDC
yfiPQ	BaeR	CRP	DicA
yfiRNB	CpxR	FNR	rpoH
yfjGF	CpxR	Fis	LexA
yfjLK	FlhDC	NarL	OmpR
yfjPQ	AllR	AlsR	ArgR
yfjR-ypjK-yfjST	FlhDC	AtoC	Cbl
yfjXY-ypjJ-yfjZ-ypjF	Cbl	AllR	AllS
ygaC	Fur	CpxR	IHF
ygaM	IHF	CRP	Fis
ygaU	CRP	Fis	H-NS
ygaVP	CysB	Ada	BaeR
ygaW	IHF	CRP	CpxR
ygaXY	BaeR	CaiF	Fis
ygaZH	FNR	Fis	CRP
ygbI	CaiF	CueR	GutM
ygbJK	FlhDC	AllS	ChbR
ygbLM	GntR	AgaR	Cbl
ygcB	ArgR	GntR	HU
ygcF	CpxR	FNR	Lrp
ygcLKJIH-ygbTF	FhlA	NtrC	ArgR
ygcNOP	CRP	GadE	MarA
ygcRQ	PhoB	AllR	AtoC
ygcS	PhoB	EvgA	FhlA
ygdE	BaeR	GcvA	Nac
ygdH	CRP	FNR	Lrp
ygdI	IHF	ArcA	CRP
ygdL	CaiF	CueR	FucR
ygeA	AraC	CRP	CaiF
ygeF	AgaR	AppY	Fis
ygeH	AllS	Fis	GntR
ygeLK	CusR	Fis	H-NS
ygeP	CRP	CdaR	CytR
ygeQ	ArgR	CRP	FNR
ygeR	CpxR	DicA	GlpR
ygeV	CRP	DgsA	FNR
ygeW	AtoC	Cbl	CpxR
ygeY	AgaR	AllR	CsgD

ygfA-rygC	CpxR	FadR	H-NS
ygfB-pepP-ubiH-visC	CpxR	LexA	rpoH
ygfF	Cbl	DgsA	FhlA
ygfI	FhlA	GadX	GntR
ygfJ	CRP	FNR	H-NS
ygfK-ssnA	AgaR	NagC	AcrR
ygfM-xdhD	AgaR	AcrR	AllS
ygfO-guaD-ygfQ	GntR	AcrR	AllR
ygfU	GntR	AllR	GalR
ygfYX	SoxS	CpxR	FadR
ygfZ	CpxR	FlhDC	H-NS
yggE	CRP	FNR	IHF
yggG	Lrp	MalT	MetR
yggI	CaiF	DicA	GadX
yggM	CusR	FlhDC	GntR
yggN	CpxR	FlhDC	FruR
yggR	CsgD	GalR	GalS
yggSTU-rdgB-yggW	rpoH	FlhDC	CpxR
yghA	H-NS	IHF	Lrp
yghB	CpxR	GalR	GalS
yghED	AllR	AscG	AsnC
yghF	rpoH	AllR	FadR
yghG	BirA	CRP	DicA
yghO	AcrR	AgaR	DnaA
yghQ	AgaR	CysB	ModE
yghSR	AtoC	GntR	MngR
yghT	AcrR	AgaR	CsgD
yghU	ArcA	H-NS	LexA
yghW	CpxR	FlhDC	IHF
yghZ	CRP	FNR	H-NS
ygiD	DeoR	CytR	DgsA
ygiF-glnE	ArgR	AraC	Cbl
ygiL	ArgP	CpxR	FlhDC
ygiN	HU	HcaR	PhoP
ygiS	FNR	Fis	GadE
ygiV	CpxR	CysB	MarA
ygiW	CRP	IHF	FNR
ygiZ	CpxR	Fis	H-NS
ygjG	Fis	IHF	NtrC
ygjH	AgaR	GutM	GutR
ygjI	FhlA	FlhDC	HyfR
ygjNM	BetI	CRP	Fis
ygjP	CpxR	DicA	IclR
ygjR	CRP	CpxR	CysB
ygjV	CdaR	CpxR	CsgD
yhaBC	Fis	H-NS	IHF
yhaH	BaeR	Cbl	EnvY
yhaI	AgaR	HyfR	NarL
yhaJ	CaiF	EnvY	FlhDC
yhaKL	CpxR	CynR	KdpE
yhaOM	CRP	CdaR	Fis
yhbE-obgE	Fis	ArcA	CRP
yhbO	MarA	SoxS	Lrp
yhbQ	HcaR	IdnR	NarP
yhbTS	NarL	AraC	CaiF
yhbUV	FhlA	ModE	NarL
yhbW	IHF	FlhDC	MarA
yhbY	CRP	FNR	Fis
yhcAD-ychE_1	AtoC	Cbl	FlhDC

yhcB	AgaR	CpxR	Fur
yhcC	AgaR	CpxR	CsgD
yhcG	AllR	ArgR	CpxR
yhcM	Ada	CaiF	ChbR
yhcN	CRP	IHF	ArcA
yhcO	ArcA	CRP	Cbl
yhdH	CRP	CytR	EnvY
yhdJ	AgaR	AllS	FhlA
yhdN-zntR	CRP	IHF	rpoH
yhdT-panF-prmA	OxyR	Ada	AsnC
yhdV	EnvY	GntR	NagC
yhdWXYZ	NtrC	SdiA	AgaR
yheO-tusDCB	CpxR	rpoH	CRP
yheSTU	ArgP	CpxR	DnaA
yhfG-fic-pabA	Fis	NtrC	CRP
yhfK	AllR	CytR	FlhDC
yhfXW-php-yhfUTS	GntR	NagC	AgaR
yhfZY	GntR	CsgD	FhlA
yhgE	DnaA	FlhDC	FruR
yhgF	CpxR	FNR	Fis
yhgN	AraC	CaiF	CsgD
yhhA	CRP	FNR	Fis
yhhH	AraC	AscG	CpxR
yhhK	AraC	Cbl	CsgD
yhhN	AraC	CynR	IdnR
yhhQ	CpxR	LrhA	MarA
yhhS	BaeR	CpxR	FruR
yhhW	Cbl	CpxR	CysB
yhhX	CpxR	FNR	IHF
yhhY	Fur	GntR	MarR
yhhZ-yrhA-insA-6AB-6B-6	ChbR	CsgD	DicA
yhiI-rbbA-yhhJ	MngR	PhoP	AgaR
yhiJ	Fis	FlhDC	GadE
yhiL	CRP	CpxR	FNR
yhiM	ArcA	CRP	FNR
yhiN	CpxR	FlhDC	FruR
yhiP	CRP	CpxR	LexA
yhiR	ArgR	GntR	LeuO
yhiS	CueR	CytR	DeoR
yhjA	AgaR	Cbl	Fis
yhjD	DeoR	Fis	GadE
yhjE	CRP	Lrp	H-NS
yhjG	AgaR	ArcA	CRP
yhjH	CpxR	FlhDC	H-NS
yhjJ	CpxR	GntR	IscR
yhjK	CpxR	FNR	FruR
yhjQ	CRP	ExuR	Fis
yhjV	BaeR	FNR	Fis
yhjX	CRP	FNR	FhlA
yhjY	PhoB	EvgA	GadX
yiaD	CRP	ArcA	Fis
yiaF	CpxR	FruR	GntR
yiaG	IHF	CRP	Fis
yiaKLMNO-lyxK-sgbHUE	YiaJ	AgaR	CaiF
yiaU	ArgR	CytR	DeoR
yiaWV	FhlA	Fis	FlhDC
yibF	CaiF	DgsA	ExuR
yibJ	FNR	FadR	H-NS
yibK	FadR	FlhDC	GadX

yibN	CpxR	CusR	FNR
yicC	CpxR	FhlA	Fur
yicG	CRP	FNR	IHF
yicH	CpxR	Fis	IHF
yicJI	AgaR	FhlA	GntR
yicL	PhoB	AgaR	Fur
yicR-rpmBG-mutM	FNR	Fis	AgaR
yidE	CpxR	FlhDC	FruR
yidHGF	AraC	FhlA	HyfR
yidKJ	AllR	FlhDC	GntR
yidP	AllR	FlhDC	NagC
yidQ	CRP	CpxR	FNR
yidR	CpxR	IHF	NarL
yieEF	CpxR	rpoH	ArcA
yieG	ArgR	CRP	FNR
yieP-hsrA	rpoH	RcsAB	CRP
yifB	CaiF	Fis	FlhDC
yifE	CRP	CpxR	IHF
yifK	Ada	ArcA	FNR
yifL-dapF-yigA-xerC-yigB	CpxR	rpoH	AllR
yifNO	CRP	CpxR	CsiR
yigGF	H-NS	ArgR	Cbl
yigI	ArcA	CRP	FNR
yigM	AgaR	BetI	DicA
yihA	CpxR	FlhDC	NagC
yihF	Fis	FlhDC	GntR
yihG	CysB	Fis	GlpR
yihI	PurR	CpxR	Fur
yihLM	FhlA	FlhDC	FruR
yihN	AtoC	CaiF	GntR
yihPO	BaeR	FhlA	Fis
yihQ	CaiF	CsgD	MprA
yihUTS	AcrR	AllS	AscG
yihVW	CpxR	GadX	GntR
yihXY-dtd-yiiD	AgaR	BaeR	Cbl
yiiF	CaiF	Cbl	CpxR
yiiQ	SoxS	ArgR	CpxR
yiiX	CpxR	LexA	MetJ
yijE	AllR	AscG	CaiF
yijF	AgaR	AllR	Cbl
yijP	CRP	FNR	Fur
yjaG	ArgR	CpxR	CsgD
yjaH	AcrR	ArgR	CpxR
yjaZ	rpoH	LexA	NtrC
yjbB	AgaR	AraC	AscG
yjbI	AgaR	Fis	GntR
yjbJ	IHF	ArcA	CRP
yjbQR	CRP	FNR	H-NS
yjcB	CRP	ArcA	FNR
yjcC	CpxR	Fur	LexA
yjcD	FNR	Fur	Lrp
yjcE	AtoC	CaiF	DnaA
yjcF	Fis	H-NS	IHF
yjcO	AppY	CaiF	CpxR
yjdA-yjcZ	FlhDC	CpxR	FNR
yjdF	FhlA	AppY	CaiF
yjdIJ	Fis	CRP	FNR
yjdKO	CRP	ChbR	FNR
yjdL	AraC	ArcA	CysB

yjdM	ArcA	CytR	DeoR
yjdN	CRP	Fis	IHF
yjeFE-amiB-mutL-miaA-hfq-hflXKC	CpxR	rpoH	Fis
yjeH	CpxR	GadX	PhoP
yjeI	CpxR	FlhDC	H-NS
yjeJ	DsdC	EvgA	Fis
yjeK	FlhDC	ArgR	BaeR
yjeM	Cbl	LacI	PhoB
yjeNO	ArgP	GntR	IdnR
yjeS	CpxR	DnaA	Fis
yjfIJ	AgaR	AllS	CsgD
yjfN	CRP	CpxR	FNR
yjfO	CRP	FNR	Fis
yjfP	CsgD	AraC	CaiF
yjfY	AllS	CRP	CpxR
yjfZ	CdaR	FlhDC	Fur
yjgA	FNR	Fis	IHF
yjgB	MarA	SoxS	NarL
yjgF	CRP	FNR	IHF
yjgH	PhoB	ArcA	Cbl
yjgI	AgaR	AscG	CsgD
yjgK	CRP	FNR	HcaR
yjgN	GntR	H-NS	LexA
yjgPQ	CpxR	ArgR	DicA
yjgR	DicA	GadX	HU
yjgW	CdaR	H-NS	LexA
yjgZ	AraC	CsgD	EnvY
yjhBC	CRP	FNR	FlhDC
yjhF	Cbl	FlhDC	MetR
yjhIHG	ArgR	CRP	CusR
yjhP	AtoC	Cbl	CysB
yjhR	LexA	Fur	NagC
yjhS	NagC	CdaR	CysB
yjhU	CpxR	DicA	FlhDC
yjhXQ	Rob	Cbl	MprA
yjiC	AgaR	RcsAB	TdcA
yjiD	FNR	Fur	IHF
yjiE	BaeR	FhlA	GlcC
yjiJ	DnaA	FlhDC	GntR
yjiK	DgsA	FlhDC	GntR
yjiML	Rob	ArcA	Cbl
yjiPQ	ArgP	ArgR	CpxR
yjiR	Cbl	DgsA	GntR
yjiT	H-NS	IHF	CpxR
yjiXA	CRP	FNR	AcrR
yjjB-dnaTC-yjjA	CpxR	FlhDC	LexA
yjjI	NarL	FNR	IHF
yjjJ	AscG	CpxR	HyfR
yjjK	CRP	CpxR	FNR
yjjL	CpxR	FlhDC	Fur
yjjM	CRP	CpxR	FNR
yjjP	CusR	FlhDC	GntR
yjjQ-bglJ	Fur	Fis	IHF
yjjUV	Lrp	CRP	H-NS
yjjW	FhlA	LldR	ModE
yjjX	DnaA	HyfR	MarA
yjjY	FNR	CRP	CpxR
yjtD	AgaR	BetI	CpxR
ykfA-yafZ	FlhDC	AllR	DicA

ykfC	ArgR	Cbl	DeoR
ykfJ-prfH	FadR	GntR	IdnR
ykgA	AllR	CdaR	FNR
ykgC	AscG	CpxR	EvgA
ykgD	CsgD	GntR	GutM
ykgEFG	CRP	FNR	IHF
ykgH	AgaR	GntR	HcaR
ykgIB	TdcA	TdcR	CRP
ylaB	CpxR	CytR	H-NS
ylaC	ArcA	CRP	FNR
yliEF	CpxR	CsgD	DgsA
yliG	CRP	Fis	IHF
yliI	ArgR	GntR	IscR
yliJ	FruR	Nac	NtrC
yliL	CRP	CsgD	Fis
ymbA	CaiF	CdaR	DgsA
ymcE-gnsA	AraC	ArcA	CRP
ymdA	DeoR	FNR	FlhDC
ymdBC	FlhDC	ArgR	CdaR
ymdE-ycdU	CpxR	CsgD	FhlA
ymfA-ycfZ	UxuR	Cbl	CpxR
ymfED	CRP	CysB	FNR
ymfH-xisE-intE	ArcA	CRP	H-NS
ymfI	CRP	CdaR	FNR
ymfJ	AllS	ArcA	CRP
ymfK	CpxR	FNR	Fur
ymfTLMNROPQ-ycfK-ymfS	LexA	CRP	AgaR
ymgA-ariR	IHF	CRP	CpxR
ymgE	CRP	Fis	IHF
ymgGD	FruR	CRP	DhaR
ymjA	FNR	FhlA	Fis
ymjC-mpaA	CpxR	CytR	DeoR
ynaA-lomR_1	FlhDC	ArgR	CpxR
ynaE	ArcA	CRP	FNR
ynaI	CpxR	CRP	Fis
ynaJ	FNR	ArcA	CRP
ynaK-ydaY	FlhDC	AgaR	Fur
ynbABCD	GntR	RcsAB	CpxR
yncA-ydcZ	CpxR	H-NS	FadR
yncB	CRP	Fis	H-NS
yncC	CRP	Fis	PhoB
yncE	CRP	Fur	FNR
yncG	CpxR	Fur	IdnR
yncI	CRP	CpxR	FNR
yncJ	CpxR	IscR	ModE
yncM	ArcA	GntR	IscR
yncN-ydcQ	DicA	GcvA	LexA
yneHG	MarA	SoxS	ExuR
yneI	CpxR	FNR	FruR
yneJ	GadX	MetJ	NagC
yneK	CpxR	FlhDC	IscR
yneL	CpxR	FlhDC	GntR
yneN-ydeO	CpxR	FlhDC	CRP
ynfA	Cbl	CdaR	DeoR
ynfB-speG	CRP	FNR	ArcA
ynfC	CRP	CpxR	DeoR
ynfD	NtrC	PhoB	Fis
ynfEFGH-dmsD	FNR	NarL	IHF
ynfM	FNR	GadE	IHF

ynfN	Cbl	CdaR	CpxR
ynfO-ydfO	CRP	FNR	FhlA
ynhG	CRP	IHF	Fis
yniA	CRP	CpxR	Fis
yniB	AraC	CaiF	Fis
yniC	Cbl	FhlA	Fis
ynjE	ArcA	FNR	IHF
ynjF	CRP	FlhDC	IHF
ynjH	CaiF	CpxR	FNR
ynjI	CpxR	FruR	Fur
yoaA	FNR	HcaR	ModE
yoaB	CRP	Fis	Fur
yoaC	H-NS	CRP	Fis
yoaD	CaiF	DhaR	GntR
yoaE	ExuR	FlhDC	Fur
yoaF	AgaR	BetI	FlhDC
yoaH	Ada	CaiF	MarR
yoaI	CpxR	FhlA	ModE
yobA-yebZY	Fis	CRP	H-NS
yobB-exoX	NagC	CpxR	CusR
yobD	BaeR	CpxR	FlhDC
yobF-cspC	FNR	CRP	CpxR
yodC	ArcA	CRP	IHF
yodD	Fis	CRP	FruR
yoeE	ArgR	CaiF	Cbl
yohC	CRP	DsdC	FNR
yohD	SoxS	FadR	FruR
yohF	SoxS	H-NS	MarA
yohJK	Ada	GadW	MtlR
yohN	ArgR	FlhDC	H-NS
yojI	Fur	CRP	FNR
ypdABC	PhoB	SoxS	DicA
ypeA-yfeZ	AraC	ArgR	CpxR
ypeC	CpxR	FlhDC	Fur
ypfG	DicA	ModE	NagC
ypfH	NagC	NarL	PhoB
ypfJI	PhoB	Cbl	FlhDC
yphA	DeoR	DgsA	Fis
yphCB	Rob	Cbl	CdaR
yphFED	GntR	Cbl	CusR
yphG	FadR	GntR	NagC
yphH	Ada	DeoR	GntR
ypjC	CpxR	Fis	HyfR
ypjD	ArgR	CpxR	Nac
ypjL-yfjU	CaiF	FhlA	GlcC
ypjM-yfjV	AcrR	ArgR	FlhDC
yqaBA	ArgR	CpxR	Fis
yqaD	ArgR	BetI	GntR
yqaE	CRP	EvgA	FNR
yqcC-truC-yqcA	AppY	CpxR	GntR
yqcE-ygcE	NagC	CpxR	CsgD
yqeA	AllS	CaiF	ChbR
yqeB	GalR	GalS	GntR
yqeC	CRP	CpxR	GlpR
yqeF	Fis	ArcA	CRP
yqeG	CRP	CpxR	IHF
yqfA	CRP	CpxR	Fis
yqgA	AgaR	CaiF	FhlA
yqgD	Fis	FlhDC	IHF

yqgEF	IclR	AllS	CpxR
yqhA	ArcA	ArgR	FNR
yqhC	CpxR	MarA	QseB
yqhD	ArcA	CRP	FNR
yqhG	AgaR	FlhDC	GntR
yqiC	ArcA	CRP	FNR
yqiJK	AgaR	CRP	CpxR
yqjAB	CpxR	Fis	ArgR
yqjCDEK	Fis	IHF	MarA
yqjF	AgaR	CpxR	FruR
yqjG	CRP	IHF	NtrC
yqjH	CRP	FNR	Fur
yqjI	FNR	ArcA	CRP
yraH	AgaR	ArgR	Cbl
yraIJK	AgaR	CsgD	HU
yraL	BaeR	CsiR	FhlA
yraM	CpxR	FlhDC	Lrp
yraN	AllS	LldR	PhoP
yraP	CpxR	Fis	FlhDC
yraQ	CdaR	CueR	Fis
yraR	AppY	CynR	DeoR
yrbA	Fis	CRP	CpxR
yrbFEDCB	Fis	rpoH	FNR
yrbG-kdsD	CpxR	Fis	Fur
yrbL	PhoP	CRP	CpxR
yrdA	AraC	CRP	CpxR
yrdD-rimN-aroE-yrdB	CpxR	FNR	H-NS
yrfF	FruR	LexA	MhpR
yrfG-hslRO	rpoH	CRP	Fis
yrhB	AraC	LexA	Lrp
ysaA	NarL	ExuR	FNR
ysgA	FNR	Lrp	CRP
ytfB	CRP	CpxR	ExuR
ytfE	CRP	FNR	Fis
ytfG	AraC	AscG	CaiF
ytfI	AllS	CRP	ChbR
ytfJ	CRP	Fis	H-NS
ytfK	FNR	ArcA	CRP
ytfL	FadR	LexA	Rob
ytfMNP	CpxR	ArgP	ArgR
ytfQRT-yjfF	CRP	Fis	GntR
ytjB-lplA	AraC	AppY	ArgR
ytjC	CpxR	FadR	MarA
yzcX	CsgD	Fis	MarA
yzfA	AcrR	AgaR	CsgD
zapA	ArgR	CRP	CpxR
zinT	Fur	SoxS	CRP
zipA	ArgR	FNR	Fis
zitB	CytR	DeoR	FlhDC
zntA	CpxR	Fis	IHF
znuA	Zur	CRP	FNR
znuCB	Zur	CaiF	CdaR
zraSR	Cbl	AgaR	AppY
zupT	CpxR	FhlA	Fis
zur	CpxR	CytR	FruR
zwf	MarA	Rob	SoxS
